# Distinct mechanisms of recognition of phosphorylated RNAPII C-terminal domain by BRCT repeats of the BRCA1–BARD1 complex

**DOI:** 10.1016/j.jbc.2025.111010

**Published:** 2025-12-05

**Authors:** V. Klapstova, K. Sedova, J. Houser, M. Sebesta

**Affiliations:** 1 CEITEC–Central European Institute of Technology, Masaryk University Brno, Czechia; 2 National Centre for Biomolecular Research, Faculty of Science, Masaryk University Brno, Czechia

**Keywords:** BRCA1, BRCT repeats, DNA repair, transcription, RNA polymerase II, condensation

## Abstract

Transcription competes with other DNA-dependent processes, such as DNA repair, for access to its substrate, DNA. However, the principles governing the interplay between these processes remain poorly understood. Evidence suggests that the BRCA1-BARD1 complex, a key player in the DNA damage response, may act as a mediator of this crosstalk. In this study, we investigated the molecular mechanism underpinning the interaction between RNA polymerase II (RNAPII) and the BRCA1-BARD1 complex, as well as its functional implications. Our findings reveal that the tandem BRCT domain of BRCA1 binds the Ser5-phosphorylated CTD of RNAPII, utilizing a mechanism previously established for other BRCA1 BRCT ligands. Furthermore, we demonstrate that this interaction is critical for the organization of RNAPII into condensates with liquid-like properties. Analysis of disease-associated variants within the BRCT repeats further supports the biological relevance of this condensation. Collectively, our results suggest that the BRCA1-BARD1 complex may coordinate transcription and DNA repair by facilitating the organization of RNAPII into transcription factories.

Accurate transmission of genetic information is paramount for cellular viability. Since DNA functions as a template for replication, recombination, repair, and transcription simultaneously, these processes can intersect, potentially leading to genomic instability (1, 2, 3, 4). One of the major sources of conflict arises from the formation of R-loops, tripartite nucleic-acid structures in which the nascent RNA hybridizes with its complementary DNA strand, leaving the nontemplate strand unpaired. R-loops can tether RNA polymerases (RNAPs) to chromatin, thereby increasing the risk of collisions with replication and repair complexes (5, 6, 7, 8). To mitigate these transcription–replication/repair conflicts, cells use nucleases (*e*.*g*., RNase H) and helicases (*e*.*g*., senataxin and members of the DDX family) to dismantle R-loops (9, 10, 11, 12, 13, 14, 15, 16) and factors such as XRN2 and senataxin to promote the dislodging of RNAPs from chromatin (17, 18).

Although transcription can threaten genome stability by generating R-loops, it also plays a beneficial role in DNA repair. For instance, RNA polymerase II (RNAPII) activity is critical for efficient repair of DNA double-stranded breaks (DSBs) *via* homologous recombination (HR) (19, 20, 21, 22, 23). Current models propose that RNAPII produces damage-specific short RNAs at DSB sites to activate the DNA damage response, thereby promoting repair (24, 25). The kinase c-ABL phosphorylates the C-terminal domain (CTD) of the catalytic subunit of RNAPII to facilitate this damage-induced transcription (26, 27), and once the RNAPII activity is no longer required, the phosphatase PP2A and helicase senataxin are recruited *via* INTS6 to remove RNAPII and enable efficient DSB repair (28).

The presence of RNAPII at DSBs raises the question of how its activity is coordinated with the early steps of HR. A likely candidate for mediating this crosstalk is the BRCA1–BARD1 complex, known to associate with transcriptionally engaged RNAPII (29, 30, 31, 32, 33, 34)–possibly *via* its BRCT repeats (33, 35). The BRCT domain of BRCA1 recognizes a diverse set of proteins containing phosphorylated serine followed by an aromatic residue (tyrosine or phenylalanine) at the +3-position relative to the phosphoserine (33, 36, 37, 38, 39, 40, 41). The binding pocket recognizing the phosphorylated serine is conserved also in the BRCT domain of BARD1, however, its preferred binding motif has yet to be structurally characterized (33, 42, 43).

The BRCA1-BARD1 complex is essential for HR, in which it promotes pathway choice (by antagonizing the 53BP1-mediated nonhomologous end-joining pathway) and stimulates RAD51 filament formation required for homology search (44, 45, 46, 47, 48, 49, 50). It also ubiquitinates chromatin to activate the DNA damage response (51, 52) and modulates DNA end processing enzymes (53, 54, 55), placing the BRCA1-BARD1 complex at a prime position to promote the crosstalk between transcription and HR.

Recent studies suggest that both transcription factors (56, 57, 58, 59, 60, 61, 62, 63, 64, 65) and DNA repair factors (24, 66, 67, 68, 69, 70, 71) can assemble into membraneless factories, potentially *via* liquid-liquid phase separation (LLPS). Such clustering may enhance the efficiency and spatial organization of nuclear processes. Nevertheless, how specificity and selectivity in these condensates is achieved remains poorly understood, particularly regarding the interplay between transcription and HR machinery.

Here, we provide the first detailed investigation of direct interactions between RNAPII and the BRCA1–BARD1 complex at molecular level. Using biochemical and structural analyzes, we show that the tandem BRCT domains of BRCA1 and BARD1 differ in their binding to the CTD of RNAPII. We further present the first three-dimensional structure of a tandem BRCT domain bound to a phosphorylated on serine 5 (pS5-CTD) peptide, revealing how this binding promotes condensation of the phosphorylated CTD (pCTD) in an interaction-dependent manner. Finally, we demonstrate that disease-associated variants within the BRCT repeats impair LLPS *in vitro*, thereby providing a possible mechanistic insight of the defects associated with these variants, underscoring the functional relevance of the condensation of the BRCA1–BARD1 complex in the presence of the pCTD of RNAPII. These findings provide new insights into how transcription is coordinated with early HR events.

## Results

### The BRCA1-BARD1 complex interacts with the RNAPII CTD through its BRCT repeats

Ample evidence supports the association of the BRCA1-BARD1 complex with transcriptionally engaged RNAPII (29, 30, 72, 73). However, the possibility of a direct, physical interaction between these complexes has not been investigated.

**Figure 1:**
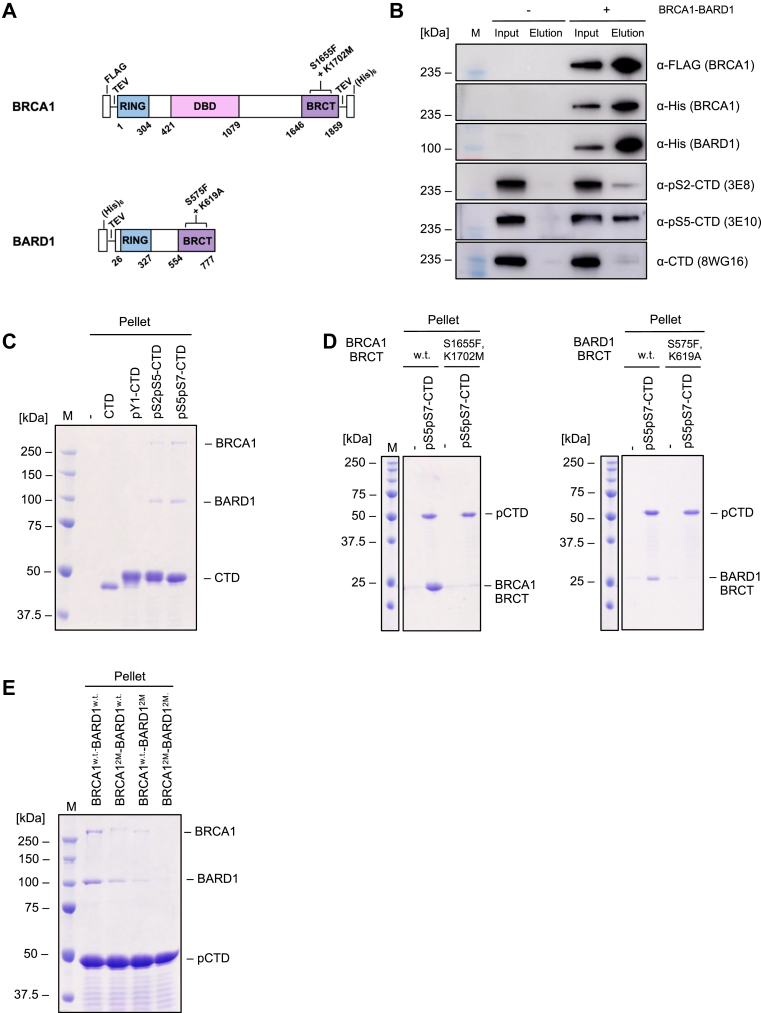
**The interaction between the BRCA1-BARD1 complex and RNAPII is direct and mediated by the phosphorylated CTD of RNAPII and the BRCT domains of BRCA1-BARD1**. *A*, schematic representation of the domains of BRCA1 and BARD1. Positions of investigated binding variants, the tags, and cleavage sites are indicated. *B*, western blot analysis of pull-downs from HEK293 lysates. HEK293 cells were lysed, and the lysate was cleared by centrifugation. To the supernatant, FLAG-BRCA1-BARD1 was added, and the samples were incubated with α-FLAG beads. As a control, the HEK293 lysate with no added BRCA1-BARD1 was used. The proteins were eluted using 3xFLAG peptide and the samples were analyzed using western blots. BRCA1-BARD1 interacts with RNAPII *via* the CTD phosphorylated on Ser2 and Ser5, respectively. Uncropped blot and gel images are provided in Figure S2. *C*, SDS-PAGE analysis of *in vitro* pull-down assay between GST-(CTD)_26_ and the BRCA1-BARD1 complex. Purified BRCA1-BARD1 was incubated with phosphorylated and nonphosphorylated GST-(CTD)_26_ bound to glutathione beads. The samples were centrifuged and the input, unbound (supernatant) and bound (pellet) fractions were analyzed using the SDS PAGE. BRCA1-BARD1 interacts directly with GST-pS2pS5-(CTD)_26_ and GST-pS5pS7-(CTD)_26_*in vitro*. Uncropped gel images are provided in Figure S3*A*. *D*, SDS-PAGE analysis of *in* vitro pull-down assay between GST-pS5pS7-(CTD)_26_ and BRCA1 BRCT and BARD1 BRCT, respectively. Purified BRCA1 BRCT and BARD1 BRCT, respectively, were incubated with GST-pS5pS7-(CTD)_26_ bound to glutathione beads. The samples were centrifuged and the input, unbound (supernatant) and bound (pellet) fractions were analyzed using the SDS PAGE. Substitutions in the phosphoserine binding site (BRCA1^S1655F,K1702M^, BARD1^S575F,K619A^) abolish the binding. Uncropped gel images are provided in Figure S3*B*. *E*, SDS-PAGE analysis of *in vitro* pull-down assay between GST-pS5pS7-(CTD)_26_ and BRCA1-BARD1 variants BRCA1^w.t.^-BARD1^w.t.^ (BRCA1-BARD1), BRCA1^2M^-BARD1^w.t.^ (BRCA1^S1655F,K1702M^-BARD1), BRCA1^w.t.^-BARD1^2M^ (BRCA1-BARD1^S575F,K619A^), and BRCA1^2M^-BARD1^2M^ (BRCA1^S1655F,K1702M^- BARD1^S575F,K619A^), respectively. Purified BRCA1-BARD1 variants were incubated with GST-pS5pS7-(CTD)_26_ bound to glutathione beads. The samples were centrifuged and the input, unbound (supernatant) and bound (pellet) fractions were analyzed using the SDS PAGE. The inactivation of single tandem BRCT repeat (either BRCA1 or BARD1 BRCT, respectively) lead to the reduction of binding. Uncropped gel images are provided in Figure S3*C*. CTD, C-terminal domain; RNAPII, RNA polymerase II.

To address this, we purified the BRCA1-BARD1 complex to near-homogeneity from insect cells using a combination of affinity chromatography and size-exclusion chromatography (Fig. 1*A*, Fig. S1, *A* and *B*). We first tested whether the BRCA1-BARD1 complex, immobilized on FLAG beads, could pull down RNAPII from HEK293T cell extract. The BRCA1-BARD1 complex pulled-down RNAPII with a pS5-CTD (Fig. 1*B*, Fig. S2). Next, we tested the ability of purified GST-(CTD)_26_ peptides – specifically phosphorylated on Y1, S2, and S5 by ABL1, DYRK1a, and the CDK7 complex, respectively (70)—to pull down the BRCA1-BARD1 complex. *In vitro*, the complex directly, and specifically, recognized CTD phosphorylated on S2 and S5 (Fig. 1*C*, Fig. S3*A*). Further analysis showed that this interaction is mediated by the BRCT domains, *via* the conserved residues S1655 and K1702 in BRCA1, and S575 and K619 in BARD1, which have been implicated in phospho-serine recognition (33, 35, 39, 74, 75) (Fig. 1*D*, Fig. S3*B*). To identify, which of the two BRCT domains is responsible for the interaction with pCTD, we prepared variants of the BRCA1-BARD1 complex harboring substitutions abolishing the interaction with the phosphorylated serine (BRCA1^S1655F,K1702M^ (BRCA1^2M^), BARD1^S575F,K619A^ (BARD1^2M^)) in either one or both of the BRCT domains (BRCA1^2M^-BARD1^w.t.,^ BRCA1^w.t.^-BARD1^2M^, and BRCA1^2M^-BARD1^2M^, respectively). Already, inactivation of single tandem BRCT repeat leads to the reduction of binding, suggesting that both domains are required for efficient recognition of pCTD by the BRCA1-BARD1 complex (Fig. 1*E*, Fig. S3*C*).

In summary, these data indicate that the BRCA1-BARD1 complex directly interacts with the pCTD of RNAPII. This interaction is mediated by the BRCT repeats on both subunits, and the mechanism of phospho-serine recognition appears similar to that proposed for other known BRCT ligands.

### The BRCT domains of BRCA1 and BARD1, respectively, differ in binding kinetics to the pCTD

**Figure 2:**
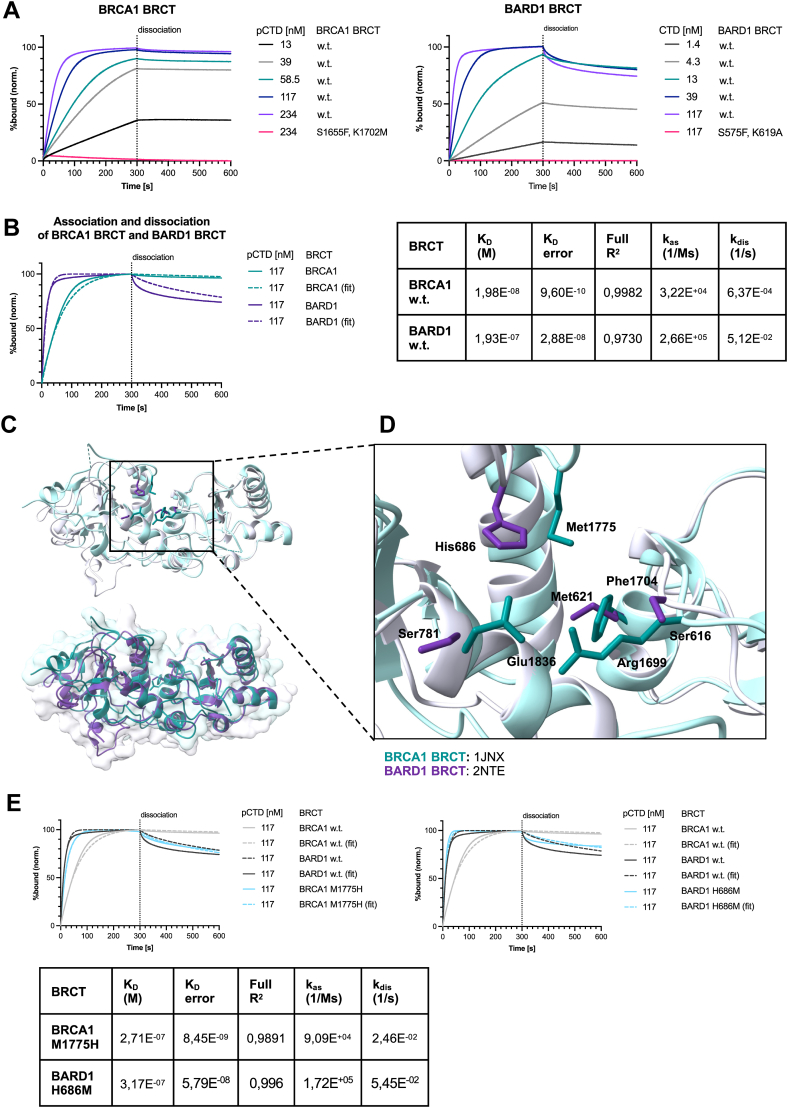
**The BRCT domains of the BRCA1–BARD1 complex exhibit differences in their binding kinetics to the phosphorylated CTD of RNAPII**. *A*, sensorgrams obtained by biolayer interferometry (BLI) demonstrating interactions of BRCA1 BRCT and BARD1 BRCT with GST-pS5pS7-(CTD)_26_. BRCA1 BRCT and BARD1 BRCT interact with the GST-pS5pS7-(CTD)_26_*via* the canonical phosphoserine binding site. Substitutions in the phosphoserine binding site (BRCA1 BRCT ^S1655F,K1702M^, BARD1 BRCT ^S575F,K619A^) abolish the binding. The sensorgrams represent the mean of three measurements for each concentration. The data were analyzed in Octet Analysis Studio Software using 1:2 Bivalent analyte model. The data were plotted using Prism GraphPad 9 software. Sensorgrams for individual concentrations of BRCA1 BRCT ^S1655F, K1702M^, BARD1 BRCT ^S575F, K619A^ can be found in Figure S6*A*. *B*, sensorgrams obtained by biolayer interferometry (BLI) and their respective fits (*left*). Comparison of association (*k*_as_) and dissociation (*k*_dis_) kinetic constants, and equilibrium dissociation (*K*_D_) constants for GST-pS5pS7-(CTD)_26_ and BRCA1 and BARD1 BRCT, respectively, obtained by biolayer interferometry (*right*). BARD1 BRCT associates with GST-pS5pS7-(CTD)_26_ more dynamically than BRCA1 BRCT. The sensorgrams represent the mean of three measurements for each concentration. The association and dissociation constants and the coefficient of determination (R^2^) indicating the appropriateness of the fit were calculated in Octet Analysis Studio Software using 1:2 Bivalent analyte model. The data were plotted using Prism GraphPad 9 software. *C*, structural alignment of BRCA1 BRCT (PDB: 1JNX, *teal*) and BARD1 BRCT (PDB: 2NTE, *purple*) obtained in UCSF Chimera. *D*, comparison of the amino-acid composition of the hydrophobic pocket of BRCA1 BRCT (1JNX, *teal*) and the residues present on the homologous positions in BARD1 BRCT (2NTE, *purple*). Close up from (*C*). *E*, comparison of sensorgrams obtained by biolayer interferometry (BLI) and their respective fits (*top*) of GST-pS5pS7-(CTD)_26_ binding to BRCA1 BRCT^M1775H^ and BARD1 BRCT^H686H^. Kinetic parameters (association (*k*_as_), dissociation (*k*_dis_), and dissociation (*K*_D_) constants) (*bottom*). The sensorgrams represent the mean of three measurements for each concentration. The data were analyzed in Octet Analysis Studio Software using 1:2 Bivalent analyte model. The data were plotted using Prism GraphPad 9 software. Sensorgrams for individual concentrations can be found in Figure S6*C*. CTD, C-terminal domain; PDB, Protein Data Bank; RNAPII, RNA polymerase II.

In our initial *in vitro* binding assay, we observed a clear difference in the amount of pulled-down BRCT domain (Fig. 1*D*). To investigate this difference, we measured the binding kinetics between the isolated BRCT domains and pCTD using biolayer interferometry (BLI). The data revealed an apparent affinity of 20 nM for BRCA1 BRCT and 193 nM for BARD1 BRCT (Fig. 2*A*, Fig. S6*A*). Control experiments showed that the variants of BRCT domains defective in recognition of phosphopeptide (BRCA1 BRCT^S1655F,K1702M^ and BARD1 BRCT^S575F,K619A^) did not bind pCTD, nor did the wild-type BRCT domains bind unmodified CTD, consistent with the *in vitro* pull-down assays described above.

The binding profile suggests that the BARD1 BRCT exhibits more dynamic binding, as it associates and dissociates faster from the hyperphosphorylated CTD (Fig. 2*B*). To investigate this phenomenon from structural point of view, we aligned the existing apo structures of the BRCT domains (Protein Data Bank (PDB) codes: 1JNX, 2NTE) (43, 76) (Fig. 2, *C* and *D*, Fig. S6*B*). This structural alignment revealed that the residues recognizing the phosphoserine are conserved in both BRCA1 and BARD1; however, the surrounding residues, which interact with the broader peptide sequence differ between these domains. BRCA1 BRCT recognizes its ligands *via* a two-anchor mechanism, in which one anchor is the phosphorylated serine recognized by S1655 and K1702, and the second is an aromatic residue at the +3 position relative to the phosphoserine, which fits into a hydrophobic pocket composed of T1700, L1701, F1704, N1774, M1775, and L1839, with R1699 contributing by additional stabilizing hydrogen bonds (33, 36, 37, 38, 39, 40, 41). Within the context of the CTD, this aromatic residue is Y1. As the hydrophobic pocket of BARD1 BRCT is shallower—due to the presence of histidine 686 (H686) instead of methionine 1775 (M1775) as in BRCA1–this may affect the accommodation of the Y1 residue of the CTD. To test this hypothesis, we engineered BRCA1 BRCT^M1775H^ and BARD1 BRCT^H686M^ variants to swap these key residues (Fig. 2*E*, Fig. S6*C*). BRCA1 BRCT^M1775H^ exhibited binding kinetics similar to BARD1 BRCT, supporting our model. However, the mirror variant (BARD1 BRCT^H686M^) retained a more dynamic binding profile, suggesting that additional substitutions are necessary to reconstitute a fully BRCA1-like hydrophobic pocket (Fig. 2, *C* and *D*).

In summary, the kinetic binding assays show that the two BRCT domains within the BRCA1-BARD1 complex differ in recognizing the Y1 of the pCTD.

### The mechanism of BRCT domain binding to a pS5-CTD diheptad

**Figure 3:**
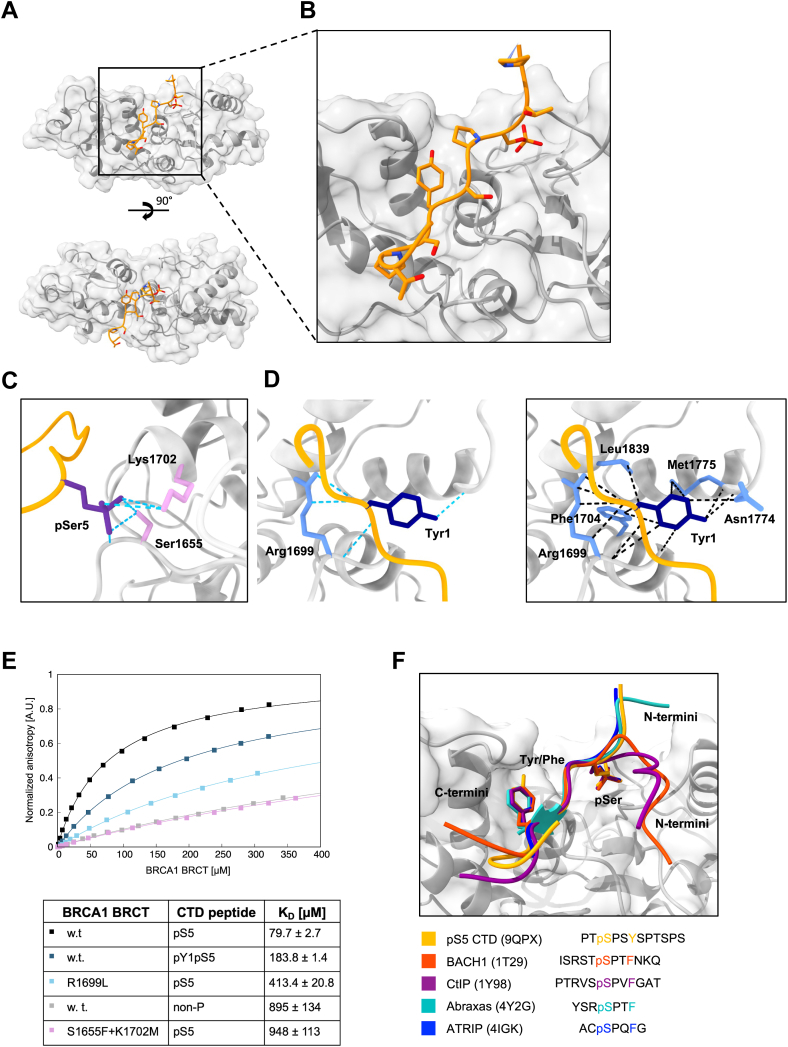
**Structural characterization of the BRCA1 BRCT domain bound to pS5 CTD peptide**. *A*, crystal structure of BRCA1 BRCT (*gray*) with bound pS5 CTD peptide (*yellow*), PDB ID: 9QPX. The data collection and refinement statistics is provided in (Table S4). *B*, detail of the BRCA1 BRCT phospho-peptide binding site (*gray*) with bound pS5 CTD peptide (*yellow*). Close up from (*A*). *C*, detail of the phosphoserine binding site of BRCA1 BRCT (*gray*) with bound pS5 CTD peptide (*yellow*). The pS5 of the CTD peptide is depicted in *purple*, the amino-acid residues interacting with pS5 are depicted in *light violet*. The hydrogen bonds were displayed as pseudo bonds using the structural analysis tool Hydrogen bonds in UCSF ChimeraX and are depicted in *turquoise*. The relax distance tolerance was 1.0 Å and the relax angle tolerance was 20.0. *D*, detail of the aromatic amino-acid binding pocket of BRCA1 BRCT (*gray*) with bound pS5 CTD peptide (*yellow*). The Y1 of the CTD is depicted in *navy blue*, the interacting amino-acid residues in *light blue*. The hydrogen bonds (*left*) were displayed as pseudobonds using the structural analysis tool Hydrogen bonds in UCSF ChimeraX and are depicted in *turquoise*. The relax distance tolerance was 1.0 Å and the relax angle tolerance was 20.0. van der Waals hydrophobic and stacking interactions (*right*) were displayed as pseudobonds using the structural analysis tool in UCSF ChimeraX and are depicted in *black*. The interacting atoms were identified based on van der Waals overlap ≥ −0.4 Å. *E*, validation of the obtained structural model by fluorescence anisotropy measurement. The assays were performed between BRCA1 BRCT domain and the indicated ligands (at 25 nM). Anisotropy data were plotted as a function of protein concentration and fitted to a single-site saturation with nonspecific binding model using XMGrace. *F*, comparison of binding of different ligands to BRCA1 BRCT. Alignment of the structures was created using UCSF ChimeraX. BRCA1 BRCT (from the structure with pCTD, PDB ID: 9QPX) is depicted in *gray*, pCTD peptide in *yellow*, BACH1 phosphopeptide (PDB: 1T29) in *red*, CtIP phosphopeptide (PDB: 1Y98) in *magenta*, Abraxas singly phosphorylated peptide (PDB: 4Y2G) in *turquoise*, ATRIP phosphopeptide (PDB: 4IGH) in *blue*. pCTD, phosphorylated CTD; PDB, Protein Data Bank; pS5-CTD, phosphorylated on serine 5-CTD.

The specific binding of the BRCA1 and BARD1 BRCTs toward the hyperphosphorylated CTD prompted structural characterization of the binding *via* X-ray crystallography. For both BRCA1 and BARD1 BRCT domains, we obtained ligand-free structures at resolution of 2.4 Å and 1.8 Å, respectively. These apo structures were nearly identical to the existing ones; therefore, we did not analyze them further. Next, we focused on mixtures of BRCT domains with peptides derived from the consensus CTD sequence—modified specifically on either S5 alone or on S2, S5, and S7. No additional density corresponding to the CTD-derived peptide was observed in crystals formed by the BARD1 BRCT domain when mixed with either pS5-CTD or pS2pS5pS7-CTD. In contrast, clear density corresponding to the pS5-CTD-derived peptide was observed in the BRCA1 BRCT dataset, despite the BRCA1 BRCT domain exhibiting similar affinities for both peptides (Fig. S7*A*). The BRCA1 BRCT-CTD complex crystallized in the C222_1_ space group with four monomers in the asymmetric unit, and the structure was refined to 2.8 Å resolution (Table S4). Up to eight of the 14 residues of the pS5-CTD peptide could be fitted into the extra density (Fig. 3*A*; Fig. S7, *B*–*E*). Structural analysis revealed that the CTD ligand was accommodated within the positively charged binding pocket of the BRCA1 BRCT domain without inducing conformational changes to the overall domain fold. Indeed, structural alignment of the apo and liganded forms indicated no induced-fit movements (Fig. S7).

Closer examination revealed that the pS5 CTD ligand is bound *via* the canonical two-anchor mechanism, consistent with other known BRCA1 BRCT ligands (Fig. 3, *A* and *B*, *F*) (38, 40, 41, 77, 78). Specifically, pS5 from the first CTD repeat is recognized by S1655 and K1702, and the Y1 from the next CTD repeat occupies a hydrophobic pocket formed by R1699, F1704, N1774, M1775, and L1839. Moreover, the P6 residue from the first CTD repeat contributes to this interaction, aligning with the canonical pSPXY/F binding motif seen in other BRCA1 BRCT ligands (Fig. 3, *C*–*E*) (33).

We corroborated these X-ray data by mutational analysis using fluorescence anisotropy measurements with a pS5-(CTD)_2_ peptide. Replacing S1655 and K1702 reduced BRCA1 BRCT affinity to 895 ± 134 μM, similar to the affinity of the w.t. domain for the nonphosphorylated (CTD)_2_ (K_D_ = 948 ± 113 μM). Comparable results were obtained for the BARD1 BRCT^M1775K^ variant, confirming that M1775 is essential for accommodating the aromatic residue at position +3. Likewise, using a pY1pS5-(CTD)_2_ peptide and the R1699L BRCA1 variant (which disrupts the hydrophobic pocket), respectively, weakened binding to the pCTD (K_D_ = 183.8 ± 1.4 μM and 413.4 ± 20.8 μM, respectively) (Fig. 3*E*).

As BARD1 BRCT cocrystallization with pCTD was unsuccessful we used AlphaFold 3 (79) (Fig. S8, *A* and *B*, Fig. S29, *A*–*D*) to predict how BARD1 BRCT might bind pS5-(CTD)_2_. The resulting model aligned well with our mutational data, suggesting that S575 and K619 indeed recognize pS5 in the CTD and that H686, S616, M621, P687, N690, and I741 interact with Y1 in the CTD (Fig. S8, *C* and *D*).

In summary, these structural analyzes clarify why the BRCA1 BRCT domain exhibits a preference for pS5-CTD and suggest a similar canonical binding mode for BARD1 BRCT domain.

### The BRCA1-BARD1 complex forms condensates *in vitro* into which it can simultaneously incorporate hyperphosphorylated CTD and an RNA transcript

Our next question concerned the functional significance of BRCA1-BARD1 interacting with hyperphosphorylated CTD. Recent work has shown that phosphorylated RNAPII can cluster into transcription-cycle-specific condensates with liquid-like properties *in vivo* (57, 62, 80, 81, 82, 83), and the hyperphosphorylated form may shuttle as a client (62, 84). In addition, the BRCA1-BARD1 complex has also been suggested to be present in condensates *in vivo* (85). We therefore asked whether the purified BRCA1-BARD1 complex can undergo condensation *in vitro*.

**Figure 4:**
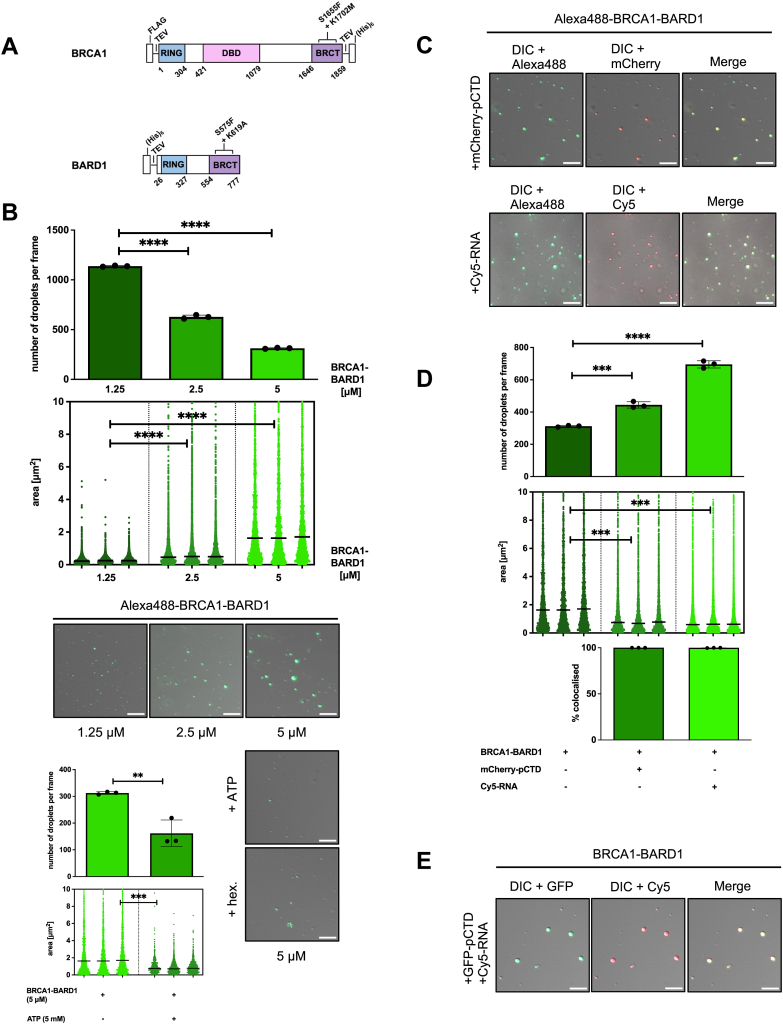
**The BRCA1-BARD1 complex forms liquid-like condensates *in vitro*, which accommodate phosphorylated CTD domain of RNAPII and RNA**. *A*, schematic representation of BRCA1 and BARD1 domains. Positions of investigated binding mutants are indicated. *B*, liquid-liquid phase separation (LLPS) assays with purified, Alexa488-labeled BRCA1-BARD1. BRCA1-BARD1 (at 1.25 μM, 2.5 μM, and 5 μM) was mixed with the crowding agent (10% dextran). Bar chart (*top*) representing quantification (n = 3) of the number of droplets per frame from the LLPS experiments with BRCA1-BARD1. Statistical significance was determined by unpaired *t* test. A nested scatterplot (*middle*) represents quantification (n = 3) of an area of individual droplets from three independent experiments with BRCA1-BARD1, with median area determined per dataset. Statistical significance was determined by nested *t* test. Representative images from three experiments (*bottom*) are depicted as an overlay of differential interference contrast (DIC) and GFP. Where indicated, hexane-1,6-diol (hex; at 10%) was added to inhibit hydrophobic interactions or ATP (at 5 mM) to inhibit electrostatic interactions. The scale bar represents 10 μm. *C*, LLPS assays with purified, Alexa488-labeled BRCA1-BARD1, mCherry-p-hCTD and Cy5-RNA. BRCA1-BARD1 (at 5 μM) was mixed with phosphorylated CTD (2.5 μM), and Cy5-ITS1 RNA (at 15 nM), respectively, in the presence of a crowding agent (10% dextran). Representative images from three experiments are depicted as an overlay of differential interference contrast (DIC), Alexa488, and Cy5. The scale bar represents 10 μm. *D*, bar chart (*top*) representing quantification (n = 3) of the number of droplets per frame from the LLPS experiments with the BRCA1-BARD1 complex shown in (*C*). Statistical significance was determined by unpaired *t* test. A nested scatterplot (*bottom*) represents quantification (n = 3) of an area of individual droplets from three independent experiments with the BRCA1-BARD1 complex shown in (*C*), with median area determined per dataset. Statistical significance was determined by nested *t* test. *E*, LLPS assays with purified, BRCA1-BARD1, mGFP-p-hCTD, and Cy5-RNA. BRCA1-BARD1 (at 5 μM) was mixed with phosphorylated CTD (2.5 μM) and Cy5-ITS1 RNA (at 15 nM) in the presence of a crowding agent (10% dextran). Representative images from three experiments are depicted as an overlay of differential interference contrast (DIC), GFP, and Cy5. The scale bar represents 10 μm. Quantification of the data can be found in (Fig. S9*B*). CTD, C-terminal domain; RNAPII, RNA polymerase II.

To test this hypothesis, we labeled the BRCA1-BARD1 complex (Fig. 4*A*) *in vitro* with Alexa488. During the assay, we used a 1:20 ratio (labeled:unlabeled) complex, because *in vitro* labeling had a negative effect on condensation propensity (Fig. S9*C*). When the sample was visualized by fluorescence microscopy in the presence of 10% dextran, regularly shaped objects appeared at concentrations as low as 1.25 μM. With increasing concentration (up to 5 μM), these objects grew but their number decreased, suggesting fusion events consistent with liquid-like behavior. To determine which forces drive this condensation, we added two known inhibitors: hexane-1,6-diol (which disrupts hydrophobic interactions) (86) and ATP (which can weaken electrostatic interactions) (87). The presence of hexane-1,6-diol reduced droplet number and led to irregularly shaped objects, indicative of partial aggregation, while ATP (5 mM) substantially reduced droplet size and number (Fig. 4*B*). Therefore, the BRCA1-BARD1 complex undergoes condensation *via* LLPS, which likely relies on both hydrophobic and electrostatic forces.

Next, we investigated whether these BRCA1-BARD1 condensates can incorporate hyperphosphorylated CTD (mCherry-p-hCTD) (as a proxy for RNAPII *in vivo*) and a model ∼1 kb RNA transcript (Cy5-ITS1). Neither pCTD nor RNA in isolation forms condensates (Fig. S15*C*) (70, 88). To this end, we mixed the BRCA1-BARD1 with the said factors either individually or in combination and visualized the objects using fluorescence microscopy. Both pCTD and the RNA transcript were incorporated into condensates formed by the BRCA1-BARD1 complex (Fig. 4, *B*–*D*). Nearly all assessed condensates contained signal for both the BRCA1-BARD1 complex and mCherry-p-hCTD. Similar results were observed when pCTD was substituted with the Cy5-labeled RNA transcript. Finally, we tested whether both pCTD and RNA can coexist in the same droplets. Indeed, we observed mGFP-p-hCTD and Cy5-ITS1 signals within droplets formed by unlabeled BRCA1-BARD1 under the tested conditions, confirming that the complex can simultaneously incorporate pCTD and RNA into the condensates (Fig. 4*E*).

Subsequently, we performed a series of titration experiments in which the concentration of the BRCA1-BARD1 complex and either pCTD or RNA was kept constant, while the other factor was titrated (*i*.*e*., when pCTD was held constant, RNA was titrated, and *vice versa*). Increasing the concentration of pCTD or RNA resulted in a significant increase in the number of condensates (up to 2.5 μM pCTD and up to 15 nM RNA), but only a limited change in their size and no change in the colocalization of the two factors within the condensates. These data suggest that, once the binding sites on the BRCA1-BARD1 complex are saturated, the condensates reach a maximal size under the tested conditions (Fig. S26).

In summary, our results demonstrate that BRCA1-BARD1 can form condensates capable of accommodating both pCTD (as a proxy for RNAPII) and RNA (as a proxy for cellular transcripts).

### The BRCT domains of BRCA1 and BARD1 form condensates able to accommodate pCTD and RNA

Previous work suggested that condensation of the BRCA1-BARD1 complex *in vivo* may involve the BRCT domains in concert with specific transcripts (85), implicating their active role in the process of condensation. We therefore asked whether the isolated BRCT domains could also form droplets containing both RNA and pCTD.

Initially, we confirmed published reports that the BRCT repeats of both BRCA1 and BARD1 bind nucleic acids (DNA and RNA) (89, 90), albeit to a lower extent than the specialized nucleic-acid-binding domain of BRCA1 (residues 421–1079) (91, 92, 93). Importantly, the BRCT domain of BRCA1 bound RNA *via* the same site as the pCTD, whereas the BARD1 BRCT bound RNA using a different site, distinct from the canonical pCTD-binding pocket (Figs. S11, 12, *A*–*C*). This finding is further supported by: (i) competition EMSA, in which pCTD could compete for nucleic-acid binding to BRCA1 BRCT but not to BARD1 BRCT; and (ii) AlphaFold 3-generated models, in which an RNA molecule occupies the canonical phosphoserine-binding site in BRCA1 BRCT but a conserved basic patch near R705 in BARD1 BRCT (Fig. S11*D*; S12D,E; S13; S26J).

Consistent with this, the BRCT domains and RNA formed condensates in a concentration-dependent manner that required intact RNA binding (Fig. S14, *A* and *B*; S15).

**Figure 5:**
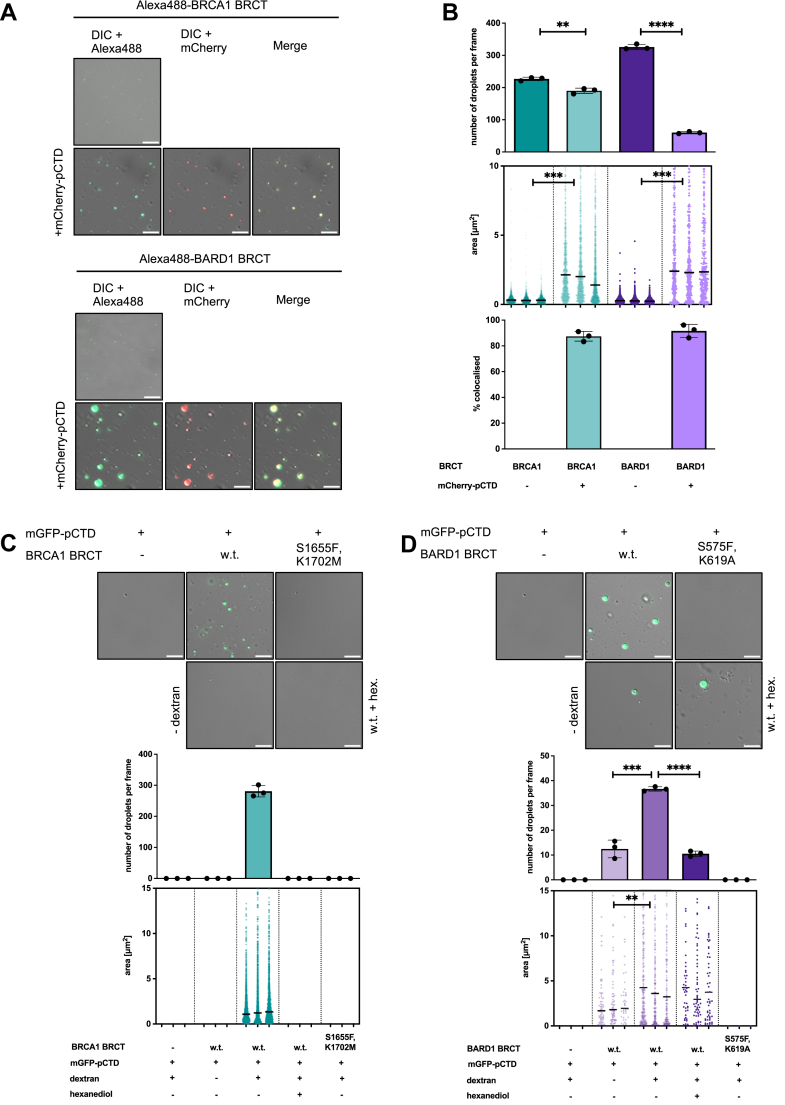
**The BRCT repeats of the BRCA1-BARD1 form liquid-like condensates *in vitro*, which accommodate phosphorylated CTD domain of RNAPII and RNA**. *A*, LLPS assays with mCherry-p-hCTD and Alexa488-labeled BRCA1 BRCT (*top*) and BARD1 BRCT (*bottom*). The BRCA 1 BRCT and BARD1 BRCT (present at the 2.4:1 protein to its CTD binding site ratio), respectively, were mixed with the crowding agent (10% dextran) in absence or presence of phosphorylated CTD (2.5 μM). Representative images from three experiments are depicted as an overlay of differential interference contrast (DIC) with the signal for Alexa488 or mCherry. The scale bar represents 10 μm. *B*, bar chart (*top*) representing quantification (n = 3) of the number of droplets per frame from the LLPS experiments with BRCA1 and BARD1 BRCT shown in (*A*). Statistical significance was determined by unpaired *t* test. A nested scatterplot (*middle*) represents quantification (n = 3) of an area of individual droplets from three independent experiments with BRCA1-BARD1 shown in (*A*), with median area determined per dataset. Statistical significance was determined by nested *t* test. Bar chart (*bottom*) depicts the proportion of droplets containing signals for Alexa488-labeled BRCT (*green*) and mCherry-p-hCTD (*red*). *C*, LLPS assays with purified BRCA1 BRCT and mGFP-p-hCTD. BRCA1 BRCT w.t. and BRCA1 BRCT^S1655F,K1702M^ (present at the 2.4:1 protein to its CTD binding site ratio), respectively, were mixed with phosphorylated CTD (2.5 μM) in the absence or presence of a crowding agent (10% dextran). Representative images (*top*) from three experiments are depicted as an overlay of differential interference contrast (DIC) and GFP. Hexane-1,6-diol (hex; at 10%) was added to inhibit hydrophobic interactions. The scale bar represents 10 μm. Bar chart (*middle*) representing quantification (n = 3) of the number of droplets per frame from the LLPS experiments. Statistical significance was determined by unpaired *t* test. A nested scatterplot (*bottom*) represents quantification (n = 3) of an area of individual droplets from three independent experiments, with median area determined per dataset. Statistical significance was determined by nested *t* test. Representative images from the experiments with three individual BRCT concentrations can be found in Figure S10*A*. *D*, LLPS assays with purified BARD1 BRCT and mGFP-p-hCTD. BARD1 BRCT w.t. and BARD1 BRCT^S575F,K619A^ (present at the 2.4:1 protein to its CTD binding site ratio), respectively, were mixed with phosphorylated CTD (2.5 μM) in the absence or presence of a crowding agent (10% dextran). Representative images (*top*) from three experiments are depicted as an overlay of differential interference contrast (DIC) and GFP. Hexane-1,6-diol (hex; at 10%) was added to inhibit hydrophobic interactions. The scale bar represents 10 μm. Bar chart (*middle*) representing quantification (n = 3) of the number of droplets per frame from the LLPS experiments. Statistical significance was determined by unpaired *t* test. A nested scatterplot (*bottom*) represents quantification (n = 3) of an area of individual droplets from three independent experiments, with median area determined per dataset. Statistical significance was determined by nested *t* test. Representative images from the experiments with three individual BRCT concentrations can be found in Figure S10*C*. CTD, C-terminal domain; LLPS, liquid-liquid phase separation; RNAPII, RNA polymerase II.

Next, we tested whether the pCTD might also enter droplets formed by the BRCT domains of the BRCA1-BARD1 complex. Since isolated CTD phosphorylated on its residues does not undergo phase separation unless its negative charge is shielded by binding factors (70, 84, 88, 94, 95), we used BRCT domain at concentrations sufficient to saturate all binding sites on the pCTD (calculated to be 26 sites in human CTD containing 52 heptapeptide repeats). We therefore mixed mCherry-p-hCTD with *in vitro* fluorescently labeled BRCT repeats (Alexa488, at a 1:20 ratio labeled:unlabeled). Under stoichiometric conditions and in 10% dextran, both BRCT repeats formed droplets with mCherry-p-hCTD that were significantly larger than condensates of BRCT domains in isolation (Fig. 5, *A* and *B*). The colocalization of Alexa488 and mCherry signals implies that both proteins entered the condensates, confirmed by a sedimentation assay in which both pCTD and BRCT repeats were found in the pellet (Fig. S16*B*). Subsequently, we used unlabeled BRCT samples to avoid labeling artefacts (Fig. S14, *C* and *D*).

When LLPS experiments were conducted with BRCA1 BRCT and mGFP-p-hCTD, droplet formation required both proteins, 10% crowding agent, and a direct physical interaction (Fig. 5, *C* and *D*, Fig. S10). These droplets exhibited liquid-like properties, as hexane-1,6-diol disrupted their formation. In contrast, BARD1 BRCT and mGFP-p-hCTD formed droplets even in the absence of a crowding agent, although less efficiently, and still required direct binding. Treatment with hexane-1,6-diol led to only partial disruption, suggesting that some droplets had transitioned to a more gel-like state. This may be explained by higher frequency of fusions of the droplets, which is supported by the presence of higher-order structures in the *in vitro* crosslinking experiments with the BARD1 BRCT, compared to the BRCA1 BRCT (Fig. S16*A*).

These experiments support the view that BARD1 BRCT is more prone to condensation, possibly due to a higher propensity for oligomerization in solution.

Finally, we examined whether both pCTD and RNA can be accommodated together in BRCT-domain droplets. We mixed BRCT domains with pCTD and RNA in the presence of a crowding agent and observed that both domains incorporated pCTD and RNA into the condensates more efficiently in the presence of Mg^2+^. Consistent with the previously presented data, both pCTD and RNA required specific interactions with BRCT domains for efficient incorporation into condensates. However, the BRCA1 BRCT domain utilized a single interface to interact with both pCTD and RNA, whereas the BARD1 BRCT domain used two distinct interfaces for these interactions (Figs. S17, 18). In line with this observation, titration of RNA in LLPS experiments with isolated BRCT domains led to reduced colocalization of the pCTD and RNA signals for BRCA1 BRCT, but not for BARD1 BRCT. Furthermore, titration of either pCTD or RNA caused a decrease in the size of the condensates formed by BRCA1 BRCT, likely due to exceeding the charge-shielding capacity of its single binding site. In contrast, condensates formed by BARD1 BRCT increased in size with higher pCTD or RNA concentrations, consistent with its ability to engage and incorporate additional ligands through separate binding interfaces. (Figs. S27, 28).

In summary, our data show the BRCA1-BARD1 BRCT domains form condensates that can simultaneously incorporate pCTD and RNA. However, the domains differ significantly in the mechanism. Although BRCA1 BRCT uses an overlapping interface for pCTD and RNA, BARD1 BRCT uses separate, discrete binding sites.

### Characterization of disease-associated variants of BRCT domains

**Figure 6:**
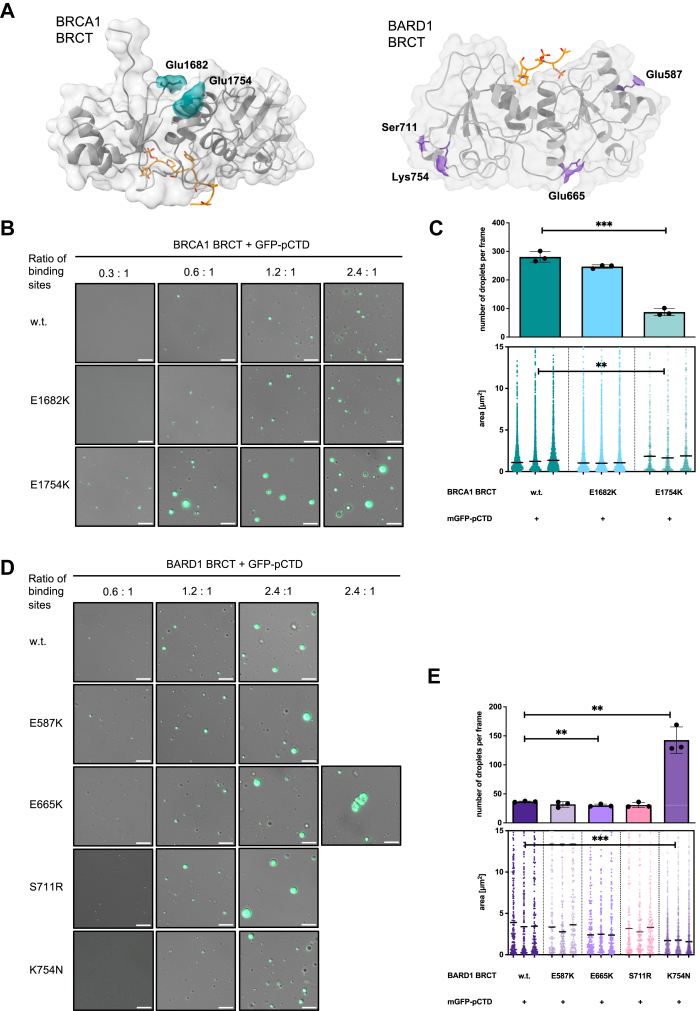
**Characterization of disease-associated variants within the BRCT repeats of the BRCA1-BARD1 complex on their ability to promote condensation *in vitro***. *A*, positions of the investigated substitutions in BRCT domains. The crystal structure of BRCA1 BRCT with the pS5 CTD ligand (*left*) and the AlphaFold 3-generated model of the BARD1 BRCT with the pS5 CTD ligand (*right*) were used. Investigated substitutions are not located near the phospho-peptide binding sites and therefore should not interfere with the binding. *B* LLPS assays with purified BRCA1 BRCT and mGFP-p-hCTD. BRCA1 BRCT w.t., BRCA1 BRCT^E1682K^, and BRCA1 BRCT^E1754K^, respectively, present at the indicated protein to its CTD binding site ratios, were mixed with phosphorylated CTD (2.5 μM) in the presence of a crowding agent (10% dextran). Representative images from three experiments are depicted as an overlay of differential interference contrast (DIC) and GFP. The scale bar represents 10 μm. *C*, bar chart (*top*) representing quantification (n = 3) of the number of droplets per frame from the LLPS experiments with BRCA1 BRCT w.t., BRCA1 BRCT^E1682K^, and BRCA1 BRCT^E1754K^, respectively, present at the 2.4:1 protein to its CTD binding site ratio, and mGFP-p-hCTD, shown in (*B*), using the *green* fluorescent signal. Statistical significance was determined by unpaired *t* test. A nested scatterplot (*bottom*) representing quantification (n = 3) of an area of individual droplets from three independent experiments with BRCA1 BRCT, mGFP-p-hCTD, shown in (*B*), with median area determined per dataset. Statistical significance was determined by nested *t* test. Quantification of the experiments with four individual BRCT concentrations can be found in Figure S22*A*. *D*, LLPS assays with purified BARD1 BRCT and mGFP-p-hCTD. BARD1 BRCT w.t., BARD1 BRCT^E587K^, BARD1 BRCT^E665K^, BARD1 BRCT^S711R^, and BARD1 BRCT^K754N^, respectively, present at the indicated protein to CTD binding site ratio, were mixed with phosphorylated CTD (2.5 μM) in the presence of a crowding agent (10% dextran). Representative images from three experiments are depicted as an overlay of differential interference contrast (DIC) and GFP. The scale bar represents 10 μm. *E*, bar chart (*top*) representing quantification (n = 3) of the number of droplets per frame from the LLPS experiments with BARD1 BRCT w.t., BARD1 BRCT^E587K^, BARD1 BRCT^E665K^, BARD1 BRCT^S711R^, and BARD1 BRCT^K754N^, respectively, present at the 2.4:1 protein to its CTD binding site ratio, and mGFP-p-hCTD, shown in (*D*), using the *green* fluorescent signal. The analysis and visualization were performed as in (*C*). Quantification of the experiments with three BRCT concentrations can be found in Figure S22*B*. CTD, C-terminal domain; LLPS, liquid-liquid phase separation; pS5-CTD, phosphorylated on serine 5-CTD.

The ability of the BRCT domains to form condensates provided a direct readout to investigate uncharacterized disease-associated variants of BRCA1 and BARD1 within their BRCT repeats (96). We focused on several conserved residues lying outside the phospho-serine-binding region (Fig. 6*A*, Fig. S19).

We purified and analyzed the following variants: BRCA1 BRCT^E1682K^, BRCA1 BRCT^E1754K^, and BARD1 BRCT^E587K^, BARD1 BRCT^E665K^, BARD1 BRCT^S711R^, and BARD1 BRCT^K754N^ (Fig. S1, *A* and *B*). These variants showed significantly lower thermal stability but retained wild-type affinity toward pCTD (Figs. S20, 21).

When we tested the ability of these variants to form condensates in the presence of pCTD, BRCA1 BRCT^E1754K^ exhibited a significantly higher propensity for condensation (droplets visible already at 20 μM), whereas BRCA1 BRCT^E1682K^ did not show this effect (Fig. 6*B*; Figs. S22*A*; S23*A*). Among the BARD1 variants, BRCT^K754N^ showed defective droplet fusion, producing numerous smaller droplets (Fig. 6*C*; Fig. S22*B*). Even though variant BARD1 BRCT^E665K^ maintained wild-type level of condensates, it exhibited higher propensity to aggregate as evidenced by the appearance of irregularly shaped objects. Moreover, when we labeled this variant *in vitro*, only aggregates were observed in the presence of the crowding agent, unlike the w.t. variant (Fig. 6*C*, Figs. S22*B*; S22*B*).

Fittingly, the behavior of the abovementioned, disease-associated, variants found within both BRCT domains of BRCA1 and BARD1, respectively, differed also in absence of the pCTD. *In vitro* labeled BRCA1 BRCT^E1754K^ formed more nucleation centers, while BARD1 BRCT^K754N^ formed fewer nucleation centers than their respective w.t. variants, indicating a change in transient intermolecular interactions, which may subsequently drive the fusion of the pCTD droplets (Fig. S23*D*). This was reproduced using an alternative approach–*in vitro* sedimentation assay (Fig. S24). These results further strengthen our observation on the effect that the investigated, disease-associated substitution have on condensation properties of the domains.

Finally, as the BARD1 BRCT^S711R^ substitution introduces a positive charge in the proximity of the RNA binding site within the BARD1 BRCT domain, we hypothesized that this substitution could affect the interaction of BARD1 BRCT with RNA. Indeed, this variant exhibited small, albeit significant, increase in affinity for RNA compared to the w.t. and increased propensity for the fusion of the condensates with RNA (Fig. S25).

Overall, we tested several previously uncharacterized disease-associated variants of BRCA1 and BARD1, some of which altered the condensate-forming properties of the BRCT domains, suggesting that defective condensation might contribute to tumorigenesis.

In summary, we provide a comprehensive mechanistic characterization of how the BRCA1-BARD1 complex interacts with RNAPII, which may link transcription and DNA repair. Our data also offer new insight into a potential functional role for this interaction in heterotypic condensates containing the BRCA1-BARD1 complex, RNAPII, and nucleic acids.

## Discussion

Understanding the mechanisms underlying crosstalk between essential cellular processes that compete for DNA–replication, recombination, repair, and transcription–is critical for understanding how these processes are orchestrated in a way that prevents collisions and possible genome instability (1, 2, 3, 4). Moreover, emerging concepts in mesoscale organization and compartmentalization of these processes *via* condensation in the nucleus suggest that the current state of the art is far from complete (66, 80, 97).

In this study, we focused on a detailed, mechanistic characterization of the interaction between the BRCA1-BARD1 complex and RNAPII, with the aim of improving our understanding of the crosstalk between transcription and DNA repair. To this end, we performed a comparative analysis of the BRCT domains found in both BRCA1 and BARD1. Furthermore, we solved the 3D structure of a complex between the BRCA1 BRCT domain and a phosphopeptide containing two heptads from the CTD of the catalytic subunit of RNAPII pS5-CTD. Finally, we explored the functional consequences of this interaction, uncovering a possible role of the BRACA1-BARD1 complex in the organization of transcription and/or repair factories *via* condensation.

The association of the BRCA1-BARD1 complex with ongoing transcription has been known for decades (29, 30). The initial proposition that the complex might, through its E3 ubiquitin ligase activity (*via* the RING domains) (73, 98, 99), mediate ubiquitination of RNAPII and its subsequent removal from chromatin upon DNA damage has not been corroborated; as other E3 ubiquitin ligases were shown to mediate this process (100, 101, 102). This leaves open the question of the functional significance of the interaction.

Presently, we do not know the precise context in which the BRCA1-BARD1 complex associates with RNAPII *in vivo*. Does the complex bind RNAPII that has been paused or stalled due to encountering a lesion on the template, or does it associate with RNAPII that has encountered a broken chromosome undergoing repair by recombination? Alternatively, does the interaction reflect the role of RNAPII in *de novo* RNA generation specifically at sites of DSBs, where the BRCA1-BARD1 complex is also present? There is also the possibility that the interaction might be S-phase specific, reflecting the possibility that sites of transcription-replication conflicts might be the place wherein the complex associates with RNAPII. In addition, the complex might play a role in the release of RNAPII stalled at promoter-proximal regions, as observed in instances in which MYCN-dependent transcription activation leads to accumulation of RNAPII molecules (34). Furthermore, it remains unclear whether this association is functionally linked to the E3 ubiquitin ligase activity of BRCA1-BARD1 (103, 104), whereby associating with RNAPII would bring the ubiquitin machinery to specific genomic loci. These are only a few unanswered questions.

We sought to help address these questions by providing a thorough, mechanistic characterization of the interaction. Determining that the preferred substrate is pS5-CTD helps narrow down the context in which the BRCA1-BARD1 complex may associate with RNAPII. This modification is associated with the early stages of the transcription cycle (105, 106, 107), and there is supporting *in vivo* evidence for the complex to be associated with pS5-CTD (30).

Moreover, our comparative analysis of the BRCT domains within the BRCA1-BARD1 complex revealed that they substantially differ in their mode of binding to the pCTD of RNAPII. The BRCA1 BRCT stably associates with pCTD, whereas the BARD1 BRCT displays a more dynamic binding, likely because of the differences in the hydrophobic pocket in BARD1 BRCT. These results may also offer an alternative explanation for a previous observation (74), suggesting that the BARD1 BRCT binds a particular phosphoprotein substrate only at lower temperature, which was linked to thermal instability of BARD1 BRCT domain. Our kinetic approach suggests that the perceived, limited to no binding by BARD1 BRCT is caused rather by its more dynamic, less stable association, which could be stabilized at lower temperatures, than by BARD1 BRCT thermal stability (Fig. S20*B*).

Furthermore, if the increased binding dynamics observed for BARD1 BRCT extend to its other phosphorylated ligands, it could explain the difficulties encountered in cocrystallizing the domain with its putative binding partners (42). In addition, the lack of structural data for the BARD1 BRCT domain bound to its cognate ligand might point to the possibility that phosphoproteins are not the best substrates for the BARD1 BRCT domain. Chemically diverse ligands, such as nucleosomes or (poly)-ADP-ribose, have been suggested as potential binding partners (46, 108, 109, 110, 111).

**Figure 7:**
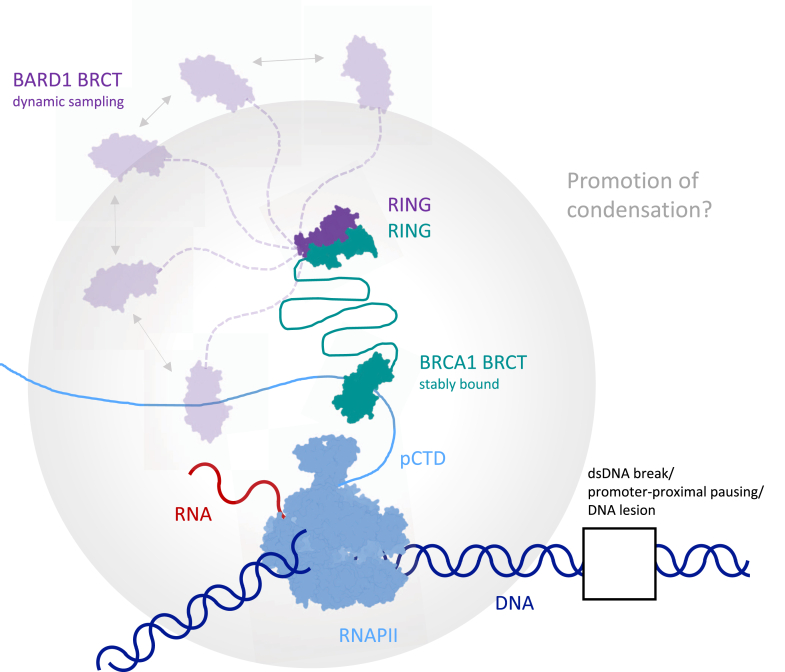
**Schematic model of the interaction between RNAPII and the BRCA1-BARD1 complex**.The BRCA1-BARD1 complex may associate with the transcriptionally engaged RNAPII phosphorylated on Ser5 of the CTD (pCTD) *via* the BRCT domains of BRCA1 and BARD1 *e*.*g*., during unscheduled pausing of RNAPII for instance at a double-stranded DNA break. The more stable interaction between the BRCA1 BRCT domain and the pCTD might serve to anchor the complex to RNAPII, whilst the BARD1 BRCT domain may engage more dynamically, thereby enabling the complex to sample the vicinity of the paused RNAPII. These interactions might contribute to the formation of condensates containing the BRCA1-BARD1 complex alongside the transcriptionally engaged RNAPII, although this remains to be confirmed *in vivo*. RNAPII, RNA polymerase II; CTD, C-terminal domain

The difference in the binding between the BRCT domains could be summarized in the following model (Fig. 7). The BRCA1-BARD1 complex may use the BRCA1 BRCT for stable association with transcribing RNAPII, which may or may not stall due to presence of damage on the template, whereas BARD1 BRCT could dynamically interact with other factors, especially given the flexibility and length (∼700 Å) of the CTD (112, 113, 114), thereby scouting the surroundings of the RNAPII for the presence of specific binding partners (let those be phosphoproteins, chromatin, or (poly)-ADP-ribose chains linked to proteins or DNA), and subsequently possibly promoting damage-dependent ubiquitination *via* the RING domains found on the N termini. Importantly, to determine whether these interactions play a role in facilitating condensation of the BRCA1-BARD1 complex and transcription-engaged RNAPII *in vivo* (as would our LLPS experiments suggest), further validation in cells is required. Such experiments should avoid overexpressing only one subunit of the complex, which exposes a nuclear-export signal otherwise buried within the RING-mediated BRCA1-BARD1 dimer interface and leads to pan-cellular localization of the overexpressed protein ((115); and our unpublished observations).

The presented crystal structure of the complex between the BRCA1 BRCT domain and a diheptad of CTD pS5-(CTD)_2_ is the first structure of a BRCT domain bound to the pCTD, expanding the repertoire of domains that recognize a specific pCTD modification. The canonical two-anchor mode of recognition, wherein the BRCT domain specifically recognizes the phosphoserine at position 5 and the tyrosine at position 1 of the following repeat in the CTD, aligns well with existing crystal structures of BRCA1 BRCT with other phosphoprotein ligands (38, 40, 41, 77, 78). The phosphorylated CTD diheptad is a relatively poor binding substrate (by at least an order of magnitude) compared with other known BRCA1 BRCT ligands (38, 40, 41, 78, 116). However, given the repetitive nature of the CTD, this lower affinity is likely compensated by avidity effects stemming from the 52-repeat CTD on RNAPII, as evidenced by the BLI measurements with a 26-repeat CTD. A similar effect has been proposed for other domains recognizing the CTD (117).

When exploring the functional consequences of the interaction between the BRCA1-BARD1 complex and RNAPII, we found that the complex forms condensates *in vitro*, which can simultaneously accommodate pCTD (a likely proxy for RNAPII *in vivo*) and an RNA transcript. Intriguingly, the isolated BRCA1 and BARD1 BRCT domains also formed condensates that incorporated pCTD and RNA. As the BRCA1-BARD1 complex has been implicated in specific condensates *in vivo*, (85) our data suggest that the complex may not only be recruited to such condensates but could also actively participate in their formation. Moreover, we and others have recently shown that other DNA repair factors, such as the trimeric SOSS1 complex (70) and 53BP1 ^24^ promote condensation *in vivo* at DSB sites, which also contain RNAPII. Thus, it is possible to speculate that the BRCA1-BARD1 complex might promote the organization of condensates containing RNAPII *in vivo*. To strengthen our *in vitro* observations, we characterized several disease-associated variants of the BRCT repeats. We identified variants that showed defects in their ability to form condensates, possibly linking defective condensation to pathology. Further experiments addressing the effect of the mutations *in vivo* will be required to corroborate these observations.

In conclusion, our study provides mechanistic insight into the interaction between the BRCA1-BARD1 complex and RNAPII and proposes potential functional roles. We anticipate that our results will enable better interpretation of existing *in vivo* data and may guide future studies aimed at uncovering the biological relevance of the BRCA1-BARD1-RNAPII association.

## Experimental procedures

### Plasmid construction

The plasmids were constructed using ligation-independent cloning (LIC), a method, which enables the insertion of DNA fragments into plasmids without the requirement for DNA ligase, and enables the combination of multiple inserts into a single plasmid (118).

To express BRCA1 BRCT domains (amino acids 1646–1859) in *Escherichia coli*, fragment of DNA, containing the coding sequence was amplified by PCR and cloned into 2BT (pET His6 TEV LIC cloning vector; addgene #29666) using LIC. Point mutations resulting in the following amino acid substitutions: S1655F, E1682K, R1699W, R1699L, K1702M, E1754K, S1755Y, M1775K, and M1775H, were introduced by site-directed mutagenesis.

To express BARD1 BRCT domains (amino acids 554–777) in *E*. *coli*, fragment of DNA, containing the coding sequence was amplified by PCR and cloned into 2BT vector. Point mutations resulting in the following amino acid substitutions: S575F, E587K, K619A, E665K, H686M, R705A, S711R, and K754N, were introduced by site-directed mutagenesis.

To generate plasmids for expression of Avi-tagged BRCA1 BRCT domains (amino acids 1646–1859) in *E*. *coli*, fragment of DNA, containing the coding sequences of BRCA1 BRCT w.t., S1655F, K1702A, E1754K, and M1775H, respectively, were amplified by PCR and cloned into H6-msfGFP plasmid (pET Biotin His6 GFP LIC cloning vector; addgene #29725) to add Avi-tag to the sequence. To generate plasmids for expression of Avi-tagged BARD1 BRCT domains (amino acids 554–777), the fragments of DNA, containing the coding sequences of BARD1 BRCT w.t., S575F, K619A, E665K, H686M, R705A, and K754N, respectively, were amplified by PCR and cloned into H6-msfGFP plasmid (pET Biotin His6 GFP LIC cloning vector; addgene #29725) to add Avi-tag to the sequence. Subsequently, the Avi-tagged fragments were subcloned into 2AT plasmid (pET LIC cloning vector; addgene #29665).

To generate plasmid enabling the expression of biotin-ligase BirA, the coding sequence of BirA was amplified by PCR and cloned into plasmid 2CT-10 (pET His10 MBP Asn10 TEV LIC cloning vector; addgene #55209).

To express the DNA binding region of BRCA1 (amino acids 421–1079), the fragment of DNA, containing the coding sequence was amplified by PCR and cloned into 438C (pFastBac His6 MBP Asn10 TEV cloning vector with BioBrick PolyPromoter LIC Subcloning, addgene #55220).

To generate a vector for coexpression of the full-length human BRCA1-BARD1 complex in insect cells, fragment of DNA, containing FLAG-tagged BRCA1 was first cloned into 2BcT plasmid (pET His6 LIC cloning vector; addgene #37236) to add C-terminal (His)_6_ tag to the construct. Subsequently, FLAG-BRCA1-His was subcloned into plasmid 438A (pFastBac cloning vector with BioBrick PolyPromoter LIC Subcloning, addgene #55218). The DNA fragment containing the ORF for BARD1 was cloned into 438B plasmid (pFastBac His6 TEV cloning vector with BioBrick PolyPromoter LIC Subcloning; addgene #55219). Subsequently, 438A-FLAG-BRCA1-(His)_6_ and 438B-BARD1 plasmids were combined using BioBrick Polypromoter LIC subcloning into a single construct. Point mutations resulting in the amino acid substitutions S1655F,K1702M in BRCA1 and S575F,K619A in BARD1 were introduced into BRCA1 and BARD1 individually. Subsequently, the plasmids were combined using BioBrick Polypromoter LIC subcloning into a single construct, which resulted in following combinations: BRCA1^2M^-BARD1^w.t.^ (BRCA1^S1655F,K1702M^-BARD1), BRCA1^w.t.^-BARD1^2M^ (BRCA1-BARD1^S575F,K619A^), and BRCA1^2M^-BARD1^2M^ (BRCA1^S1655F,K1702M^- BARD1^S575F,K619A^).

The plasmids enabling expression of the kinase module of TFIIH complex (the CDK7 complex) in insect cells, and the plasmid enabling the expression of the catalytic domain of cABL were described in (70). Plasmids 2BcT-msfGFP-hCTD and 2BcT-mCherry-hCTD were described in (88). The plasmid enabling the expression of the catalytic domain of DYRK1A was a gift from Nicola Burgess-Brown (Addgene plasmid #38913). Plasmid pGEX4T1-(CTD)_26_-(His)_7_ (provided by Olga Jasnovidova) was used to express and purify GST-(CTD)_26_-(His)_7_. Plasmid pDRIVE-ITS1 used to generate ITS1 RNA by *in vitro* transcription was kindly provided by Stepanka Vanacova.

Plasmids 2AT, 2BT, 2CT-10, 2GT, 2Bc-T, H6-msfGFP, 438A, and 438B were purchased directly from QB3 Macrolab (UC Berkeley). Oligonucleotides used in this study are listed in Table S2, list of recombinant plasmids generated in this work is provided in Table S3. The sequence integrity of all constructs was verified by sequencing.

### Synthetic RNA/DNA substrates

Oligonucleotides for preparing synthetic fluorescently labeled (Cy3) RNA/DNA substrates were purchased from Sigma-Aldrich (HPLC purified). Substrates were prepared by mixing 3 pmol of labeled oligonucleotides with a 3-fold excess of the unlabeled oligonucleotides in the annealing buffer (25 mM Tris–HCl, pH 7.5; 100 mM NaCl; and 3 mM MgCl_2_), followed by initial denaturation at 75 °C for 5 min. Substrates were then purified from a native PAGE gel.

### Protein expression in *E*. *coli*

His-BARD1^554-777^, Avi-BARD1^554-777^, and MBP-BirA were expressed in *E*. *coli* RIPL strain. BRCA1^1646-1859^ and Avi-BRCA1^1646-1859^ were expressed in *E*. *coli* Rosetta 1 strain. The expression of the proteins was induced at *A*_600_ = 0.6 by addition of IPTG (final concentration 1 mM) and the cultures were incubated overnight at 16 °C.

GST-(CTD)_26_, mCherry-hCTD, and mGFP-hCTD were expressed in *E*. *coli* BL21-AI strain grown in Terrific Broth (TB) media. The expression of the proteins was induced at *A*_600_ = 0.6 by addition of IPTG (final concentration 0.1 mM) and L-arabinose (final concentration 0.02%) and the cultures were incubated overnight at 30 °C.

### Protein expression in insect cells

To generate viruses enabling the production of proteins in insect cells, the coding sequences and the necessary regulatory sequences of the aforementioned constructs were transposed into bacmids using the *E*. *coli* strain DH10bac. Viral particles were obtained by transfection of the corresponding bacmids into the *Sf*9 cells using the FuGENE6-HD Transfection Reagent and further amplification. Proteins were expressed in 300 to 600 ml of Hi5 cells (infected at 1.2 × 10^6^ cells/ml) with the corresponding P1 virus at multiplicity of infection > 1. Cells were harvested 48 h postinfection, washed with 1xPBS, and stored at -80 °C.

### Protein purification

#### Purification of BRCA11646-1859 and BARD1554-777

Eight grams of *E*. *coli* pellet was resuspended in 40 ml of ice-cold lysis buffer (50 mM Tris–HCl, pH 7.9; 500 mM NaCl; 1 mM DTT; and 10 mM imidazole) containing one tablet of cOmplete EDTA-free Protease Inhibitors. Cells were opened up by sonication, the lysate was cleared by centrifugation and incubated for 1 h with 5 ml of Ni-NTA beads (Qiagen), equilibrated with the lysis buffer. The proteins were eluted with an elution buffer containing 25 mM Tris-HCl, pH 7.9; 300 mM NaCl; 1 mM DTT; and 400 mM imidazole. Elution fractions were dialyzed overnight against dialysis buffer (25 mM Tris–HCl, pH 7.9; 300 mM NaCl; and 1 mM DTT). The (His)_6_-tag was cleaved off by the tobacco etch virus (TEV) protease during the dialysis step. The sample was then purified from the TEV protease using Ni-NTA beads, concentrated, and loaded onto Superdex S-75 column equilibrated in a buffer containing 25 mM Tris–HCl, pH 7.9; 300 mM NaCl; and 1 mM DTT. The samples for phase separation assays were purified using Superdex S-75 column equilibrated in a buffer containing 25 mM Hepes, pH 7.5; 220 mM NaCl; and 1 mM DTT. The fractions containing purified protein were concentrated, snap-frozen in liquid nitrogen, and stored at −80 °C.

#### Purification of BirA, Avi-BRCA11646-1859 and Avi-BARD1554-777

Eight grams of *E*. *coli* pellet was resuspended in 40 ml of ice-cold lysis buffer (50 mM Tris–HCl, pH 7.9; 500 mM NaCl; 1 mM DTT; 10 mM imidazole) containing one tablet of cOmplete, EDTA-free Protease Inhibitors. Cells were opened up by sonication, the lysate was cleared by centrifugation and incubated for 1 h with 5 ml of Ni-NTA beads (Qiagen), equilibrated with the lysis buffer. The proteins were eluted with an elution buffer containing 25 mM Tris–HCl, pH 7.9; 300 mM NaCl; 1 mM DTT, and 400 mM imidazole. Elution fractions were concentrated, and the proteins were further purified using gel filtration column equilibrated in buffer containing 50 mM Tris–HCl, pH 7.9; 300 mM NaCl, and 1 mM DTT. BirA was purified using Superdex S-200 column, Avi-BRCA1^1646-1859^ and Avi-BARD1^554-777^ were purified using Superdex S-75 column. The fractions containing purified protein were concentrated, snap-frozen in liquid nitrogen, and stored at −80 °C.

#### *In vitro* biotinylation and purification of biotinylated BRCA1^1646-1859^ and BARD1^554-777^

For preparative purposes, 1 mg of Avi-BRCA1^1646-1859^ and Avi-BARD1^554-777^, respectively, were biotinylated by 800 μg of the MBP-BirA in the presence of 3 mM ATP, 3 mM MgCl_2_, and 200 μM biotin for 2 h at 23 °C. Reactions were loaded directly onto Superdex S-200 equilibrated with 25 mM Tris–HCl, pH 7.9, 250 mM NaCl, and 1 mM DTT to purify the biotinylated proteins from BirA and ATP. Fractions containing biotinylated BRCA1^1646-1859^ and BARD1^554-777^ were pooled, concentrated, snap-frozen in liquid nitrogen, and stored at −80 °C.

### Purification of BRCA1^421-1079^ from insect cells

Pellets from 300 ml of the Hi5 culture were resuspended in ice-cold lysis buffer (50 mM Tris–HCl, pH 7.9; 500 mM NaCl; 10% (v/v) glycerol; 1 mM DTT; 0.4% (v/v) Triton-X; and 10 mM imidazole) containing protease inhibitors (0.66 μg/ml pepstatin, 5 μg/ml benzamidine, 4.75 μg/ml leupeptin, and 2 μg/ml aprotinin) and 25 U benzonase per ml of lysate. The cleared lysate was incubated for 1 h with 3 ml of Ni-NTA beads (Qiagen), equilibrated with 50 mM Tris–HCl, pH 7.9; 500 mM NaCl; 10 mM imidazole; and 1 mM DTT. The proteins were eluted with elution buffer containing 25 mM Tris–HCl, pH 7.9; 300 mM NaCl; 1 mM DTT, and 400 mM imidazole. Elution fractions were concentrated, and the proteins were further purified using Superdex S-200 column equilibrated in buffer containing 50 mM Tris–HCl, pH 7.9; 300 mM NaCl, and 1 mM DTT. Fractions containing purified protein were concentrated, and glycerol was added to a final concentration of 10% before they were snap-frozen in liquid nitrogen, and stored at -80 °C.

### Purification of the BRCA1-BARD1 complex from insect cells

Pellets from 600 ml of the Hi5 culture were resuspended in ice-cold lysis buffer (50 mM Tris–HCl, pH 7.5; 500 mM KCl; 10% (v/v) glycerol; 5 mM MgCl_2_; 2 mM ATP; 1 mM DTT; 0.5% (v/v) NP-40; and 10 mM imidazole) containing protease inhibitors (0.66 μg/ml pepstatin, 5 μg/ml benzamidine, 4.75 μg/ml leupeptin, and 2 μg/ml aprotinin) and 25 U benzonase per ml of lysate. The cleared lysate was incubated for 1 h with 3 ml of Ni-NTA beads (Qiagen), equilibrated with the lysis buffer. The beads were then washed extensively with the wash buffer (25 mM Tris–HCl, pH 7.5; 500 mM KCl; 10% (v/v) glycerol; 5 mM MgCl_2_; 2 mM ATP; 1 mM DTT; 10 mM imidazole; and protease inhibitors) and subsequently, the proteins were eluted with the elution buffer (25 mM Tris–HCl, pH 7.5; 300 mM KCl; 10% (v/v) glycerol; 5 mM MgCl_2_; 2 mM ATP; 1 mM DTT; 400 mM imidazole; and protease inhibitors). Elution fractions were concentrated, His-tag was cleaved off using TEV protease and the proteins were further purified using Superose 6 column equilibrated in the gel filtration buffer containing 50 mM Tris–HCl, pH 7.5; 300 mM KCl; and 1 mM DTT. For the biochemical purposes, fractions containing purified protein were concentrated, supplemented with glycerol to a final concentration of 10%, snap-frozen in liquid nitrogen, and stored at -80 °C.

### Purification of ABL1^cat^ and DYRK1A^cat^

Five grams of *E*. *coli* BL21 RIPL cells expressing the catalytic domain of ABL1 and DYRK1A, respectively, were resuspended in ice-cold lysis buffer (50 mM Tris–HCl, pH 8; 0.5 M NaCl; 10 mM imidazole; and 1 mM DTT), containing protease inhibitors (0.66 μg/ml pepstatin, 5 μg/ml benzamidine, 4.75 μg/ml leupeptin, and 2 μg/ml aprotinin) at +4 °C. Cells were opened up by sonication. The cleared lysate was passed through 2 ml of Ni-NTA beads (Qiagen), equilibrated with buffer (50 mM Tris–HCl, pH 8; 500 mM NaCl; 10 mM imidazole; and 1 mM DTT). Proteins were eluted with an elution buffer (50 mM Tris–HCl, pH 8; 500 mM NaCl; 1 mM DTT, and 400 mM imidazole). The elution fractions containing the proteins of interest were pooled, concentrated, and further fractionated on Superdex S-75 column with SEC buffer (25 mM Tris–Cl pH 7.5; 200 mM NaCl, and 1 mM DTT). Fractions containing pure proteins of interest were concentrated, glycerol was added to a final concentration of 10% before they were snap-frozen in liquid nitrogen, and stored at −80 °C.

Purified T7 RNAP was kindly provided by Martin Mátl (CEITIC, MU). The purification of the CTD peptides and their subsequent *in vitro* phosphorylation was described earlier (70, 88).

### Pull-down assays from HEK293T cell lysates

HEK293 human cell lines were cultivated in Dulbecco's modified Eagle's medium (Sigma-Aldrich) supplemented with 10% (v/v) fetal bovine serum (Tet-free approved, Sigma-Aldrich), 100 U/ml penicillin, and 100 μg/ml streptomycin (Gibco). Cells were grown at 37 °C in humidified atmosphere in 5% CO2. Typically, one HEK293 pellet from one 10 cm dish was used per condition. The pellet was resuspended in 1 ml of nuclear/cytoplasmic extraction buffer (NETN) buffer (50 mM Tris–HCl, pH 8; 150 mM NaCl; 1 mM EDTA; 0.5% NP-40; and bovine serum albumin (final concentration 0.1 mg/ml)) containing benzonase nuclease and cOmplete, EDTA-free Protease Inhibitors. The sample was incubated for 30 min at 4 °C and sonicated briefly. The lysate was cleared by centrifugation and the supernatant was incubated with 5 μg of FLAG-tagged BRCA1-BARD1 for 30 min at 4 °C. Subsequently, the sample was added to 25 μl of ANTI-FLAG M2 Affinity Gel (Sigma-Aldrich), equilibrated in NETN buffer, and incubated for 30 min at 4 °C. The beads were washed four times with 4 column volume of NETN buffer, and the bound proteins were eluted with 50 μl of the NETN buffer containing 3xFLAG peptide (Sigma-Aldrich) (final concentration 0.25 mg/ml). The input and elute were analyzed by western blotting.

### *In vitro* pull-down assay

Purified GST-(CTD)_26_, GST-pY1-(CTD)_26_, GST-pS2pS5-(CTD)_26_, and GST-pS5pS7-(CTD)_26_ (5 μg each), respectively, were incubated with BRCA1-BARD1, BRCA1 BRCT, and BARD1 BRCT (5 μg each) in 30 μl of buffer T200 (25 mM Tris–HCl pH 7.5; 200 mM NaCl; 10% glycerol; 1 mM DTT; 0.5 mM EDTA; and 0.01% NP-40) for 30 min at 4 °C in the presence of GSH-beads. After washing the beads twice with 100 μl of buffer T200, the bound proteins were eluted with 30 μl of 4xSDS loading dye. The input, supernatant, and eluate, 7 μl each, were analyzed on SDS-PAGE gel.

### Electrophoretic mobility shift assay

Increasing concentrations of tested proteins (25, 50, 100, and 200 nM of BRCA1^421-1079^ and 3.125, 6.25, 12.5, and 25 μM of BRCA1^1646-1859^ and BARD1^554-777^, respectively) were incubated with fluorescently labeled nucleic acid substrates (at a final concentration of 10 nM) in EMSA buffer (25 mM Tris–HCl, pH 7.5; 1 mM DTT; 5 mM MgCl_2_; and 100 mM NaCl) for 20 min on ice. Loading buffer (60% glycerol in 0.001% Orange-G) was added to the reaction mixtures and the samples were loaded onto a 7.5% (w/v) polyacrylamide native gel in 0.5 x TBE buffer and run at 75 V for 1 h at +4 °C. The different nucleic acid species were visualized using FLA-9000 Starion scanner and quantified in the MultiGauge software (Fujifilm). To calculate the relative amount of bound nucleic acid substrate, the background signal from the control sample (without protein) was subtracted using the band intensity—background option. Nucleic acid-binding affinity graphs were generated with Prism-GraphPad 9.

#### Competition EMSA assays in the presence of CTD polypeptide

To assess the effect of phosphorylated CTD on the biding of BRCA1^1646-1859^ or BARD1^554-777^ to nucleic acid substrate, BRCA1^1646-1859^ and BARD1^554-777^ (12.5 μM), respectively, were incubated with the nucleic acid substrate (10 nM) for 15 min on ice. Subsequently, increasing concentrations (0.1, 0.3, and 0.9 mM) of the GST-pS5pS7-(CTD)_26_ were added, and the reaction mixtures were further incubated for 15 min on ice. Reactions were next processed as described above. The statistical significance was determined by unpaired *t* test analysis.

### *In vitro* cross-linking experiments

To determine the oligomeric state of BRCA1^1646-1859^ and BARD1^554-777^, respectively, the proteins were diluted to 0.5 mg/ml and 2.5 mg/ml, respectively, in buffer H_150_ (25 mM Hepes pH7.5; 150 mM NaCl; and 1 mM DTT). To the diluted proteins, glutaraldehyde (0.05% final concentration) was added, and the mixture was incubated for 30 min at 4 °C. Subsequently, the reactions were stopped by the addition of 1 μl of 1M Tris–HCl. The reactions were analyzed by SDS-PAGE.

### Differential scanning fluorimetry

The thermal stability of BRCA1^1646-1859^ and BARD1^554-777^ w.t. and their substitution variants was measured using the Prometheus NT.38 instrument (NanoTemper Technologies). NanoDSF grade capillaries were filled with protein samples at 1 mg/ml in protein dilution buffer (25 mM Tris–HCl, pH 7.9; 250 mM NaCl; and 1 mM DTT). Protein unfolding was detected at a temperature range of 20 to 95 °C with a 1 °C/min heating rate and 90% excitation power. Onset of protein unfolding (T_on_) and protein melting temperature (T_m_) were determined from the ratio of tryptophan emission at 330 and 350 nm. The temperature of protein aggregation onset (T_agg_) was determined by light scattering.

### Fluorescence anisotropy binding assays

The binding of BRCA1^1646-1859^ and BARD1^554-777^ to CTD peptides was characterized using FluoroLog-3 spectrofluorometer (Horiba Jobin-Yvon Edison) equipped with a thermostatic cell holder with a Neslab RTE7 water bath (Thermo Fisher Scientific). The 5,6-FAM-labeled CTD peptides, purchased from Caslo ApS, were diluted to 25 nM in fluorescence anisotropy buffer (25 mM Tris–HCl, pH 7.9; 150 mM NaCl; and 1 mM DTT). BRCA1^1646-1859^ and BARD1^554-777^ samples were titrated against a constant concentration of fluorescently labeled peptides at 25 °C. Samples were excited with vertically polarized light at 467 nm, and both vertical and horizontal emissions were recorded at 516 nm. The experiments were performed in technical triplicates. Anisotropy data were plotted as a function of protein concentration and fitted to a single-site saturation with nonspecific binding model using XMGrace. The graphs were generated using GnuPlot.

### Biolayer interferometry

To characterize the kinetics of binding of phosphorylated CTD to BRCA1^1646-1859^ and BARD1^554-777^, BLI experiments were performed using Octet RED96e (ForteBio) at 25 °C, with shaking at 2200 rpm. The streptavidin biosensors (ForteBio) were prehydrated in BLI buffer (25 mM Tris–HCl, pH 7.9; 150 mM NaCl; 1 mM DTT; and 0.05% (v/v) Tween 20) for 10 min. Biotinylated BRCA1^1646-1859^ and BARD1^554-777^, respectively, were immobilized on the biosensor tip at a final concentration of 10 μg/ml. The association of nonphosphorylated or pS5pS7 phosphorylated GST-(CTD)_26_ to the immobilized proteins was measured at five different concentrations of the analyte (1.4, 4.3, 13, 39, and 117 nM) for 300 s. After each association step, the dissociation of the GST-(CTD)_26_ was measured for additional 300 s in the BLI buffer. The experiments were performed in triplicates. The association and dissociation constants and the coefficient of determination (R^2^) indicating the appropriateness of the fit were calculated in Octet Analysis Studio Software using 1:2 Bivalent analyte model. The data and the fits were plotted using Prism GraphPad 9 software.

### *In vitro* transcription

The pDRIVE-ITS1 plasmid was purified from 200 ml of *E*. *coli* culture using Plasmid Maxi Kit (Qiagen) and linearized by BamHI enzyme. The linearized plasmid was purified from the agarose gel using QIAquick Gel Extraction Kit (Qiagen) and used as a template for the *in vitro* transcription. For the preparation of the Cy5-labeled ITS1 RNA, 8 μg of the template DNA was mixed with ATP, UTP, and GTP (at 2 mM), CTP (at 1 mM), Cy5-labeled CTP (5-Propargylamino-CTP-Cy5, Jena Bioscience, at 0.05 mM), and T7 RNA polymerase (at 1 μM) in a buffer containing 0.1 M Tris–HCl, pH 8.1; 1% Triton X-100; 16 mM MgCl_2_; 10 mM spermidine; and 50 mM DTT. The reaction was incubated for 4 h at 37 °C. The product was purified using RNeasy Mini Kit (Qiagen) and stored at −80 °C.

### *In vitro* fluorescent labeling of proteins

BRCA1-BARD1, BRCA1^1646-1859^, and BARD1^554-777^, were fluorescently labeled using Alexa Fluor 488 Conjugation Kit - Lightning-Link (Abcam) according to the manufacturer’s instructions. Labeled proteins were snap-frozen in liquid nitrogen and stored at −80 °C. For the *in vitro* LLPS assays, the labeled proteins were mixed with the same protein lacking the fluorescent tag in a 1:20 M ratio.

### Sedimentation assays

Purified BRCA1^1646-1859^ and BARD1^554-777^ (final concentration 80μM, w.t. and the substituted variants, respectively), respectively, were mixed with mCherry-p-hCTD (final concentration 2.5 μM) in the reaction buffer (25 mM Hepes, pH 7.5; 300 mM NaCl; and 1 mM DTT) containing a crowding agent (10% (w/v) dextran). The reactions were incubated 5 min at 23 °C and centrifuged at 130,00*g* for 10 min. The input, supernatant, and pellet fractions were analyzed on SDS-PAGE gel.

### *In vitro* LLPS assays

#### LLPS assays with the full length BRCA1-BARD1 complex

The LLPS assays with nonlabeled and Alexa Fluor 488-conjugated BRCA1-BARD1 (final concentrations 1.25, 2.5, and 5 μM), respectively, were performed in the reaction buffer (25 mM Hepes, pH 7.5; 300 mM NaCl; and 1 mM DTT) in the presence of a crowding agent (10% (w/v) dextran). Where indicated, Cy5-labeled ITS1 RNA (at 15 nM) and/or pS5pS7 phosphorylated hCTD (at 2.5 μM) were added.

#### LLPS assays with BRCTs and pCTD

The LLPS assays with Alexa Fluor 488-conjugated BRCA1^1646-1859^ and BARD1^554-777^ (w.t. and substitution variants at 160 μM), respectively, were performed in the reaction buffer (25 mM Hepes, pH 7.5; 220 mM NaCl; and 1 mM DTT) in the presence of a crowding agent (10% (w/v) dextran). Where indicated, pS5pS7 phosphorylated mCherry-hCTD (at 2.5 μM) was added. The LLPS assays with nonlabeled BRCA1^1646-1859^ and BARD1^554-777^ (w.t. and substitution variants, at 20, 40, 80, and 160 μM), respectively, and pS5pS7 phosphorylated GFP-hCTD (at 2.5 μM) were performed in the reaction buffer (25 mM Hepes, pH 7.5; 220 mM NaCl; 1 mM DTT) in the presence or absence of a crowding agent (10% (w/v) dextran). The combination of 20, 40, 80, and 160 μM BRCT and 2.5 μM phosphorylated GFP-hCTD represents 0.3:1, 0.6:1. 1.2:1, and 2.4:1 ratio of BRCT molecules to its binding sites on CTD (calculated to be 26 for human CTD containing 52 heptapeptide repeats).

#### LLPS assays with BRCTs and ITS1 RNA

The LLPS assays with Alexa Fluor 488-conjugated BRCA1^1646-1859^ and BARD1^554-777^ (w.t. or substitution variants, final concentrations 10, 20, and 40 μM), respectively, and Cy5-labeled ITS1 RNA (final concentration 15 nM) were performed in the reaction buffer (25 mM Hepes, pH 7.5; 220 mM NaCl; 1 mM DTT) in the presence of a crowding agent (10% (w/v) PEG 8000). The LLPS assays with nonlabeled BRCA1^1646-1859^ and BARD1^554-777^ (w.t. and substitution variants, at 160 μM), respectively, and Cy5-labeled ITS1 RNA (final concentration 15 nM) were performed in the reaction buffer (25 mM Hepes, pH 7.5; 220 mM NaCl; 1 mM DTT) in the presence of a crowding agent (10% (w/v) dextran).

#### LLPS assays with BRCTs, ITS1 RNA, and pCTD

The LLPS assays with nonlabeled and Alexa Fluor 488-labeled BRCA1^1646-1859^ and BARD1^554-777^ (concentration 10 μM), respectively, were performed in the reaction buffer (25 mM Hepes, pH 7.5, 220 mM NaCl, and 1 mM DTT) in the presence of a crowding agent (10% (w/v) PEG 8000). Where indicated, Cy5-labeled ITS1 RNA (final concentration 15 nM) and/or pS5pS7 phosphorylated hCTD (final concentration 2.5 μM), and 3 mM MgCl_2_ were added.

#### LLPS assays with BRCT cancer-associated substitution variants

Alexa Fluor 488-conjugated BRCA1^1646-1859^ and BARD1^554-777^ (w.t. or substitution variants, final concentrations 40, 80, and 160 μM) were mixed with a crowding agent (10% (w/v) dextran) in the reaction buffer (25 mM Hepes, pH 7.5, 220 mM NaCl, 1 mM DTT) and incubated for 10 min on ice.

#### Data collection

The mixtures were immediately spotted onto a glass slide, and the condensates were recorded on Zeiss Axio Observer.Z1 with a 63x water immersion objective.

### Statistical analyses

Analyses and quantifications of the micrographs were performed in Cell-Profiler (version 4.2.6.) (119). First, five micrographs (2048 pixels (px) per 2048 px, 1 px = 0.103 μm) per condition and per experiment were analyzed. Objects (droplets) were identified based on diameter (2–70 px, 0,206–7.5 μm) and intensity using Otsu’s method for thresholding. Picked objects were further filtered based on shape and intensity. For the filtered objects the area, median intensity of the objects for the GFP, and mCherry/Cy5 channel, respectively, and the object count per pictures were calculated. For the determination of the colocalization of two fluorescent signals, droplets were first sorted based on the weaker fluorescent signal (median intensity > 0.1), and within this subset, the percentage of droplets also containing the stronger fluorescent signal (median intensity > 0.1) was determined. This approach was necessary to avoid artifacts due to low fluorescence intensity in either channel, which could otherwise lead to an artificially lower colocalization ratio due to the exclusion of droplets with weak signals. The colocalization percentage was calculated as the number of droplets containing both signals divided by the total number of droplets identified in the weaker fluorescence channel. The values for droplets areas were converted from the px to μm based on the metadata of the micrographs (0.010609 μm2 = 1px). Graphs for the figures were plotted using Prism GraphPad 9 software (120). Significance is listed as ∗*p* ≤ 0.05, ∗∗*p* ≤ 0.01, ∗∗∗*p* ≤ 0.001, ∗∗∗∗*p* ≤ 0.0001.

### Crystallization and data collection

Purified BRCA1 BRCT was concentrated to 9.75 mg/ml and mixed with pS5-CTD peptide in 2:1 ratio (final concentrations 6.5 mg/ml and 3.25mg/ml of the BRCT and the CTD peptide, respectively) and crystallized using the sitting-drop vapor diffusion method at 4 °C. The crystals were obtained using the precipitant containing 0.1 M PCB buffer (sodium propionate, sodium cacodylate, and BIS-TRIS propane in the molar ratios 2:1:2), pH = 6, 25% PEG 1500, from the PACT Suite crystallization screen (Qiagen). The crystals were cryoprotected with 40% PEG 400 and frozen in liquid nitrogen. The diffraction data were collected at 100 K at the PETRA III electron storage ring beamline P13 (DESY).

### Structure determination

Diffraction images were processed using the programs XDS (121) and converted to structure factors using the program Scala from the package CCP4 v.8.0 (122), with 5% of the data reserved for the R_free_ calculation. The structure of the complex was solved by molecular replacement with Phaser (123). The BRCA1 BRCT structure (PDB ID: 1JNX) was used as the initial coordinates. The refinement was performed using REFMAC5 (124) alternated with manual model building in Coot v.0.9 (125). The bound peptides were built up manually using Coot. Molecular drawings were prepared using Pymol (Schrödinger, Inc.). The structures were visualized using UCSF ChimeraX package (Resource for Biocomputing, Visualization, and Informatics at the University of California) (126).

### AlphaFold 3 modeling of the BRCT structures

The models of BRCA1^1646-1859^ with pS5 CTD peptide, BARD1^554-777^ with pS5 CTD peptide, BRCA1^1646-1859^ with RNA oligo (ACGGAGCCCG) and BARD1^554-777^ with RNA oligo (ACGGAGCCCG) were built based on the prediction of AlphaFold 3 (79). The top ranked prediction was used for the visualization. Quality of the prediction, as well as the overlay of the five best predictions are depicted in the Figure S29. The predicted structures were deposited to ModelArchive (127) under the accession numbers: ma-xpd7t (BRCA1^1646-1859^ with pS5 CTD peptide), ma-4mmws (BARD1^554-777^ with pS5 CTD peptide), ma-8lz11 (BRCA1^1646-1859^ with RNA oligo), and ma-03j4v (BARD1^554-777^ with RNA oligo).

## Data availability

All primary data are available in this manuscript, supplementary information, and source data. Structural data are available in PDB database under the accession number 9QPX. Source data are available on Zenodo repository, DOI 10.5281/zenodo.17039249.

## Supporting information

This article contains supporting information.

## Conflict of interest

The authors declare that they have no conflicts of interest with the contents of this article.
