# Supplementary material for: Distinct mechanisms of recognition of phosphorylated RNAPII C-terminal domain by BRCT repeats of the BRCA1–BARD1 complex
Source: J Biol Chem. 2025 Dec 5;302(1):111010. doi: 10.1016/j.jbc.2025.111010 (PMC12805184; doi:10.1016/j.jbc.2025.111010)
Supplement: Supplementary Material 1 [file mmc1.pdf]

Distinct Mechanisms of Recognition of Phosphorylated RNAPII C-Terminal Domain by BRCT Repeat of the BRCA1–BARD1 Complex

V. Klapstova, K. Sedova, J. Houser, M. Sebesta

Supplementary material:

Supplementary Figures 1-29

Supplementary Tables 1-4

Supplementary Figure Legends:

Supplementary Figure 1: Purified proteins used in this study

A) SDS-PAGE gels showing purified BRCA1 BRCT variants used in this study.

B) SDS-PAGE gels showing purified BARD1 BRCT variants used in this study.

C) SDS-PAGE gels showing purified CTD variants used in this study**.**

D) SDS-PAGE gel showing purified BRCA1-BARD1 variants used in this study. $BRCA1^{w.t.}$-$BARD1^{w.t.}$ (BRCA1-BARD1), $BRCA1^{2M}$-$BARD1^{w.t.}$ ($BRCA1^{S1655F,\ K1702M}$-BARD1), $BRCA1^{w.t.}$-$BARD1^{2M}$ (BRCA1-$BARD1^{S575F,\ K619A}$), and $BRCA1^{2M}$-$BARD1^{2M}$ ($BRCA1^{S1655F,\ K1702M}$- $BARD1^{S575F,\ K619A}$).

Supplementary Figure 2:

A) SDS-PAGE gel analysis of pull-downs from HEK293 lysates.

B) Western blot analyses of pull-downs from HEK293 lysates. HEK293 cells were lysed and the lysate was cleared by centrifugation. To the supernatant, FLAG-BRCA1-BARD1 was added, and the samples were incubated with α-FLAG beads. As a control, the HEK293 lysate without added BRCA1-BARD1 was used. The proteins were eluted using 3xFLAG peptide and the samples were analysed using western blots. BRCA1-BARD1 interacts with RNAPII via the CTD phosphorylated on Ser2 and Ser5.

Supplementary Figure 3:

A) SDS-PAGE analysis of *in vitro* pull-down assay between GST-$(CTD)_{26}$ and BRCA1-BARD1. Purified BRCA1-BARD1 was incubated with phosphorylated and non-phosphorylated GST-$(CTD)_{26}$ bound to glutathione beads. The samples were centrifuged and the input, unbound (supernatant) and bound (pellet) fractions were analysed using the SDS PAGE. BRCA1-BARD1 interacts directly with GST-pS2pS5-$(CTD)_{26}$ and GST-pS5pS7-$(CTD)_{26}$ *in vitro*.

B) SDS-PAGE analysis of *in vitro* pull-down assay between GST-$(CTD)_{26}$ and BRCA1 BRCT and BARD1 BRCT, respectively. Purified BRCA1 BRCT and BARD1 BRCT, respectively, were incubated with phosphorylated and non-phosphorylated GST-$(CTD)_{26}$ bound to glutathione beads. The samples were centrifuged and the input, unbound (supernatant) and bound (pellet) fractions were analysed using the SDS PAGE. Substitutions in the phosphoserine binding site of the BRCT domain ($BRCA1^{S1655F,K1702M}$, $BARD1^{S575F,K619A}$)abolish the binding.

C) SDS-PAGE analysis of *in vitro* pull-down assay between GST-pS5pS7-$(CTD)_{26}$ and $BRCA1^{w.t.}$-$BARD1^{w.t.}$ (BRCA1-BARD1), $BRCA1^{2M}$-$BARD1^{w.t.}$ ($BRCA1^{S1655F,K1702M}$-BARD1), $BRCA1^{w.t.}$-$BARD1^{2M}$ (BRCA1-$BARD1^{S575F,K619A}$), and $BRCA1^{2M}$-$BARD1^{2M}$ ($BRCA1^{S1655F,K1702M}$- $BARD1^{S575F,K619A}$), respectively. Purified BRCA1-BARD1 variants were incubated with GST-pS5pS7-$(CTD)_{26}$ bound to glutathione beads. The samples were centrifuged and the input, unbound (supernatant) and bound (pellet) fractions were analysed

using the SDS PAGE. The inactivation of single tandem BRCT repeat (either BRCA1 or BARD1 BRCT, respectively) lead to the reduction of binding.

Supplementary Figure 4:

A) Multiple sequence alignment of amino-acids present in phosphopeptide-binding sites of BRCA1 BRCT domains from various organisms. The alignment was generated using Clustal Omega on EMBL-EBI and visualised using Jalview.

B) Multiple sequence alignment of amino-acids present in phosphopeptide-binding sites of BARD1 BRCT domains from various organisms. The alignment was generated using Clustal Omega on EMBL-EBI and visualised using Jalview.

Supplementary Figure 5:

A) Multiple sequence alignment of sequences of BRCA1 BRCT domains from various organisms. The alignment was generated using Clustal Omega on EMBL-EBI and visualised using Jalview.

B) Multiple sequence alignment of sequences of BARD1 BRCT domains from various organisms. The alignment was generated using Clustal Omega on EMBL-EBI and visualised using Jalview.

Supplementary Figure 6:

A) Sensorgrams obtained by biolayer-interferometry (BLI) analysing interactions of BRCA1 $BRCT^{S1655F,K1702M}$, BRCA1 $BRCT^{R1699L}$, and BARD1 $BRCT^{S575F,K619A}$, respectively, with GST-pS5pS7-$(CTD)_{26}$ and GST-$(CTD)_{26}$. The sensorgrams represent the mean of 3 measurements for each concentration. The data were analysed in Octet® Analysis Studio Software using 1:2 Bivalent analyte model. The data were plotted using Prism GraphPad 9 software.

B) Structural alignment of BRCA1 BRCT (1JNX) and BARD1 BRCT (2NTE) obtained in UCSF Chimera.

C) Sensorgrams obtained by biolayer-interferometry (BLI) analysing interactions of BRCA1 $BRCT^{R1699S}$, BRCA1 $BRCT^{M1775H}$, and BARD1 $BRCT^{H686M}$, respectively, with GST-pS5pS7-$(CTD)_{26}$. The sensorgrams represent the mean of 3 measurements for each concentration. The data were analysed in Octet® Analysis Studio Software using 1:2 Bivalent analyte model. The data were plotted using Prism GraphPad 9 software.

Supplementary Figure 7:

A) Specificity of the BRCA1 BRCT phospho-peptide binding site determined by fluorescence anisotropy measurement. The assays were performed between BRCA1 BRCT domain and the indicated ligands (at 25nM). Anisotropy data were plotted as a function of protein concentration and fitted to a single-site saturation with non-specific binding model using XMGrace.

B) Comparison of the apo-structure of BRCA1 BRCT and the structure of BRCA1 BRCT with bound pS5 CTD peptide (YSPTpSPSYSPTpSPS). Teal: newly determined structure of BRCA1 BRCT in complex with pCTD (PDB ID: 9QPX), Grey: BRCA1 BRCT apo-structure (PDB: 1JNX). Structural alignment was generated in UCSF ChimeraX using the Match maker tool.

C) Comparison of the phosphoserine binding site of BRCA1 BRCT with (teal, PDB ID: 9QPX) and without (gray, PDB ID: 1JNX) bound pS5 CTD peptide (yellow).

D) Distribution of the surface charge of BRCA1 BRCT with docked pS5 CTD peptide. The electrostatic potential was calculated by the Adaptive Poisson-Boltzmann Solver and visualised in Pymol.

E) Detail of the binding site of BRCA1 BRCT with the electron density map representing the pS5 CTD peptide present in the chains A, B and C (left, middle and right, respectively). Structural models and electron density were visualised using Coot (version 0.9.8.93).

Supplementary Figure 8:

A) Comparison of the crystal structure of the BRCA1 BRCT (dark gray) with the pS5 CTD ligand (dark yellow), PDB ID: 9QPX, and the AlphaFold 3 model of the complex (BRCA1 BRCT – light gray, S5 pCTD peptide – light yellow).

B) Detail of the phosphoserine binding site (left) of BRCA1 BRCT (gray) with bound pS5 CTD peptide (yellow) modelled by AlphaFold 3. The pS5 of the CTD peptide is depicted in purple, the amino-acid residues interacting with pS5 are depicted in light violet. The hydrogen bonds were displayed as pseudobonds using the structural analysis tool Hydrogen bonds in UCSF ChimeraX and are depicted in turquoise. The relax distance tolerance was 1.0 Å and the relax angle tolerance was 20.0°. Detail of the aromatic amino-acid binding pocket (middle, right) of BRCA1 BRCT (gray) with bound pS5 CTD peptide (yellow). The Y1 of the CTD is depicted in navy, the interacting amino-acid residues in light blue. The hydrogen bonds (middle) were displayed as pseudobonds using the structural analysis tool Hydrogen bonds in UCSF ChimeraX and are depicted in turquoise. The relax distance tolerance was 1.0 Å and the relax angle tolerance was 20.0°. Van der Waals, hydrophobic and stacking interactions (right) were displayed as pseudobonds using the structural analysis tool in UCSF ChimeraX and are depicted in black. The interacting atoms were identified based on VDW overlap ≥ -0.4 Å.

C) AlphaFold 3 model of the BARD1 BRCT (gray) with the S5 pCTD (orange).

D) Detail of the phosphoserine binding site (left) of BARD1 BRCT (gray) with bound pS5 CTD peptide (yellow) modelled by AlphaFold3. The pS5 of the CTD peptide is depicted in purple, the amino-acid residues interacting with pS5 are depicted in light violet. The hydrogen bonds were displayed as pseudobonds using the structural analysis tool Hydrogen bonds in UCSF ChimeraX and are depicted in turquoise. The relax distance tolerance was 1.0 Å and the relax angle tolerance was 20.0°. Detail of the aromatic amino-acid binding pocket (middle, right) of BARD1 BRCT (gray) with bound pS5 CTD peptide (yellow). The Y1 of the CTD is depicted in navy, the interacting amino-acid residues in light blue. The hydrogen bonds (middle) were displayed as pseudobonds using the structural analysis tool Hydrogen bonds in UCSF ChimeraX and are depicted in turquoise. The relax distance tolerance was 1.0 Å and the relax angle tolerance was 20.0°. Van der Waals, hydrophobic and stacking interactions (right)

were displayed as pseudobonds using the structural analysis tool in UCSF ChimeraX and are depicted in black. The interacting atoms were identified based on VDW overlap ≥ -0.4 Å.

Supplementary Figure 9:

A) LLPS assays with purified BRCA1-BARD1 complex. BRCA1-BARD1 (at 5 μM) was mixed with phosphorylated mGFP-p-hCTD (2.5 μM) and/or Cy5-ITS1 RNA (at 15 nM) in the presence of a crowding agent (10% dextran). Representative images from three experiments (bottom) are depicted as an overlay of differential interference contrast (DIC), GFP, and Cy5. Scale bars, 10 μm.

B) Bar chart (top) representing quantification (n = 3) of the number of droplets per frame from the LLPS experiments with the BRCA1-BARD1 complex shown in (A). Statistical significance was determined by unpaired t-test. A nested scatterplot (bottom) represents quantification (n = 3) of an area of individual droplets from three independent experiments with the BRCA1-BARD1 complex shown in (A), with median area determined per dataset. Statistical significance was determined by nested t-test.

C) Effect of the Alexa-488 labelling on the LLPS propensities of the BRCA1-BARD1 complex. Bar chart (top) representing quantification (n = 3) of the number of droplets per frame from the LLPS experiments with BRCA1-BARD1, mCherry-p-hCTD, and Cy5-RNA. Statistical significance was determined by unpaired t-test. A nested scatterplot (middle) represents quantification (n = 3) of an area of individual droplets from three independent experiments with BRCA1-BARD1, with median area determined per dataset. Statistical significance was determined by nested t-test. Representative images from three experiments (bottom) are depicted as an overlay of differential interference contrast (DIC) and GFP. Scale bars, 10 μm.

Supplementary Figure 10:

A) LLPS assays with purified BRCA1 BRCT and mGFP-p-hCTD. BRCA1 BRCT w.t., BRCA1 BRCT^{S1655F,K1702M}, BRCA1 BRCT^{R1699L}, and BRCA1 BRCT^{M1775K}, respectively, present at the indicated protein to CTD binding site ratio, were mixed with phosphorylated CTD (2.5 μM) in the presence of a crowding agent (10% dextran). Representative images from three experiments are depicted as an overlay of differential interference contrast (DIC) and GFP. Scale bars, 10 μm.

B) Bar chart (top) representing quantification (n = 3) of the number of droplets per frame from the LLPS experiments with BRCA1 BRCT w.t. shown in (A). Statistical significance was determined by unpaired t-test. A nested scatterplot (bottom) represents quantification (n = 3) of an area of individual droplets from three independent experiments with BRCA1 BRCT w.t. shown in (A)., with median area determined per dataset. Statistical significance was determined by nested t-test.

C) LLPS assays with purified BARD1 BRCT and mGFP-p-hCTD. BARD1 BRCT w.t. and BARD1 BRCT^{S575F,K619A}, respectively, present at the indicated protein to CTD binding site ratio, were mixed with phosphorylated CTD (2.5 μM) in the presence of a crowding agent (10% dextran). Representative images from three experiments are depicted as an overlay of differential interference contrast (DIC) and GFP. Scale bars, 10 μm.

D) Bar chart (top) representing quantification (n = 3) of the number of droplets per frame from the LLPS experiments with BARD1 BRCT w.t. shown in (C). Statistical significance was determined by unpaired t-test. A nested scatterplot (bottom) represents quantification (n = 3) of an area of individual droplets from three independent experiments with BARD1 BRCT w.t. shown in (C)., with median area determined per dataset. Statistical significance was determined by nested t-test.

Supplementary Figure 11:

A) Scan of representative gel from EMSA experiments performed with Cy3-labelled RNA and BRCA1 BRCT w.t. and BRCA1 BRCT^{S1655F,K1702M}, respectively.

B) Scan of representative gels from EMSA experiments performed with Cy3-labelled RNA and BARD1 BRCT w.t., BARD1 BRCT^{S575F,K619A}, and BARD1 BRCT^{R705A}, respectively.

C) Scan of representative gels from EMSA experiments performed with Cy3-labelled RNA or Cy3-labelled dsDNA and BRCA1 BRCT or BRCA1 DNA-binding domain (DBD).

D) Scan of representative gels from competition EMSA experiments performed with Cy3-labelled RNA, , and Cy3-labelled dsDNA, respectively, with BRCA1 BRCT and BARD1 BRCT, respectively, in the presence of GST-pS5pS7-(CTD)$_{26}$.

Supplementary Figure 12:

A) Graphs representing quantification of EMSA experiments (n = 3) shown in Supplementary figure 11A,B. performed with Cy3-labelled RNA and BRCA1 BRCT (w.t. and substitution variant, left) and BARD1 BRCT (w.t. and substitution variants, right), respectively. Statistical significance was determined using unpaired Student's t-test.

B) Bar charts representing the quantification of binding of RNA and double-stranded DNA to BRCA1 BRCT and BARD1 BRCT, respectively, in the presence of GST-pS5pS7-(CTD)$_{26}$, which was shown in (Supplementary figure 11 D). Statistical significance was determined using unpaired Student's t-test.

C) Graphs representing quantification of EMSA experiments (n = 3) shown in (Supplementary figure 11 C), comparing the binding affinity of BRCA1 BRCT and BRCA1 DNA-binding domain (DBD) to Cy3-labelled RNA and double-stranded DNA.

D) AlphaFold 3-generated models of RNA binding to the BRCA1 BRCT (left) and BARD1 BRCT (right). BRCA1 BRCT interacts with RNA through the phosphopeptide-binding site, whilst BARD1 BRCT through an alternative binding-site.

E) Detail of the RNA binding sites (bottom) of the BRCA1 BRCT (left) and BARD1 BRCT (right). The hydrogen bonds were displayed as pseudobonds using the structural analysis tool Hydrogen bonds in UCSF ChimeraX and are depicted in turquoise. The relax distance tolerance was 1.0 Å and the relax angle tolerance was 20.0°. Van der Waals, hydrophobic and stacking interactions (right) were displayed as pseudobonds using the structural analysis tool in UCSF ChimeraX and are depicted in black. The interacting atoms were identified based on VDW overlap ≥ -0.4 Å.

Supplementary Figure 13:

A) Comparison of the surface charges of BRCA1 BRCT (left) and BARD1 BRCT (right). The electrostatic potential was calculated by the Adaptive Poisson-Boltzmann Solver and visualised in Pymol.

B) Multiple sequence alignment of amino-acids, present in putative RNA-binding sites of BARD1 BRCT domains from various organisms. The alignment was generated using Clustal Omega on EMBL-EBI and visualised using Jalview.

Supplementary Figure 14:

A) LLPS assays with purified, Alexa488-labelled BRCT domains and Cy5-ITS1 RNA. BRCA1 BRCT (left, at 10, 20, and 40 µM) and BARD1 BRCT (right, at 10, 20, and 40 µM), respectively, were mixed with ITS1 RNA (15nM) in the presence of a crowding agent (10% PEG 8000). Representative images from three experiments are depicted as an overlay of differential interference contrast (DIC), Alexa488, and Cy5 signal. Scale bars, 10 µm.

B) Bar chart (top) representing quantification ($n = 3$) of the number of droplets per frame from the LLPS experiments with BRCA1 BRCT (left) and BARD1 BRCT (right), shown in (A). Statistical significance was determined by unpaired t-test. A nested scatterplot (middle) representing quantification ($n = 3$) of an area of individual droplets from three independent experiments with BRCA1 BRCT (left) and BARD1 BRCT (right), shown in (A), with median area determined per dataset. Statistical significance was determined by nested t-test. Bar chart (bottom) depicting the proportion of droplets containing signals for Cy5-ITS1 RNA (red) and Alexa488-labelled BRCA1 BRCT (green, left) and Alexa488-labelled BARD1 BRCT (green, right), respectively.

C) Effect of the Alexa-488 labelling on the LLPS propensity of BRCT domains. BRCA1 BRCT (left, non-labelled or Alexa488-labelled, at 160 µM) and BARD1 BRCT (right, non-labelled or Alexa488-labelled, at 160 µM), respectively, were mixed with Cy5-labelled ITS1 RNA (15nM) in the presence of a crowding agent (10% dextran). Representative images from three experiments are depicted as an overlay of differential interference contrast (DIC), and Cy5 signal. Scale bars, 10 µm.

D) Effect of the Alexa-488 labelling on the LLPS propensity of BRCT domains. Bar chart (top) representing quantification ($n = 3$) of the number of droplets per frame from the LLPS experiments with BRCT domains and ITS1 RNA shown in (C). Statistical significance was determined by unpaired t-test. A nested scatterplot (middle) representing quantification ($n = 3$) of an area of individual droplets from three independent experiments with BRCT domains and ITS1 RNA shown in (C), with median area determined per dataset. Statistical significance was determined by nested t-test.

Supplementary Figure 15:

A) LLPS assays with purified BRCT domains and Cy5-ITS1 RNA. BRCA1 BRCT w.t. and BRCA1 $BRCT^{S1655F,K1702M}$, respectively, (at 160 µM, left) and BARD1 BRCT w.t., BARD1 $BRCT^{S575F,K619A}$, and BARD1 $BRCT^{R705A}$, respectively, (at 160 µM, right), were mixed with Cy5-labelled ITS1 RNA (15nM) in the presence of a crowding agent (10% dextran).

Representative images from three experiments are depicted as an overlay of differential interference contrast (DIC), and Cy5 signal. Scale bars, 10 μm.

B) Bar chart (top) representing quantification (n = 3) of the number of droplets per frame from the LLPS experiments with BRCT domains and ITS1 RNA, shown in (A). Statistical significance was determined by unpaired t-test. A nested scatterplot (bottom) representing quantification (n = 3) of an area of individual droplets from three independent experiments with BRCT domains and ITS1 RNA shown in (A), with median area determined per dataset. Statistical significance was determined by nested t-test.

C) LLPS assays with Cy5-ITS1 RNA (top). Cy5-labelled ITS1 RNA (15nM) was mixed with the crowding agent (10% dextran). Representative images from three experiments are depicted as an overlay of differential interference contrast (DIC), and Cy5 signal. Scale bars, 10 μm. Bar chart (middle) representing quantification (n = 3) of the number of droplets per frame from the LLPS experiments ITS1 RNA. A nested scatterplot (bottom) representing quantification (n = 3) of an area of individual droplets from three independent experiments with ITS1 RNA, with median area determined per dataset.

Supplementary Figure 16:

A) SDS-PAGE gels analysis of cross-linked BRCA1 BRCT and BARD1 BRCT samples, respectively, showing the multimerization of BRCT domains.

B) SDS-PAGE gel analysis of sedimentation assays with BRCT domains. Purified BRCA1 BRCT and BARD1 BRCT (w.t. and substituted variants, final concentration 80 μM), were mixed with mCherry-p-hCTD (final concentration 2.5 μM) in the presence of a crowding agent (10% dextran). The reactions were incubated 5 min and centrifuged. BARD1 BRCT 2M denotes variant BARD1 BRCT^{S575F,K619A}, BRCA1 BRCT 2M denotes variant BRCA1 BRCT^{S1655F,K1702M}.

Supplementary Figure 17:

A) LLPS assays with purified BRCA1 BRCT, mGFP-p-hCTD, and Cy5-ITS1 RNA. BRCA1 BRCT (at 10 μM) was mixed with phosphorylated pCTD (2.5 μM), and/or ITS1 RNA (15nM) in the presence of a crowding agent (10% PEG 8000). Where indicated, $MgCl_2$ was present (at 3mM). Representative images from three experiments are depicted as an overlay of differential interference contrast (DIC), GFP and Cy5 signal. Scale bars, 10 μm.

B) Bar chart (top) representing quantification (n = 3) of the number of droplets per frame from the LLPS experiments with BRCA1 BRCT, mGFP-p-hCTD, and Cy5-ITS1 RNA, shown in (A), using the green (left) or red (right) fluorescent signal. Statistical significance was determined by unpaired t-test. A nested scatterplot (middle) representing quantification (n = 3) of an area of individual droplets from three independent experiments with BRCA1 BRCT, mGFP-p-hCTD, and Cy5-ITS1 RNA, shown in (A), with median area determined per dataset. Statistical significance was determined by nested t-test. Bar chart (bottom) depicting the proportion of droplets containing signals for mGFP-p-hCTD (green) and Cy5-ITS1 RNA (red).

C) LLPS assays with purified BARD1 BRCT, mGFP-p-hCTD, and Cy5-ITS1 RNA. BARD1 BRCT (at 10 μM) was mixed with phosphorylated CTD (2.5 μM), and/or ITS1 RNA (15nM) in the presence of a crowding agent (10% PEG 8000). Where indicated, $MgCl_2$ was present (at 3mM). Representative images from three experiments are depicted as an overlay of differential interference contrast (DIC), GFP, and Cy5 signal. Scale bars, 10 μm.

D) Bar chart (top) representing quantification (n = 3) of the number of droplets per frame from the LLPS experiments with BARD1 BRCT, mGFP-p-hCTD, and Cy5-ITS1 RNA, shown in (C), using the green (left) or red (right) fluorescent signal. Statistical significance was determined by unpaired t-test. A nested scatterplot (middle) representing quantification (n = 3) of an area of individual droplets from three independent experiments with BARD1 BRCT, mGFP-p-hCTD, and Cy5-ITS1 RNA, shown in (C), with median area determined per dataset. Statistical significance was determined by nested t-test. Bar chart (bottom) depicting the proportion of droplets containing signals for mGFP-p-hCTD (green) and Cy5-ITS1 RNA (red).

Supplementary Figure 18:

A) LLPS assays with purified BRCA1 BRCT, mGFP-p-hCTD, and Cy5-ITS1 RNA. BRCA1 BRCT w.t and BRCA1 BRCT^{S1655F,K1702M} (at 10 μM), respectively, were mixed with phosphorylated CTD (2.5 μM), and ITS1 RNA (15nM) in the presence of a crowding agent (10% PEG 8000). Representative images from three experiments are depicted as an overlay of differential interference contrast (DIC), GFP, and Cy5 signal. Scale bars, 10 μm.

B) Bar chart (top) representing quantification (n = 3) of the number of droplets per frame from the LLPS experiments with BRCA1 BRCT, mGFP-p-hCTD, and Cy5-ITS1 RNA, shown in (A), using the green or red fluorescent signal. Statistical significance was determined by unpaired t-test. A nested scatterplot (bottom) representing quantification (n = 3) of an area of individual droplets from three independent experiments with BRCA1 BRCT, mGFP-p-hCTD, and Cy5-ITS1 RNA, shown in (A)., with median area determined per dataset. Statistical significance was determined by nested t-test.

C) LLPS assays with purified BARD1 BRCT, mGFP-p-hCTD, and Cy5-ITS1 RNA. BARD1 BRCT w.t., BARD1 BRCT^{S575F,K619A}, and BARD1 BRCT^{R705A} (at 10 μM), respectively, were mixed with phosphorylated CTD (2.5 μM), and ITS1 RNA (15nM) in the presence of a crowding agent (10% PEG 8000). Representative images from three experiments are depicted as an overlay of differential interference contrast (DIC), GFP, and Cy5 signal. Scale bars, 10 μm.

D) Bar chart (top) representing quantification (n = 3) of the number of droplets per frame from the LLPS experiments with BARD1 BRCT, mGFP-p-hCTD, and Cy5-ITS1 RNA, shown in (C), using the green or red fluorescent signal. Statistical significance was determined by unpaired t-test. A nested scatterplot (bottom) representing quantification (n = 3) of an area of individual droplets from three independent experiments with BARD1 BRCT, mGFP-p-hCTD, and Cy5-ITS1 RNA, shown in (C), with median area determined per dataset. Statistical significance was determined by nested t-test.

E) Sensorgrams obtained by biolayer-interferometry (BLI) (left). Comparison of association (k_{as}) and dissociation (k_{dis}) kinetic constants, and equilibrium dissociation (K_D) constants for GST-pS5pS7-$(CTD)_{26}$ and BARD1 BRCT w.t. and BARD1 BRCT^{R705A}, respectively, obtained by biolayer interferometry (right). The sensorgrams represent the mean of 3 measurements for

each concentration. The data were analysed in Octet® Analysis Studio Software using 1:2 Bivalent analyte model. The data were plotted using Prism GraphPad 9 software.

F) Comparison of sensorgrams obtained by biolayer-interferometry (BLI) with BARD1 BRCT w.t., and BARD1 BRCT^{R705A}, and their respective fits (left). Comparison of association (k_{as}) and dissociation (k_{dis}) kinetic constants, and equilibrium dissociation (K_D) constants for GST-pS5pS7-$(CTD)_{26}$ and BARD1 BRCT w.t., and BARD1 BRCT^{R705A}, respectively, obtained by biolayer interferometry (right).

Supplementary Figure 19:

A) Conservation of the selected positions in BRCA1 BRCT with disease-associated substitutions. Multiple sequence alignment of sequences of BRCA1 BRCT domains from various organisms was generated using Clustal Omega on EMBL-EBI and visualised using Jalview.

B) Conservation of the selected positions in BARD1 BRCT that with disease-associated substitutions. Multiple sequence alignment of sequences of BARD1 BRCT domains from various organisms was generated using Clustal Omega on EMBL-EBI and visualised using Jalview.

Supplementary Figure 20:

A) Quantification of the thermal stability of BRCA1 BRCT w.t., BRCA1 BRCT^{E1682K}, BRCA1 BRCT^{R1699L}, BRCA1 BRCT^{R1699W}, BRCA1 BRCT^{E1754K}, BARD1 BRCT w.t., BARD1 BRCT^{E587K}, BARD1 BRCT^{E665K}, BARD1 BRCT^{S711R}, and BARD1 BRCT^{K754N} measured by nanoDSF. The bar charts depict the temperatures corresponding to the onset of protein unfolding (T_o), the melting temperature (T_m), and the onset of aggregation (T_{agg}) from n = 9 measurements. Statistical significance was determined by unpaired t-test.

B) Comparison of the thermal stability of BRCA1 BRCT w.t., and BARD1 BRCT w.t. determined by nanoDSF. The bar charts depict the temperatures corresponding to the onset of protein unfolding (T_o), the melting temperature (T_m), and the onset of aggregation (T_{agg}) from n = 9 measurements. Statistical significance was determined by unpaired t-test.

Supplementary Figure 21:

A) Sensorgrams obtained by biolayer-interferometry (BLI) with BRCA1 BRCT^{E1754K} . The sensorgrams represent the mean of 3 measurements for each concentration. The data were analysed in Octet® Analysis Studio Software using 1:2 Bivalent analyte model. The data were plotted using Prism GraphPad 9 software.

B) Comparison of sensorgrams obtained by biolayer-interferometry (BLI) with BRCA1 BRCT w.t. and BRCA1 BRCT^{E1754K} and their respective fits (left). Comparison of association (k_{as}) and dissociation (k_{dis}) kinetic constants, and equilibrium dissociation (K_D) constants for GST-pS5pS7-$(CTD)_{26}$ and BRCA1 BRCT w.t. and BRCA1 BRCT^{E1754K}, respectively, obtained by biolayer interferometry (right). Cancer-associated substitution BRCA1 BRCT^{E1754K} does not affect the binding of the GST-pS5pS7-$(CTD)_{26}$.

C) Sensorgrams obtained by biolayer-interferometry (BLI) with BARD1 BRCT^{E665K} (left) and BARD1 BRCT^{K754N} (right). The sensorgrams represent the mean of 3 measurements for each concentration. The data were analysed in Octet® Analysis Studio Software using 1:2 Bivalent analyte model. The data were plotted using Prism GraphPad 9 software.

D) Comparison of sensorgrams obtained by biolayer-interferometry (BLI) with BARD1 BRCT w.t., BARD1 BRCT^{E665K}, and BARD1 BRCT^{K754N} and their respective fits (left). Comparison of association (k_{as}) and dissociation (k_{dis}) kinetic constants, and equilibrium dissociation (K_D) constants for GST-pS5pS7-$(CTD)_{26}$ and BARD1 BRCT w.t., BARD1 BRCT^{E665K}, and BARD1 BRCT^{K754N}, respectively, obtained by biolayer interferometry (right). Cancer-associated substitutions BARD1 BRCT^{E665K}, and BARD1 BRCT^{K754N} do not affect the binding of the GST-pS5pS7-$(CTD)_{26}$.

Supplementary Figure 22:

A) Bar charts (top) representing quantification (n = 3) of the number of droplets per frame from the LLPS experiments with BRCA1 BRCT^{E1682K}, and BRCA1 BRCT^{E1754K} shown in (Fig. 6B). Statistical significance was determined by unpaired t-test. Nested scatterplots (bottom) representing quantification (n = 3) of an area of individual droplets from three independent experiments with BRCA1 BRCT^{E1682K}, and BRCA1 BRCT^{E1754K}, respectively, shown in (Fig. 6B), with median area determined per dataset. Statistical significance was determined by nested t-test.

B) Bar charts (top) representing quantification (n = 3) of the number of droplets per frame from the LLPS experiments with BARD1 BRCT^{E587K}, BARD1 BRCT^{E665K}, BARD1 BRCT^{S711R}, and BARD1 BRCT^{K754N} shown in (Fig. 6D). Statistical significance was determined by unpaired t-test. Nested scatterplots (bottom) representing quantification (n = 3) of an area of individual droplets from three independent experiments with BARD1 BRCT^{E587K}, BARD1 BRCT^{E665K}, BARD1 BRCT^{S711R}, and BARD1 BRCT^{K754N}, respectively, shown in (Fig. 6D), with median area determined per dataset. Statistical significance was determined by nested t-test.

Supplementary Figure 23:

A) LLPS assays with purified, Alexa488-labelled BRCT domains. BRCA1 BRCT w.t. and BRCA1 BRCT^{E1754K}, respectively, (at 40 µM, 80 µM, 160 µM, left), were mixed with a crowding agent (10% dextran). BARD1 BRCT w.t. and BARD1 BRCT^{K754N}, respectively, (at 40 µM, 80 µM, 160 µM, right), were mixed with a crowding agent (10% dextran). Representative images from three experiments are depicted as an overlay of differential interference contrast (DIC) and Alexa488 signal. Scale bars, 10 µm.

B) Aggregation of purified, Alexa-488 labelled BARD1 BRCT^{E665K}. BARD1 BRCT^{E665K} (160 µM) was mixed with a crowding agent (10% dextran). Representative image from three experiments is depicted as an overlay of differential interference contrast (DIC) and Alexa488 signal. Scale bar, 10 µm.

C) SDS-PAGE gels with cross-linked samples of BRCA1 BRCT and BARD1 BRCT disease-associated variants, comparing the multimerization of w.t. proteins and their respective disease-associated variants.

D) Bar charts (top) representing quantification (n = 3) of the number of droplets per frame from the LLPS experiments with BRCA1 BRCT w.t. and BRCA1 $BRCT^{E1754K}$, respectively (left), and BARD1 BRCT w.t. and BARD1 $BRCT^{K754N}$, respectively (right), shown in (A). Statistical significance was determined by unpaired t-test. Nested scatterplots (bottom) representing quantification (n = 3) of an area of individual droplets from three independent experiments with BRCA1 BRCT w.t. and BRCA1 $BRCT^{E1754K}$, respectively (left), and BARD1 BRCT w.t. and BARD1 $BRCT^{K754N}$, respectively (right), shown in (A), with median area determined per dataset. Statistical significance was determined by nested t-test.

Supplementary figure 24:

A) SDS-PAGE gel analysis of sedimentation assays with BRCA1 BRCT in the presence (right) or absence (left) of phosphorylated CTD. Purified BRCA1 BRCT w.t., BRCA1 $BRCT^{E1682K}$, and BRCA1 $BRCT^{E1754K}$, respectively (final concentration 80 µM), were mixed with mCherry-p-hCTD (final concentration 2.5 µM) in the presence of a crowding agent (10% dextran). The reactions were incubated 5 min and centrifuged.

B) SDS-PAGE gel analysis of sedimentation assays with BARD1 BRCT in the presence (bottom) or absence (top) of phosphorylated CTD. Purified BARD1 BRCT w.t., BARD1 $BRCT^{E665K}$, BARD1 $BRCT^{S711R}$, and BARD1 $BRCT^{K754N}$, respectively (final concentration 80 µM), were mixed with mCherry-p-hCTD (final concentration 2.5 µM) in the presence of a crowding agent (10% dextran). The reactions were incubated 5 min and centrifuged.

Supplementary figure 25:

A) Scan of a representative gel from EMSA experiments performed with Cy3-labelled RNA and BARD1 BRCT w.t., and BARD1 $BRCT^{S711R}$, respectively.

B) Graph representing quantification of EMSA experiments (n = 3) shown in (A) performed with Cy3-labelled RNA and BARD1 BRCT w.t., and BARD1 $BRCT^{S711R}$, respectively. Statistical significance was determined using unpaired Student's t-test.

C) LLPS assays with purified, Alexa488-labelled BRCT domains and Cy5-ITS1 RNA. BARD1 BRCT w.t., and BARD1 $BRCT^{S711R}$, respectively (at 160 µM), were mixed with Cy5-labelled ITS1 RNA (15nM) in the presence of a crowding agent (10% dextran). Representative images from three experiments are depicted as an overlay of differential interference contrast (DIC), and Cy5 signal. Scale bars, 10 µm.

D) Bar chart (top) representing quantification (n = 3) of the number of droplets per frame from the LLPS experiments with BARD1 BRCT and ITS1 RNA, shown in (C). Statistical significance was determined by unpaired t-test. A nested scatterplot (bottom) representing quantification (n = 3) of an area of individual droplets from three independent experiments with BARD1 BRCT shown in (C), with median area determined per dataset. Statistical significance was determined by nested t-test.

Supplementary Figure 26:

A) LLPS assays with purified BRCA1-BARD1, mGFP-p-hCTD, and Cy5-ITS1 RNA. BRCA1-BARD1 (at 5 µM) was mixed with phosphorylated pCTD (at 1.25 µM, 2.5 µM, and 5 µM, respectively), and ITS1 RNA (15 nM) in the presence of a crowding agent (10% PEG

8000) and $MgCl_2$ (at 3 mM). Representative images from three experiments are depicted as an overlay of differential interference contrast (DIC), GFP and Cy5 signal. Scale bars, 10 µm.

B) Bar chart (top) representing quantification (n = 3) of the number of droplets per frame from the LLPS experiments with BRCA1-BARD1, mGFP-p-hCTD, and Cy5-ITS1 RNA, shown in (A), using the green (left) or red (right) fluorescent signal. Statistical significance was determined by unpaired t-test. A nested scatterplot (middle) representing quantification (n = 3) of an area of individual droplets from three independent experiments with BRCA1-BARD1, mGFP-p-hCTD, and Cy5-ITS1 RNA, shown in (A), with median area determined per dataset. Statistical significance was determined by nested t-test. Bar chart (bottom) depicts the proportion of droplets containing signals for mGFP-p-hCTD (green) and Cy5-ITS1 RNA (red).

C) LLPS assays with purified BRCA1-BARD1, mGFP-p-hCTD, and Cy5-ITS1 RNA. BRCA1-BARD1 (at 5 µM) was mixed with phosphorylated pCTD (at 2.5 µM), and ITS1 RNA (at 7.5 nM, 15 nM, and 30 nM, respectively) in the presence of a crowding agent (10% PEG 8000) and $MgCl_2$ (at 3 mM). Representative images from three experiments are depicted as an overlay of differential interference contrast (DIC), GFP and Cy5 signal. Scale bars, 10 µm.

D) Bar chart (top) representing quantification (n = 3) of the number of droplets per frame from the LLPS experiments with BRCA1-BARD1, mGFP-p-hCTD, and Cy5-ITS1 RNA, shown in (C), using the green (left) or red (right) fluorescent signal. Statistical significance was determined by unpaired t-test. A nested scatterplot (bottom) representing quantification (n = 3) of an area of individual droplets from three independent experiments with BRCA1-BARD1, mGFP-p-hCTD, and Cy5-ITS1 RNA, shown in (C), with median area determined per dataset. Statistical significance was determined by nested t-test. Bar chart (bottom) depicts the proportion of droplets containing signals for mGFP-p-hCTD (green) and Cy5-ITS1 RNA (red).

Supplementary Figure 27:

A) LLPS assays with purified BRCA1 BRCT, mGFP-p-hCTD, and Cy5-ITS1 RNA. BRCA1 BRCT (at 10 µM) was mixed with phosphorylated pCTD (at 1.25 µM, 2.5 µM, and 5 µM, respectively), and ITS1 RNA (15 nM) in the presence of a crowding agent (10% PEG 8000) and $MgCl_2$ (at 3 mM). Representative images from three experiments are depicted as an overlay of differential interference contrast (DIC), GFP and Cy5 signal. Scale bars, 10 µm.

B) Bar chart (top) representing quantification (n = 3) of the number of droplets per frame from the LLPS experiments with BRCA1 BRCT, mGFP-p-hCTD, and Cy5-ITS1 RNA, shown in (A), using the green (left) or red (right) fluorescent signal. Statistical significance was determined by unpaired t-test. A nested scatterplot (middle) representing quantification (n = 3) of an area of individual droplets from three independent experiments with BRCA1 BRCT, mGFP-p-hCTD, and Cy5-ITS1 RNA, shown in (A), with median area determined per dataset. Statistical significance was determined by nested t-test. Bar chart (bottom) depicts the proportion of droplets containing signals for mGFP-p-hCTD (green) and Cy5-ITS1 RNA (red).

C) LLPS assays with purified BRCA1 BRCT, mGFP-p-hCTD, and Cy5-ITS1 RNA. BRCA1 BRCT (at 10 µM) was mixed with phosphorylated pCTD (at 2.5 µM), and ITS1 RNA (at 7.5 nM, 15 nM, and 30 nM, respectively) in the presence of a crowding agent (10% PEG 8000) and $MgCl_2$ (at 3 mM). Representative images from three experiments are depicted as an overlay of differential interference contrast (DIC), GFP and Cy5 signal. Scale bars, 10 µm.

D) Bar chart (top) representing quantification (n = 3) of the number of droplets per frame from the LLPS experiments with BRCA1 BRCT, mGFP-p-hCTD, and Cy5-ITS1 RNA, shown in (C), using the green (left) or red (right) fluorescent signal. Statistical significance was determined by unpaired t-test. A nested scatterplot (bottom) representing quantification (n = 3) of an area of individual droplets from three independent experiments with BRCA1 BRCT, mGFP-p-hCTD, and Cy5-ITS1 RNA, shown in (C), with median area determined per dataset. Statistical significance was determined by nested t-test. Bar chart (bottom) depicts the proportion of droplets containing signals for mGFP-p-hCTD (green) and Cy5-ITS1 RNA (red).

Supplementary Figure 28:

A) LLPS assays with purified BARD1 BRCT, mGFP-p-hCTD, and Cy5-ITS1 RNA. BARD1 BRCT (at 10 µM) was mixed with phosphorylated pCTD (at 1.25 µM, 2.5 µM, and 5 µM, respectively), and ITS1 RNA (15 nM) in the presence of a crowding agent (10% PEG 8000) and $MgCl_2$ (at 3 mM). Representative images from three experiments are depicted as an overlay of differential interference contrast (DIC), GFP and Cy5 signal. Scale bars, 10 µm.

B) Bar chart (top) representing quantification (n = 3) of the number of droplets per frame from the LLPS experiments with BARD1 BRCT, mGFP-p-hCTD, and Cy5-ITS1 RNA, shown in (A), using the green (left) or red (right) fluorescent signal. Statistical significance was determined by unpaired t-test. A nested scatterplot (middle) representing quantification (n = 3) of an area of individual droplets from three independent experiments with BARD1 BRCT, mGFP-p-hCTD, and Cy5-ITS1 RNA, shown in (A), with median area determined per dataset. Statistical significance was determined by nested t-test. Bar chart (bottom) depicts the proportion of droplets containing signals for mGFP-p-hCTD (green) and Cy5-ITS1 RNA (red).

C) LLPS assays with purified BARD1 BRCT, mGFP-p-hCTD, and Cy5-ITS1 RNA. BARD1 BRCT (at 10 µM) was mixed with phosphorylated pCTD (at 2.5 µM), and ITS1 RNA (at 7.5 nM, 15 nM, and 30 nM, respectively) in the presence of a crowding agent (10% PEG 8000) and $MgCl_2$ (at 3 mM). Representative images from three experiments are depicted as an overlay of differential interference contrast (DIC), GFP and Cy5 signal. Scale bars, 10 µm.

D) Bar chart (top) representing quantification (n = 3) of the number of droplets per frame from the LLPS experiments with BARD1 BRCT, mGFP-p-hCTD, and Cy5-ITS1 RNA, shown in (C), using the green (left) or red (right) fluorescent signal. Statistical significance was determined by unpaired t-test. A nested scatterplot (bottom) representing quantification (n = 3) of an area of individual droplets from three independent experiments with BARD1 BRCT, mGFP-p-hCTD, and Cy5-ITS1 RNA, shown in (C), with median area determined per dataset. Statistical significance was determined by nested t-test. Bar chart (bottom) depicts the proportion of droplets containing signals for mGFP-p-hCTD (green) and Cy5-ITS1 RNA (red).

Supplementary Figure 29:

A) AlphaFold 3 model of the BRCA1 BRCT with the S5 pCTD shown in colours of pLDDT values. The regions predicted with high accuracy (pLDDT>90), are depicted in dark blue, good backbone predictions (pLDDT between 70 and 90) are depicted in light blue, regions predicted with low accuracy (pLDDT <70) are depicted in yellow and orange,

B) Overlay of the five best AlphaFold 3 models of the BRCA1 BRCT with the S5 pCTD.

C) AlphaFold 3 model of the BARD1 BRCT with the S5 pCTD shown in colours of pLDDT values. The regions predicted with high accuracy (pLDDT>90), are depicted in dark blue, good backbone predictions (pLDDT between 70 and 90) are depicted in light blue, regions predicted with low accuracy (pLDDT <70) are depicted in yellow and orange,

D) Overlay of the five best AlphaFold 3 models of the BARD1 BRCT with the S5 pCTD.

E) AlphaFold 3 model of the BRCA1 BRCT with RNA molecule (sequence ACGGAGCCCG) shown in colours of pLDDT values. The regions predicted with high accuracy (pLDDT>90), are depicted in dark blue, good backbone predictions (pLDDT between 70 and 90) are depicted in light blue, regions predicted with low accuracy (pLDDT <70) are depicted in yellow and orange,

F) Overlay of the five best AlphaFold 3 models of the BRCA1 BRCT with RNA molecule (sequence ACGGAGCCCG).

G) AlphaFold 3 model of the BARD1 BRCT with RNA molecule (sequence ACGGAGCCCG) shown in colours of pLDDT values. The regions predicted with high accuracy (pLDDT>90), are depicted in dark blue, good backbone predictions (pLDDT between 70 and 90) are depicted in light blue, regions predicted with low accuracy (pLDDT <70) are depicted in yellow and orange,

F) Overlay of the five best AlphaFold 3 models of the BARD1 BRCT with RNA molecule (sequence ACGGAGCCCG).

Supplementary Table 1: List of plasmids used in this study.

Name	Addgene #	Plasmid	Tags / Fusion proteins	Bacterial resistance
2AT	29665	pET LIC cloning vector	-	Ampicilin
2BT	29666	pET His6 TEV LIC cloning vector	His6-TEV (N-terminal on backbone)	Ampicilin
2CT-10	55209	pET His10 MBP Asn10 TEV LIC cloning vector	His10-MBP-N10-TEV (N-terminal on backbone)	Ampicilin
2Bc-T	37236	pET His6 LIC cloning vector	TEV-His6 (C-terminal on backbone)	Ampicilin
2E	29775	pET empty polycistronic destination vector	-	Kanamycin
438-A	55218	pFastBac cloning vector with BioBrick PolyPromoter LIC Subcloning	-	Ampicilin
438-B	55219	pFastBac His6 TEV cloning vector with BioBrick PolyPromoter LIC Subcloning	His6-TEV (N-terminal on backbone)	Ampicilin
H6-msfGFP	29725	pET Biotin His6 GFP LIC cloning vector	Biotin- His6-TEV (N terminal on backbone), GFP (C-terminal on backbone)	Kanamycin

Supplementary Table 2: List of oligonucleotides used in this study.

Name	Sequence	Use
T7F	TAATACGACTCACTATAGGG	Sequencing
T7R	GCTAGTTATTGCTCAGCGG	Sequencing
M13F	CCCAGTCACGACGTTGTAAAACG	Bacmid screening
M13R	AGCGGATAACAATTTCACACAGG	Bacmid screening
FB01	CCTATAACTATTCCGGATTATTCATACCGTC	Sequencing
FB02	CAGGTTCAGGGGGAGGTGTG	Sequencing
pRVK007	AAGAATTGTTACAAATCACC	Sequencing of BRCA1
pRVK008	TGGATAACACTAAATAGC	Sequencing of BRCA1
pRVK009	ATAAGCAATATGGAACTCG	Sequencing of BRCA1
pRVK010	GAACCAAATAAATGTGTG	Sequencing of BRCA1
pRVK011	AGAACATTCCAAGTACAG	Sequencing of BRCA1
pRVK012	CAATATACCTTCTCAGTC	Sequencing of BRCA1
pRVK013	AGAAAAGTAGTGAATACC	Sequencing of BRCA1
pRVK014	TACTTCCAATCCAATGCAATGCCGGATAATCGGCAG	Forward primer for cloning of BARD1 into 438B, v1 tag
pRVK015	TTATCCACTTCCAATGTTATTATTAGCTGTCAAGAGGAAG CA	Reverse primer for cloning of BARD1 into 438B, v1 tag
pRVK016	GAAAGTCAGATATGTTGTG	Sequencing of BARD1

pRVK017	GTGGAGATTTTGTTAAGC	Sequencing of BARD1
pRVK018	TACTTCCAATCCAATCGATGGACTACAAGGACGATGACGACAAGGGAAGTGGTATGGATTTATCTGCTCTTCGC	Forward primer for cloning of BRCA1 into 438A, vBac F tag, FLAG tag
pRVK019	TTATCCACTTCCAATGTTATTATCAGTAGTGGCTGTGGGG	Reverse primer for cloning of BRCA1 into 438A, v1 tag
pRVK032	TCTGTAGAAGTCTTTTGG	Sequencing of BRCA1, reverse
pRVK041	TACTTCCAATCCAATGCAGTCAACAAAAGAATGTCC	Forward primer for cloning of BRCA1 $^{1646\text{-}1859}$ into 2BT, v1 tag
pRVK042	TTATCCACTTCCAATGTTATTATCAGGGGATCTGGGGTAT	Reverse primer for cloning of BRCA1 $^{1646\text{-}1859}$ into 2BT, v1 tag
pRVK048	TACTTCCAATCCAATGCAGCTAGCCACTGCTCAGTA	Forward primer for cloning of BARD1 $^{554\text{-}777}$ into 2BT, v1 tag
pRVK064	CAAAAGAATGTCCATGGTGGTGTTTGGCCTGACCCCAGAAGAATTTATG	Forward primer for site-directed mutagenesis to introduce a S1655F substitution into BRCA1
pRVK065	CATAAATTCTTCTGGGGTCAGGCCAAACACCACCATGGACATTCTTTTG	Reverse primer for site-directed mutagenesis to introduce a S1655F substitution into BRCA1
pRVK066	GTTTGTGTGTGAACGGACACTGATGTATTTTCTAGGAATTGCGGGAGG	Forward primer for site-directed mutagenesis to introduce a K1702M substitution into BRCA1
pRVK067	CCTCCCGCAATTCCTAGAAAATACATCAGTGTCCGTTCACACACAAAC	Reverse primer for site-directed mutagenesis to introduce a K1702M substitution into BRCA1
pRVK068	GATGGACCTCTTGTACTTATAGGCTTTGGGCTGTCTTCAGAACAACAG	Forward primer for site-directed mutagenesis to introduce a S575F substitution into BARD1
pRVK069	CTGTTGTTCTGAAGACAGCCCAAAGCCTATAAGTACAAGAGGTCCATC	Reverse primer for site-directed mutagenesis to introduce a S575F substitution into BARD1
pRVK070	GGTGATGCAGTTCAAAGTACCTTGGCGTGTATGCTTGGGATTCTCAATGG	Forward primer for site-directed mutagenesis to introduce a K619M substitution into BARD1
pRVK071	CCATTGAGAATCCCAAGCATACACGCCAAGGTACTTTGAACTGCATCACC	Reverse primer for site-directed mutagenesis to introduce a K619M substitution into BARD1
pRVK072	TTTAAGAAGGAGATATAGTTCATGGACTACAAAGACGATGACGACAAGGAAAACCTGTACTTCCAATCCAATATGGATTTATCTGCTCTTCG	Forward primer for cloning of BRCA1 into 2BcT, FLAG tag, v3 tag
pRVK073	GGATTGGAAGTAGAGGTTCTCTCAGTAGTGGCTGTGGGG	Reverse primer for cloning of BRCA1 into 2BcT, v3 tag
pRVK074	TACTTCCAATCCAATCGATGGACTACAAAGACGATGAC	Forward primer for cloning of N-terminally FLAG-tagged sequences into 438 plasmids, vBAC F tag
pRVK075	TTATCCACTTCCAATGTTATTAATGGTGATGGTGATGGTG	Reverse primer for cloning of C-terminally His-tagged sequences into 438 plasmids, v1 tag
pRVK087	TACTTCCAATCCAATGCATATTCTGGTTCTTCAGAGAAA	Forward primer for cloning cloning of BRCA1$^{421\text{ - }1079}$ into 438C, v1 tag
pRVK092	TTATCCACTTCCAATGTTATTACAATTTTGGCCCTCTGTTTCT	Reverse primer for cloning of BRCA1 $^{421\text{ - }1079}$ into 438C, v1 tag
pRVK093	ACTGAGCCACAGATAATACAAGAGCATCCCCTCACAAATAAATTAAAGCGT	Forward primer for site-directed mutagenesis to introduce a R496H substitution into BRCA1
pRVK094	ACGCTTTAATTTATTTGTGAGGGGATGCTCTTGTATTATCTGTGGCTCAGT	Reverse primer for site-directed mutagenesis to introduce a R496H substitution into BRCA1

pRVK095	TACTGAGCCACAGATAATACAAGAGTGTCCCCTCACAAATAAATTAAAGC	Forward primer for site-directed mutagenesis to introduce a R496C substitution into BRCA1
pRVK096	GCTTTAATTTATTTGTGAGGGGACACTCTTGTATTATCTGTGGCTCAGTA	Reverse primer for site-directed mutagenesis to introduce a R496C substitution into BRCA1
pRVK097	CAGCAGTATAAGCAATATGGAACTCAAATTAAATATCCACAATTCAAAAG	Forward primer for site-directed mutagenesis to introduce a E597K substitution into BRCA1
pRVK098	CTTTTGAATTGTGGATATTTAATTTGAGTTCCATATTGCTTATACTGCTG	Reverse primer for site-directed mutagenesis to introduce a E597K substitution into BRCA1
pRVK099	GCTGAGGAGGAAGTCTTCTACCAGGAATATTCATGCGCTTGAACTAGTAG	Forward primer for site-directed mutagenesis to introduce a H619N substitution into BRCA1
pRVK100	CTACTAGTTCAAGCGCATGAATATTCCTGGTAGAAGACTTCCTCCTCAGC	Reverse primer for site-directed mutagenesis to introduce a H619N substitution into BRCA1
pRVK101	CAAGGGACTAATTCATGGTTGTTCCGAAGATAATAGAAATGACACAGAAG	Forward primer for site-directed mutagenesis to introduce a K820E substitution into BRCA1
pRVK102	CTTCTGTGTCATTTCTATTATCTTCGGAACAACCATGAATTAGTCCCTTG	Reverse primer for site-directed mutagenesis to introduce a K820E substitution into BRCA1
pRVK103	ATTGGGACATGAAGTTAACCACAGTTGGGAAACAAGCATAGAAATGGAAG	Forward primer for site-directed mutagenesis to introduce a R841W substitution into BRCA1
pRVK104	CTTCCATTTCTATGCTTGTTTCCCAACTGTGGTTAACTTCATGTCCCAAT	Reverse primer for site-directed mutagenesis to introduce a R841W substitution into BRCA1
pRVK110	CAGATGCTGAGTTTGTGTGTGAATGGACACTGAAATATTTTCTAGGAATTGC	Forward primer for site-directed mutagenesis to introduce a R1699W substitution into BRCA1
pRVK111	GCAATTCCTAGAAAATATTTCAGTGTCCATTCACACACAAACTCAGCATCTG	Reverse primer for site-directed mutagenesis to introduce a R1699W substitution into BRCA1
pRVK112	CAGATGCTGAGTTTGTGTGTGAACTGACACTGAAATATTTTCTAGGAATTGC	Forward primer for site-directed mutagenesis to introduce a R1699L substitution into BRCA1
pRVK113	GCAATTCCTAGAAAATATTTCAGTGTCAGTTCACACACAAACTCAGCATCTG	Reverse primer for site-directed mutagenesis to introduce a R1699L substitution into BRCA1
pRVK114	GCAGGTGGGGGCCAGATCCTCAGTGCAAAGCCCAAGCCAGACAGTGACGTG	Forward primer for site-directed mutagenesis to introduce a R705A substitution into BARD1
pRVK115	CACGTCACTGTCTGGCTTGGGCTTTGCACTGAGGATCTGGCCCCCACCTGC	Reverse primer for site-directed mutagenesis to introduce a R705A substitution into BARD1
pRVK128	CAGAACAACAGAAAATGCTCAGTAAGCTTGCAGTAATTCTTAAGGC	Forward primer for site-directed mutagenesis to introduce a E587K substitution into BARD1
pRVK129	GCCTTAAGAATTACTGCAAGCTTACTGAGCATTTTCTGTTGTTCTG	Reverse primer for site-directed mutagenesis to introduce a E587K substitution into BARD1
pRVK130	CCACGCAGAAGCAGGCTCAACAGAAAACAGCTGTTGCCAAAGCTGTTTG	Forward primer for site-directed mutagenesis to introduce a E665K substitution into BARD1
pRVK131	CAAACAGCTTTGGCAACAGCTGTTTTCTGTTGAGCCTGCTTCTGCGTGG	Reverse primer for site-directed mutagenesis to introduce a E665K substitution into BARD1
pRVK132	CAGTAGAAAGCCCAAGCCAGACCGTGACGTGACTCAGACCATCAATAC	Forward primer for site-directed mutagenesis to introduce a S711R substitution into BARD1

pRVK133	GTATTGATGGTCTGAGTCACGTCACGGTCTGGCTTGGGCT TTCTACTG	Reverse primer for site-directed mutagenesis to introduce a S711R substitution into BARD1
pRVK134	CAGAGAGGGTTCGGCAGGGCAACGTCTGGAAGGCTCCTT CGAGCTG	Forward primer for site-directed mutagenesis to introduce a K754N substitution into BARD1
pRVK135	CAGCTCGAAGGAGCCTTCCAGACGTTGCCCTGCCGAACCC TCTCTG	Reverse primer for site-directed mutagenesis to introduce a K754N substitution into BARD1
pRVK139	TTTAAGAAGGAGATATAGATCATGGGAGGCCTGAACGAT	Forward primer for cloning of Avi-tagged sequences into 2AT, v2 tag
pRVK140	TTATGGAGTTGGGATCTTATTAGCTGTCAAGAGGAAGCAA	Reverse primer for cloning of BARD1 into 2AT, v2 tag
pRVK141	TACTTCCAATCCAATGCAAAGGATAACACCGTGCCA	Forward primer for cloning of BirA into 2CT10, v1 tag
pRVK142	TTATCCACTTCCAATGTTATTATTTTCTGCACTACGCAG	Reverse primer for cloning of BirA into 2CT10, v1 tag
pRVK159	GTTGCTATGGGCCCTTCACCAACAAGCCCACAGATCAACT GGAATGGATG	Forward primer for site-directed mutagenesis to introduce a M1775K substitution into BRCA1
pRVK160	CATCCATTCCAGTTGATCTGTGGGCTTGTTGGTGAAGGGC CCATAGCAAC	Reverse primer for site-directed mutagenesis to introduce a M1775K substitution into BRCA1
pRVK161	GTTGCTATGGGCCCTTCACCAACCACCCCACAGATCAACT GGAATGGATG	Forward primer for site-directed mutagenesis to introduce a M1775H substitution into BRCA1
pRVK162	CATCCATTCCAGTTGATCTGTGGGGTGGTTGGTGAAGGGC CCATAGCAAC	Reverse primer for site-directed mutagenesis to introduce a M1775H substitution into BRCA1
pRVK163	CAGATGCTGAGTTTGTGTGTGAATCGACACTGAAATATTT TCTAGGAATTGC	Forward primer for site-directed mutagenesis to introduce a R1699S substitution into BRCA1
pRVK164	GCAATTCCTAGAAAATATTTCAGTGTCGATTCACACACAA ACTCAGCATCTG	Reverse primer for site-directed mutagenesis to introduce a R1699S substitution into BRCA1
pRMS217	GACGCTGCCGAATTCTACCAGTGCCTTGCTA GGACATCTTTGCCCACCTGCAGGTTCACCC	61-mer DNA primer fluorescently labelled on 5’ end with Cy3 used to create R-loop/D-loop/bubble substrates for EMSAs
pRMS219	GGGTGAACCTGCAGGTGGGCGGCTGCTCAT CGTAGGTTAGTTGGTAGAATTCGGCAGCGTC	61-mer DNA primer used to create R-loop/D-loop/bubble substrates for EMSAs
pRMS223	AAAGAUGUCCUAGCAAGGCAC	21-mer RNA primer used to create an R-loop substrate for EMSAs
pRMS391	GGGTGAACCTGCAGGTGGGCAAAGATGTCC TAGCAAGGCACTGGTAGAATTCGGCAGCGT C	61-mer DNA primer used to create a double stranded DNA substrate for EMSA
pRMS433	CACTTTAACTAATCTAATTACTAAAGAGACTACTCATGTT GTTATG	Forward primer for site-directed mutagenesis to introduce a E1682K substitution into BRCA1
pRMS434	CATAACAACATGAGTAGTCTCTTTAGTAATTAGATTAGTT AAAGTG	Reverse primer for site-directed mutagenesis to introduce a E1682K substitution into BRCA1
pRMS435	CCAAGGTCCAAAGCGAGCAAGAAAATCCCAGGACAGAAA GATCTTC	Forward primer for site-directed mutagenesis to introduce a E1754K substitution into BRCA1
pRMS436	GAAGATCTTTCTGTCCTGGGATTTTCTTGCTCGCTTTGGAC CTTGG	Reverse primer for site-directed mutagenesis to introduce a E1754K substitution into BRCA1
pRMS437	CCAAGGTCCAAAGCGAGCAAGAGAATACCAGGACAGAAA GATCTTCAG	Forward primer for site-directed mutagenesis to introduce a S1755Y substitution into BRCA1

pRMS438	CTGAAGATCTTTCTGTCCTGGTATTCTCTTGCTCGCTTTGG ACCTTGG	Reverse primer for site-directed mutagenesis to introduce a S1755Y substitution into BRCA1
pRMS679	TTGTCTTCTCATAAATTTAATCCCCGTACGCTTATACTCCT TTAACTACCCAGTCTTCCCG	61-mer DNA primer fluorescently labelled on 5' end with Cy3 used as a single-stranded DNA for EMSAs

Supplementary Table 3: List of recombinant plasmids used in this study.

Name of the plasmid	Backbone	ORF	Boundaries (AA)	Purpose
pVK1.1	438B	BARD1	1-777 (full-length)	Transfer vector / cloning intermediate
pVK1.2	2BT	BRCA1	1646-1859	CTD binding assays, LLPS experiments
pVK1.3	2BT	BARD1	554-777	CTD binding assays, LLPS experiments
pVK1.4	2BT	BRCA1 S1655F	1646-1859	Transfer vector / cloning intermediate
pVK1.5	2BT	BRCA1 K1702M	1646-1859	Transfer vector / cloning intermediate
pVK1.6	2BT	BARD1 S575F	554-777	Transfer vector / cloning intermediate
pVK1.7	2BT	BARD1 K619A	554-777	Transfer vector / cloning intermediate
pVK1.8	2BT	BRCA1 S1655F, K1702M	1646-1859	CTD binding assays, LLPS experiments
pVK1.9	2BT	BARD1 S575F, K619A	554-777	CTD binding assays, LLPS experiments
pVK1.10	2BT	BRCA1 E1682K	1646-1859	CTD binding assays, LLPS experiments
pVK1.11	2BT	BRCA1 E1754K	1646-1859	CTD binding assays, LLPS experiments
pVK1.12	2BT	BRCA1 S1755Y	1646-1859	CTD binding assays, LLPS experiments
pVK1.13	2BcT	FLAG-BRCA1	1-1859 (full-length)	Transfer vector / cloning intermediate
pVK1.14	438A	FLAG-BRCA1-His	1-1859 (full-length)	Transfer vector / cloning intermediate
pVK1.15	438A	FLAG-BRCA1-His, His-BARD1	BRCA1 1-1859 (full-length), BARD1 1-777 (full-length)	Structural studies, biochemical characterization
pVK1.16	438C	BRCA1	421-1079	DNA binding assays
pVK1.17	438C	BRCA1 R496H	421-1079	DNA binding assays
pVK1.18	438C	BRCA1 R496C	421-1079	DNA binding assays
pVK1.19	438C	BRCA1 E597K	421-1079	DNA binding assays
pVK1.20	438C	BRCA1 H619N	421-1079	DNA binding assays
pVK1.21	438C	BRCA1 R841W	421-1079	DNA binding assays
pVK1.22	2BT	BRCA1 R1699W	1646-1859	CTD binding assays
pVK1.23	2BT	BRCA1 R1699L	1646-1859	CTD binding assays
pVK1.24	2BT	BARD1 R705A	554-777	CTD binding assays
pVK1.25	2BT	BARD1 E587K	554-777	CTD binding assays, LLPS experiments
pVK1.26	2BT	BARD1 E665K	554-777	CTD binding assays, LLPS experiments

pVK1.27	2BT	BARD1 S711R	554-777	CTD binding assays, LLPS experiments
pVK1.28	2BT	BARD1 K754N	554-777	CTD binding assays, LLPS experiments
pVK1.29	H6-msfGFP	BARD1	554-777	Transfer vector / cloning intermediate
pVK1.30	H6-msfGFP	BARD1 S575F, K619A	554-777	Transfer vector / cloning intermediate
pVK1.31	H6-msfGFP	BARD1 R705A	554-777	Transfer vector / cloning intermediate
pVK1.32	2AT	Avi-His-BARD1	554-777	CTD binding assays
pVK1.33	2AT	Avi-His-BARD1 S575F, K619A	554-777	CTD binding assays
pVK1.34	2AT	Avi-His-BARD1 R705A	554-777	CTD binding assays
pVK1.35	2CT10	BirA	1-321 (full-length)	Transfer vector / cloning intermediate
pVK1.36	H6-msfGFP	BRCA1	1646-1859	Transfer vector / cloning intermediate
pVK1.37	H6-msfGFP	BRCA1 S1655F, K1702M	1646-1859	Transfer vector / cloning intermediate
pVK1.38	H6-msfGFP	BRCA1 R1699L	1646-1859	Transfer vector / cloning intermediate
pVK1.39	H6-msfGFP	BRCA1 E1754K	1646-1859	Transfer vector / cloning intermediate
pVK1.40	2AT	Avi-His-BRCA1	1646-1859	CTD binding assays
pVK1.41	2AT	Avi-His-BRCA1 S1655F, K1702M	1646-1859	CTD binding assays
pVK1.42	2AT	Avi-His-BRCA1 R1699L	1646-1859	CTD binding assays
pVK1.43	2AT	Avi-His-BRCA1 E1754K	1646-1859	CTD binding assays
pVK1.44	2AT	Avi-His-BARD1 E665K	554-777	CTD binding assays
pVK1.45	2AT	Avi-His-BARD1 K754N	554-777	CTD binding assays
pVK1.46	2BT	BRCA1 M1775K	1646-1859	CTD binding assays
pVK1.47	2BT	BRCA1 M1775H	1646-1859	CTD binding assays
pVK1.48	2AT	Avi-His-BRCA1 M1775H	1646-1859	CTD binding assays
pVK1.49	2BT	BRCA1 R1699S	1646-1859	CTD binding assays
pVK1.50	2AT	Avi-His-BRCA1 R1699S	1646-1859	CTD binding assays
pVK1.51	438A	FLAG-BRCA1-His, His-BARD1 S575F, K619A	BRCA1 1-1859 (full-length), BARD1 1-777 (full-length)	CTD binding assays
pVK1.52	438A	FLAG-BRCA1-His S1655F, K1702M, His-BARD1	BRCA1 1-1859 (full-length), BARD1 1-777 (full-length)	CTD binding assays
pVK1.53	438A	FLAG-BRCA1-His S1655F, K1702M, His-BARD1 S575F, K619A	BRCA1 1-1859 (full-length), BARD1 1-777 (full-length)	CTD binding assays
pVK1.54	2BT	BARD1 H686M	554-777	CTD binding assays
pVK1.55	2AT	Avi-His-BARD1 H686M	554-777	CTD binding assays

Supplementary Table 4: Data collection and refinement statistics. Data in parentheses for highest resolution shell.

Data collection	
Complex	BRCA1/2xCTD
PDB ID	9QPX
Beamline, diffraction source	P13, PETRA III
Wavelength (Å)	0.9763
Space group	$C222_1$
a, b, c (Å)	112.83, 134.76, 180.38
α, β, γ (°)	90.00, 90.00, 90.00
No. of monomers in asymmetric unit	4
Resolution range (Å)	49.37–3.00 (2.85–2.70)
Total no. of reflections	382 280 (56 707)
No. of unique reflections	27 850 (4 024)
Completeness (%)	99.8 (99.9)
Redundancy	13.7 (14.1)
$\langle I/\sigma(I) \rangle$	7.2 (1.0)
R_{merge} (%)	0.289 (3.230)
CC(1/2)	0.996 (0.505)
Wilson B (Å^2)	76.3
Refinement statistics	
No. of protein amino acids	845
No. of protein atoms	6609
No. of peptide-ligand atoms	210
Resolution limits	49.42–3.00 (3.08–3.00)
No. of reflections in working set	26 437 (1938)
No. of reflections in test set	1386 (89)
Final R_{cryst} (%)	0.255 (0.498)
Final R_{free} (%)	0.312 (0.544)
Mean B factor (Å^2)	103.0
R.m.s. deviations	
Bonds (Å)	0.004
Angles (°)	1.257
Planar groups (Å)	0.005
Chiral volumes (Å^3)	0.064
Ramachandran plot	
Most favoured (%)	93.2
Allowed (%)	6.8

Supplementary figure 1 **Klapstova *et al.*,**

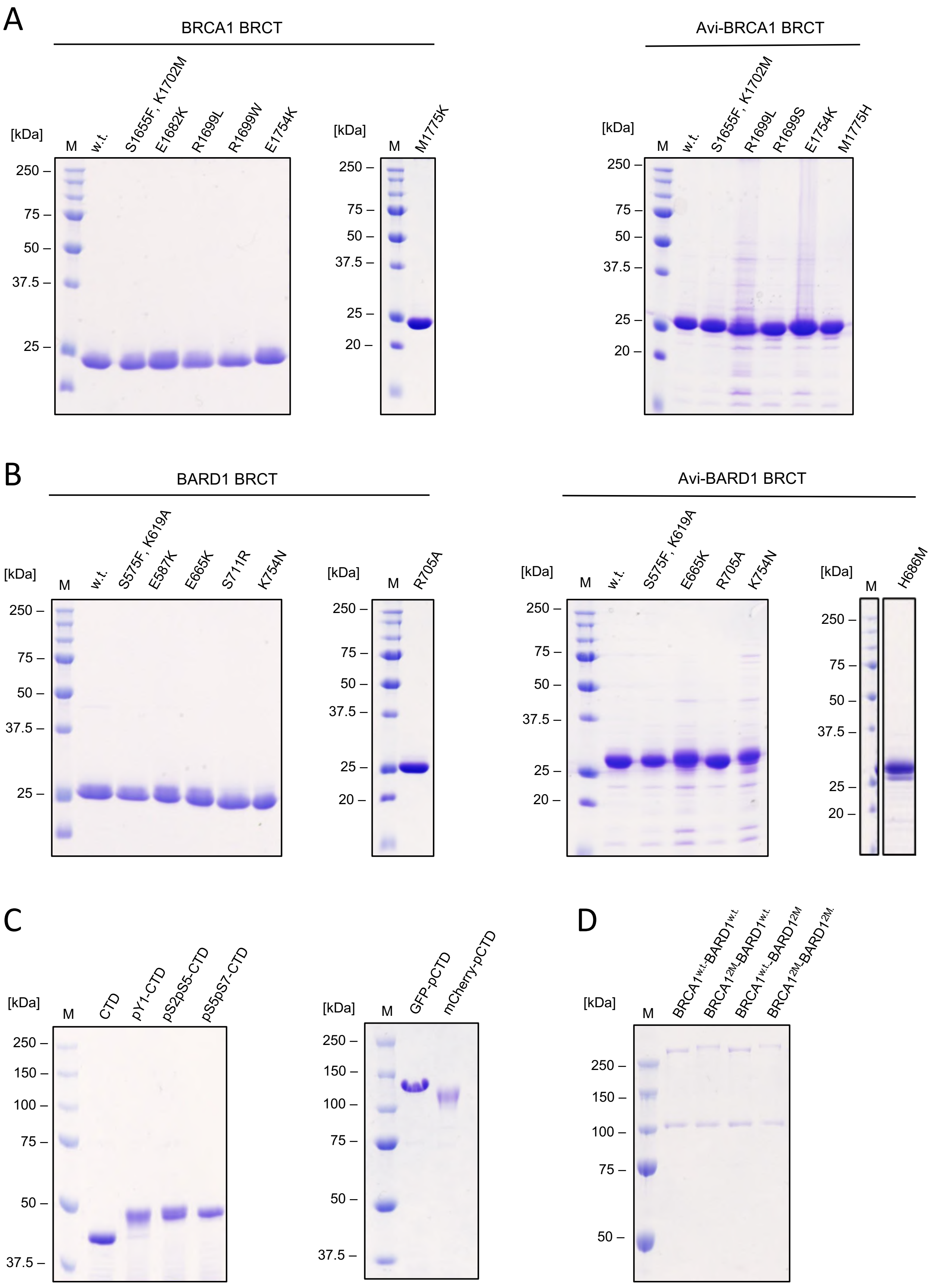

Supplementary figure 2 **Klapstova *et al.*,**

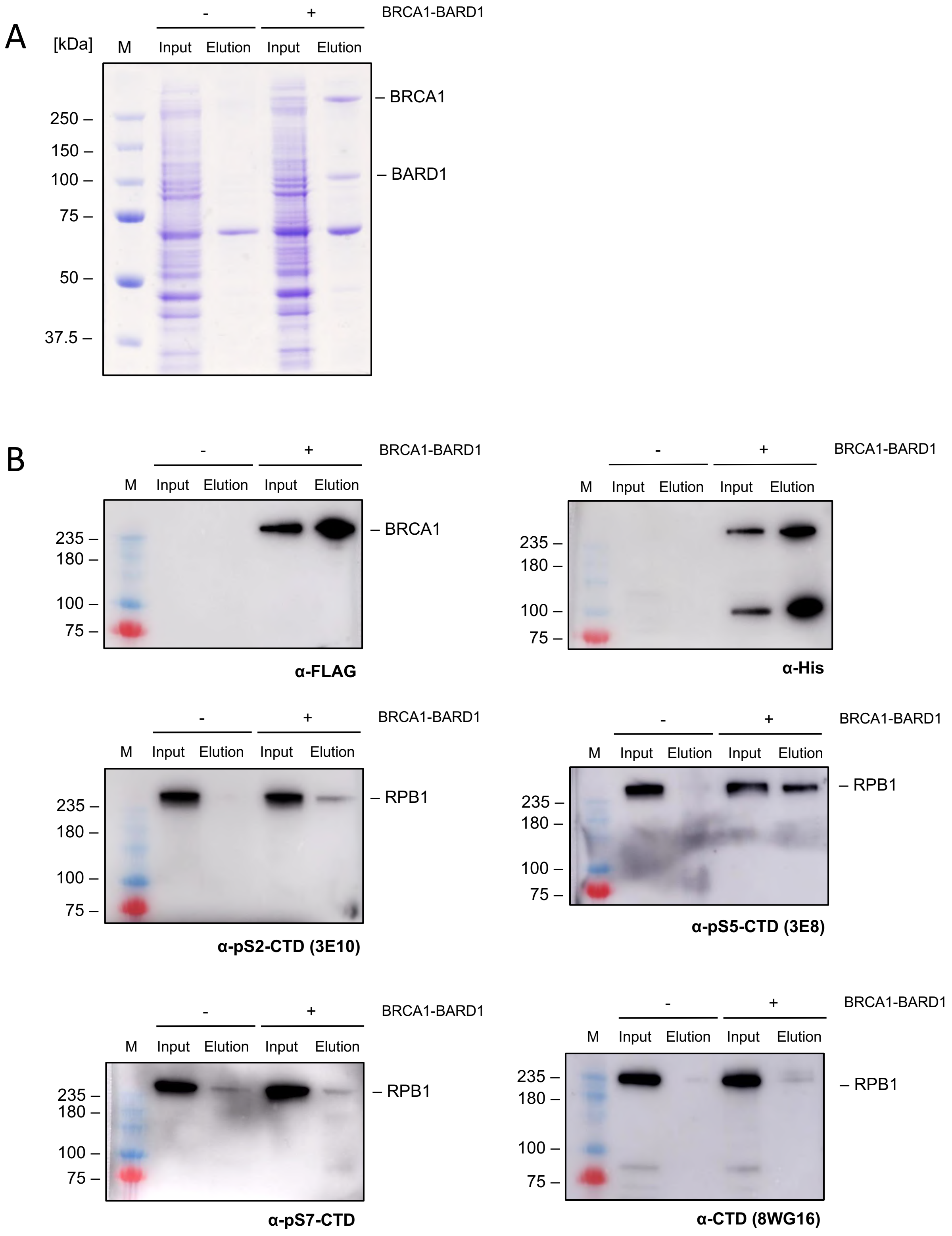

Supplementary figure 3 **Klapstova *et al.*,**

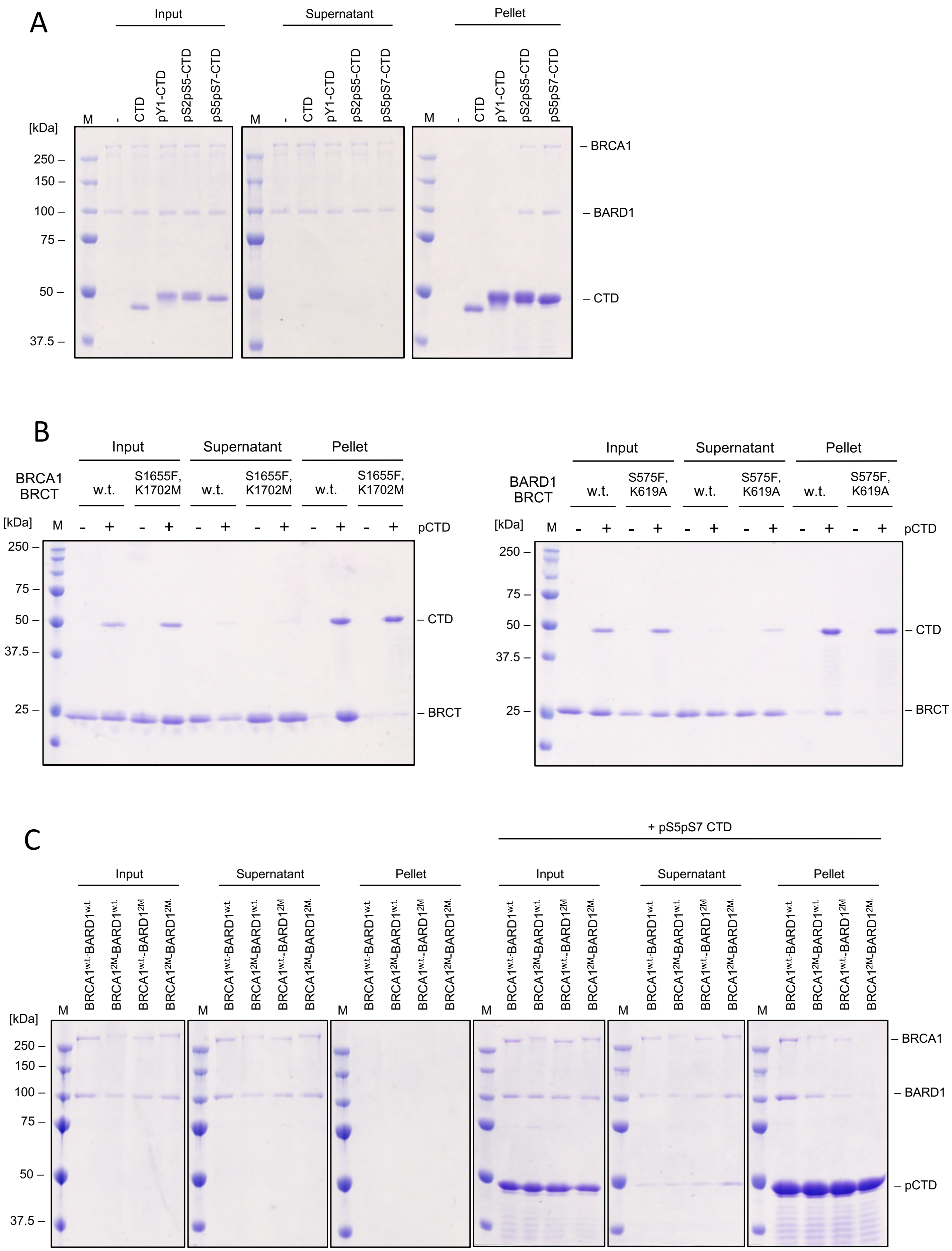

Supplementary figure 4

Klapstova *et al.*,

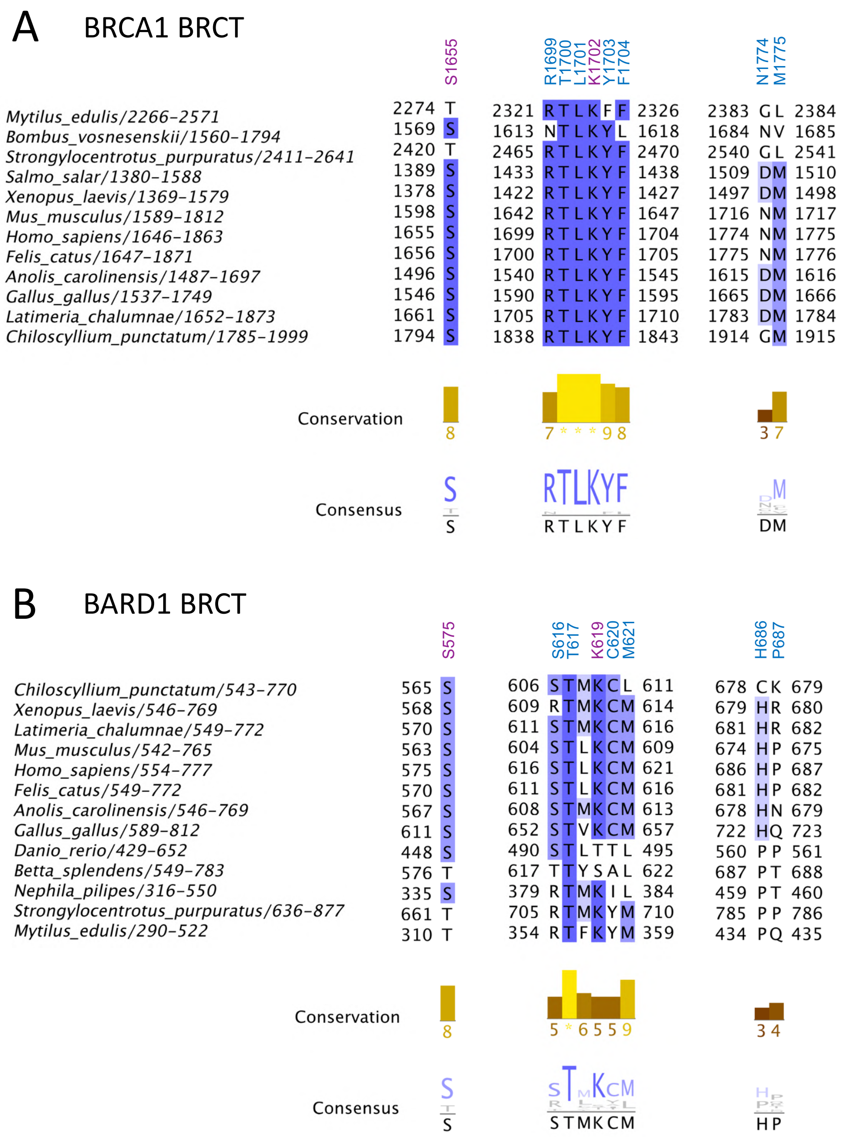

Supplementary figure 5 **Klapstova *et al.*,**

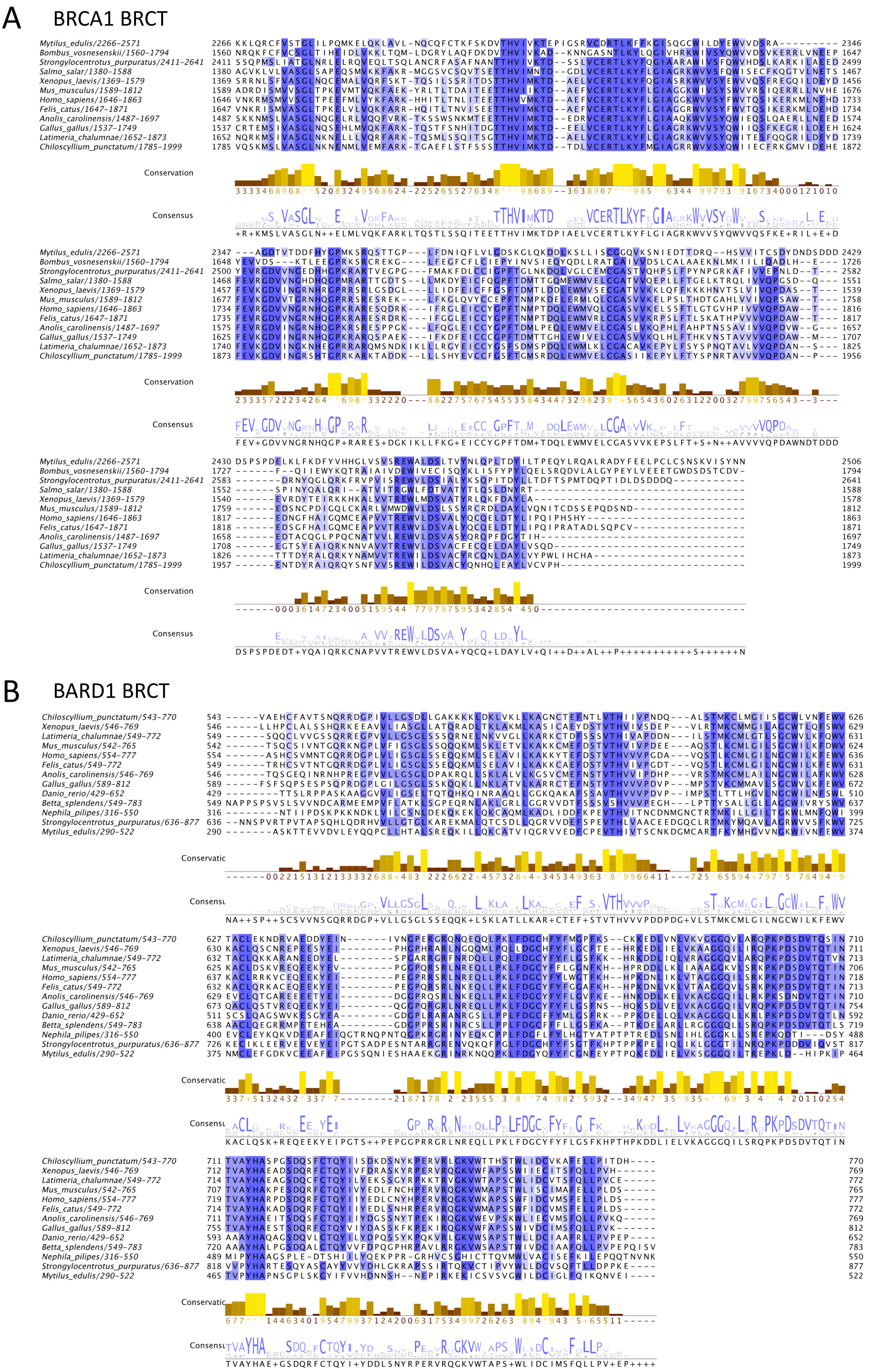

Supplementary figure 6 Klapstova *et al.*,

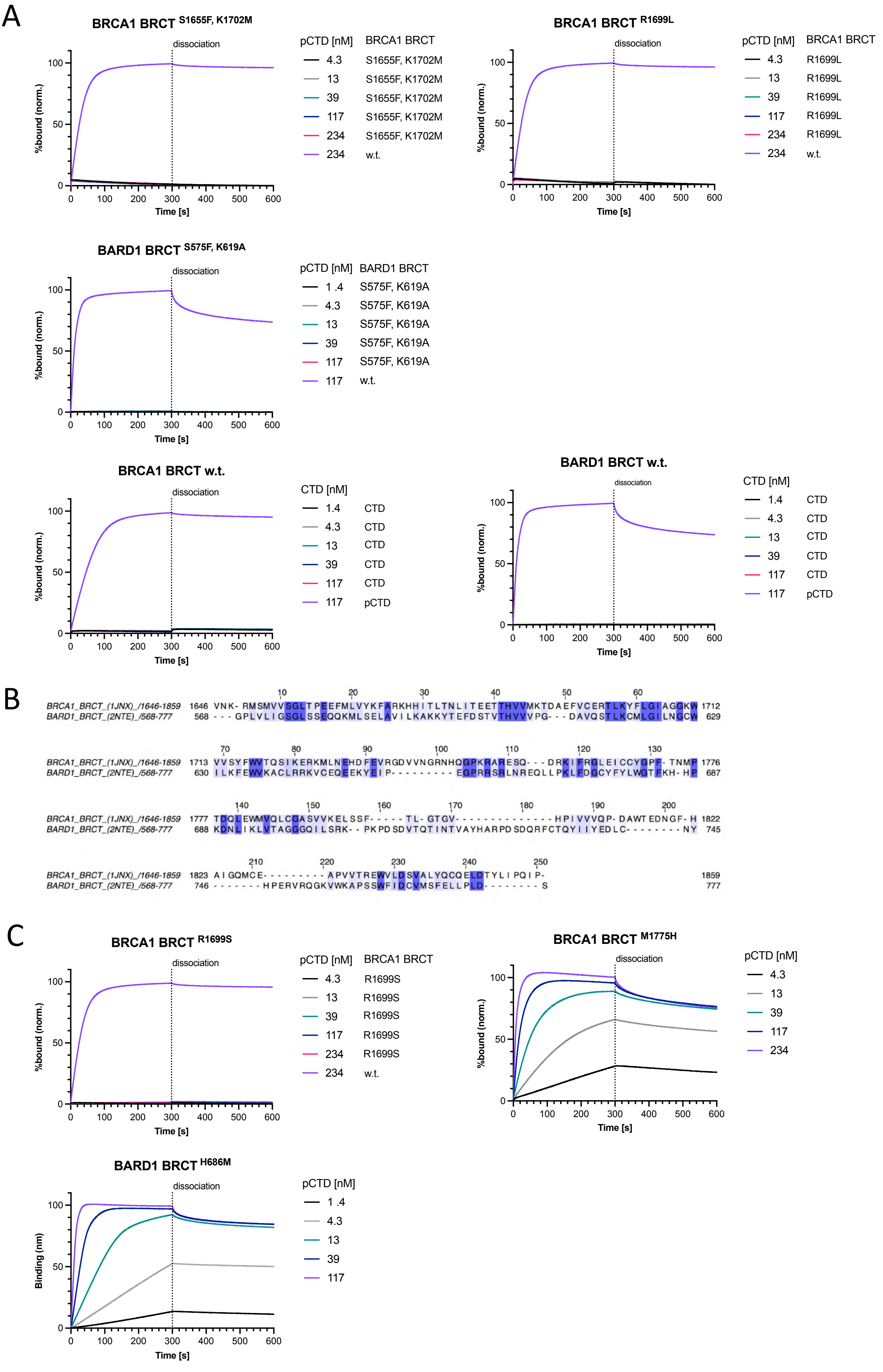

Supplementary figure 7 **Klapstova *et al.*,**

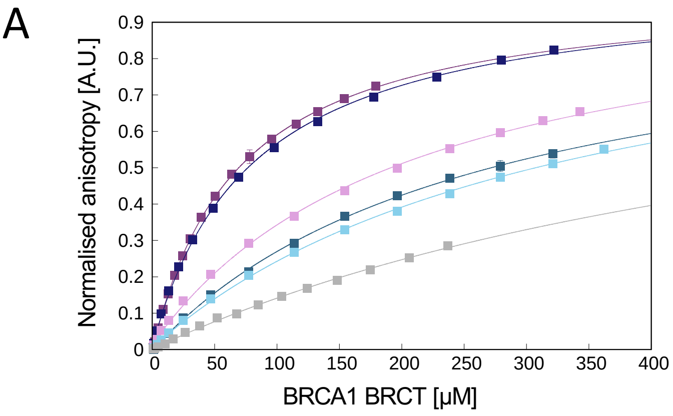

	CTD peptide	K_D [μM]
■	pS2pS5pS7	68.2 ± 0.9
■	pS5	78.4 ± 2.7
■	pS2pS5	202.6 ± 14.3
■	pS7	270.2 ± 12.2
■	pS2	314.7 ± 16.6
■	CTD	618.7 ± 93.1

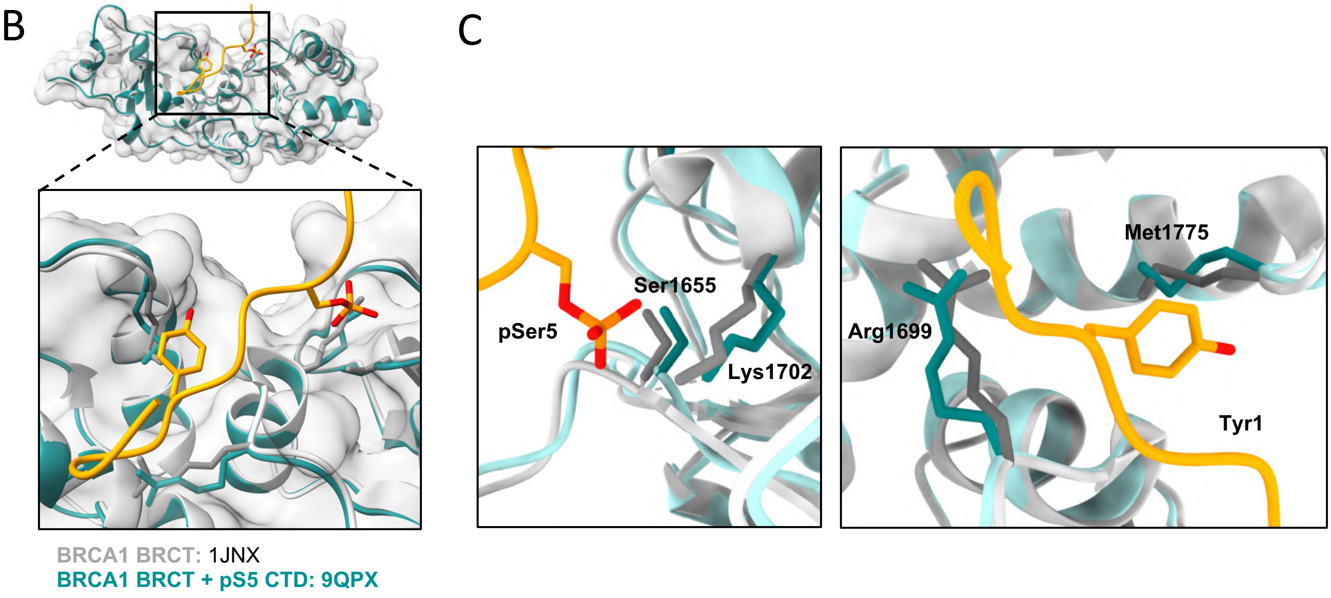

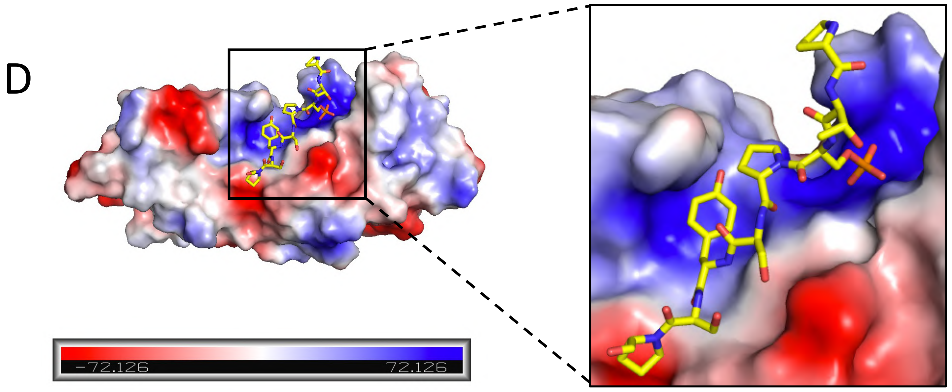

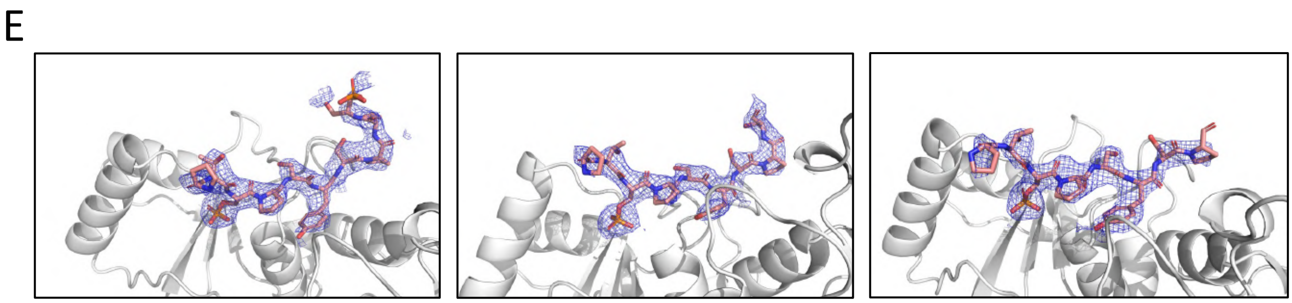

Supplementary figure 8 Klapstova *et al.*,

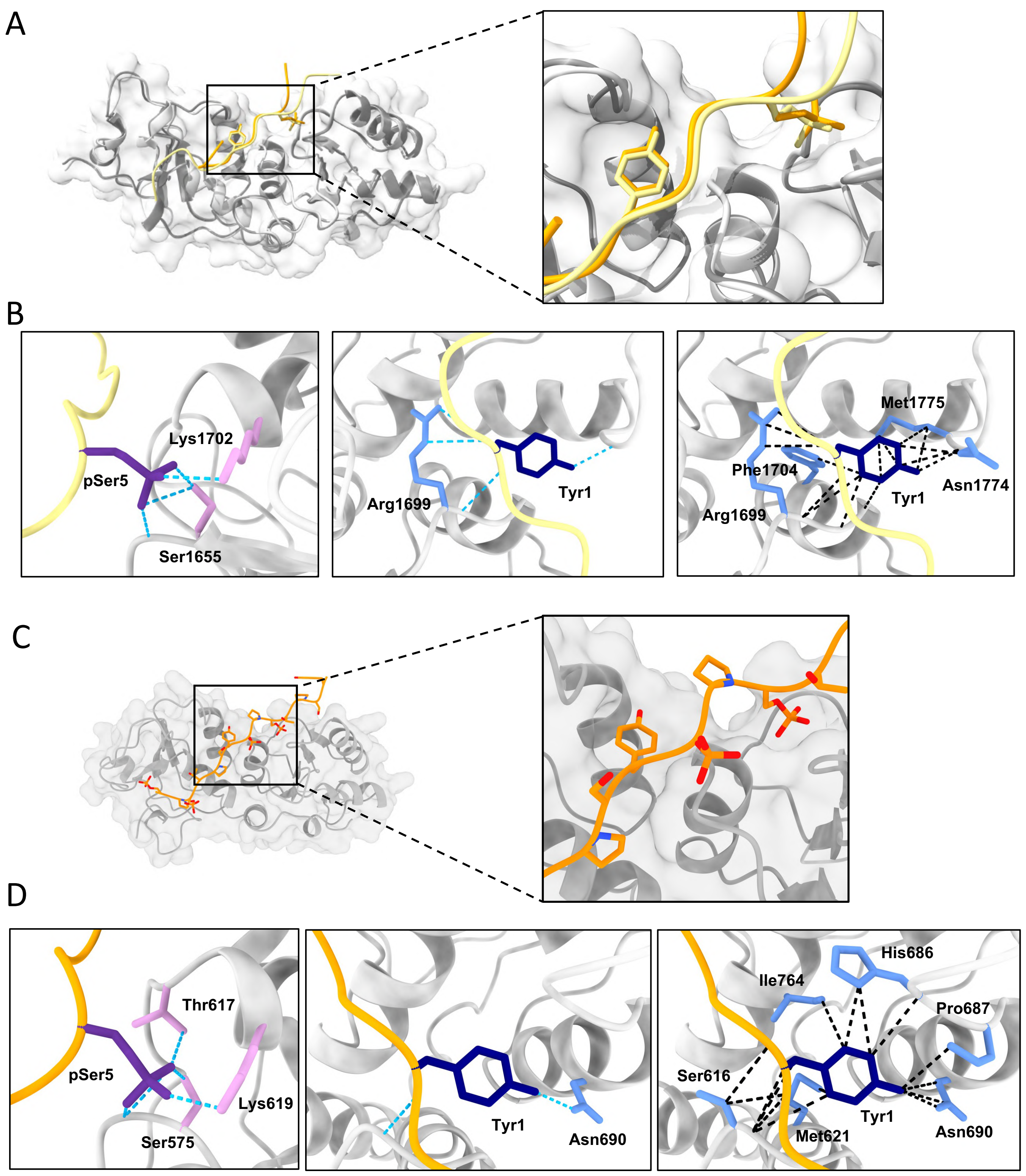

Supplementary figure 9

Klapstova *et al.*,

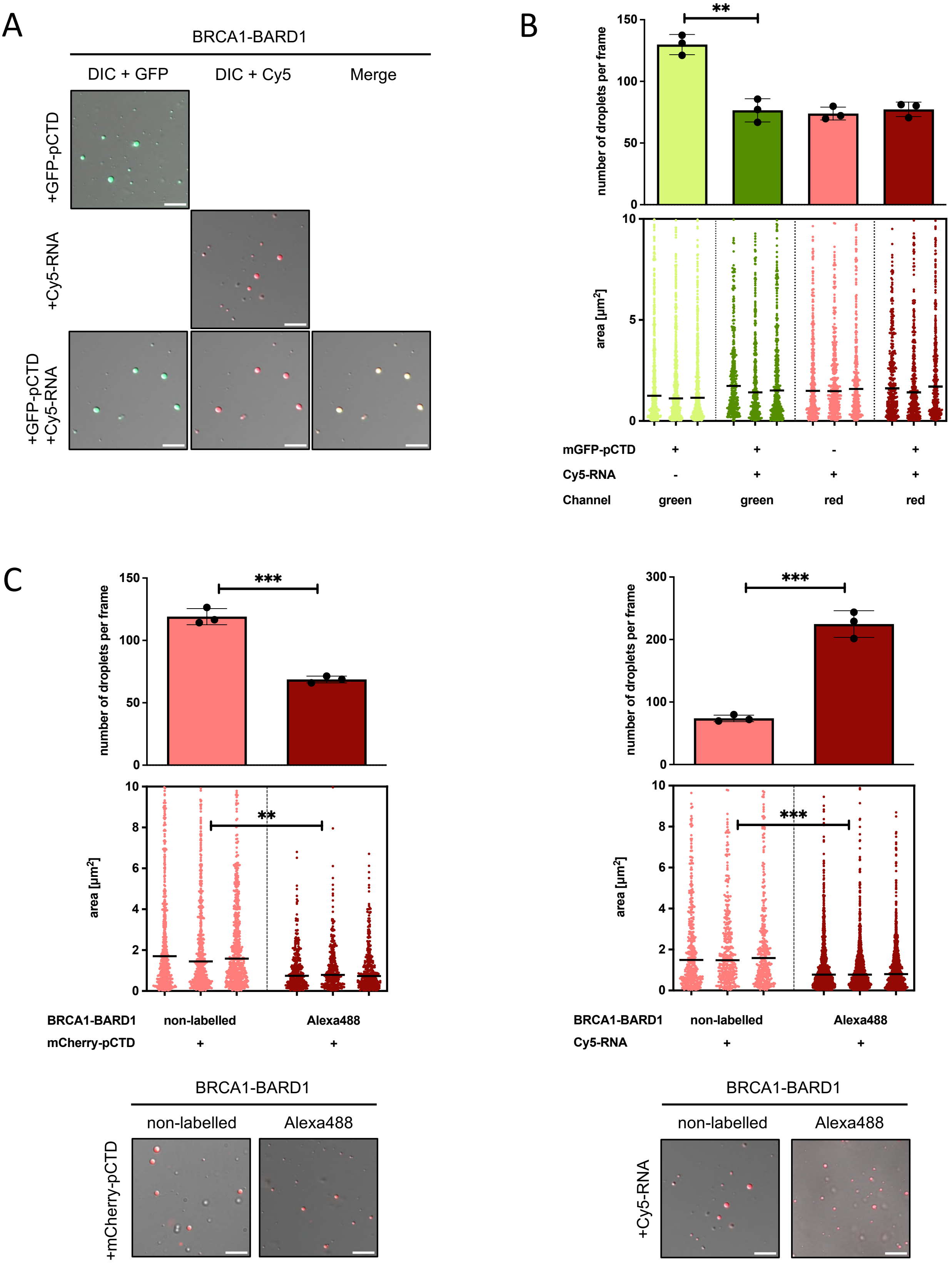

Supplementary figure 10 Klapstova *et al.*,

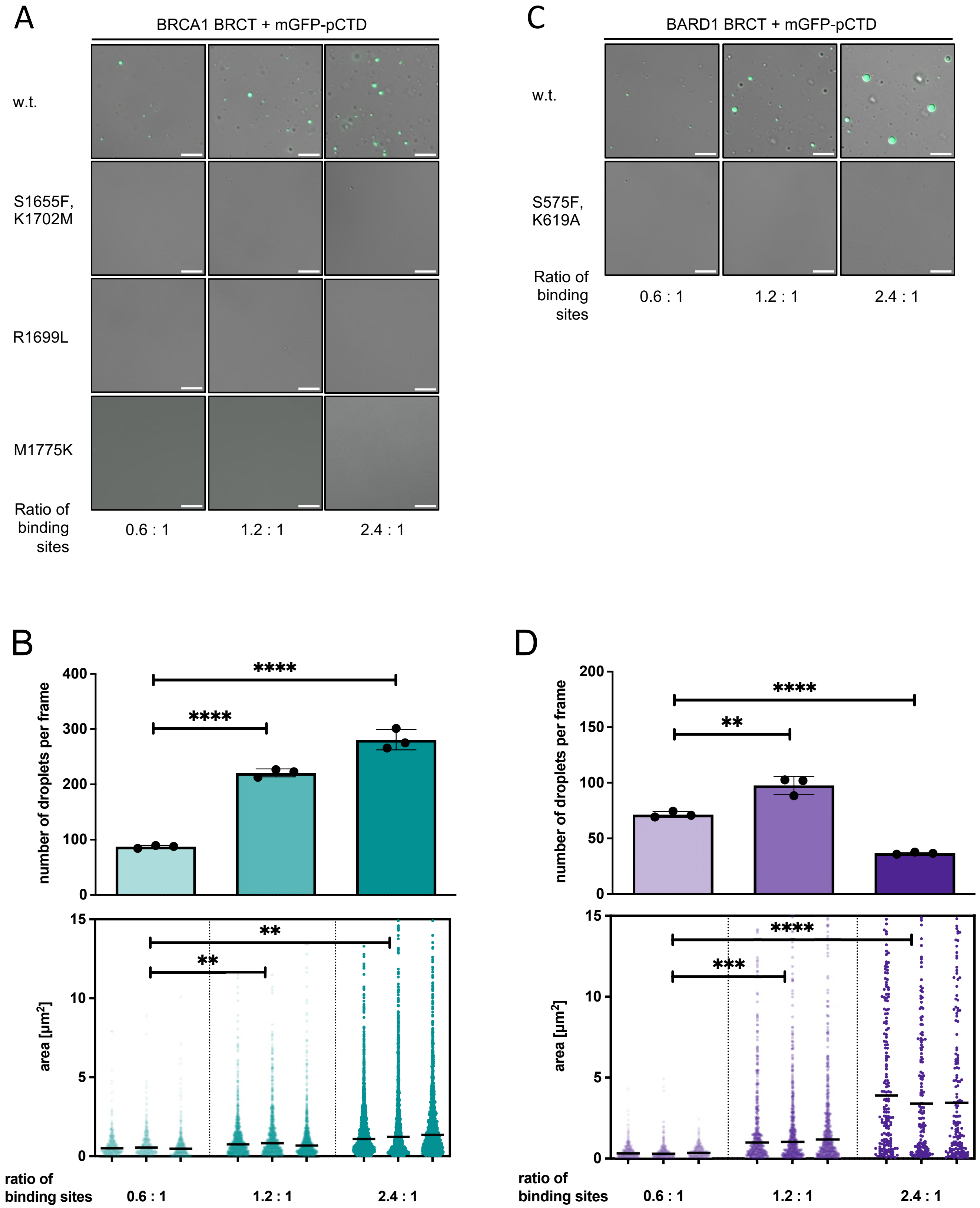

Supplementary figure 11 **Klapstova *et al.*,**

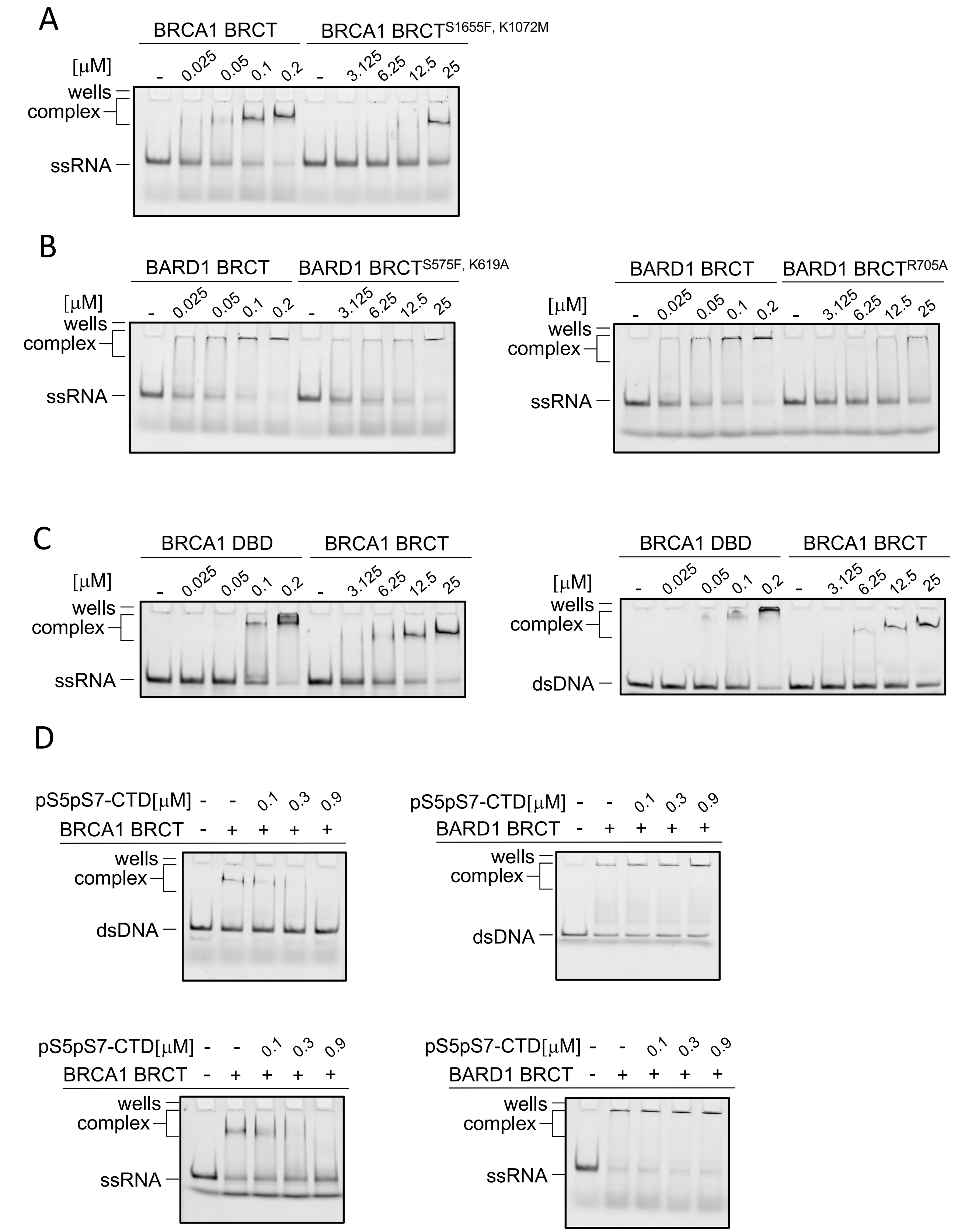

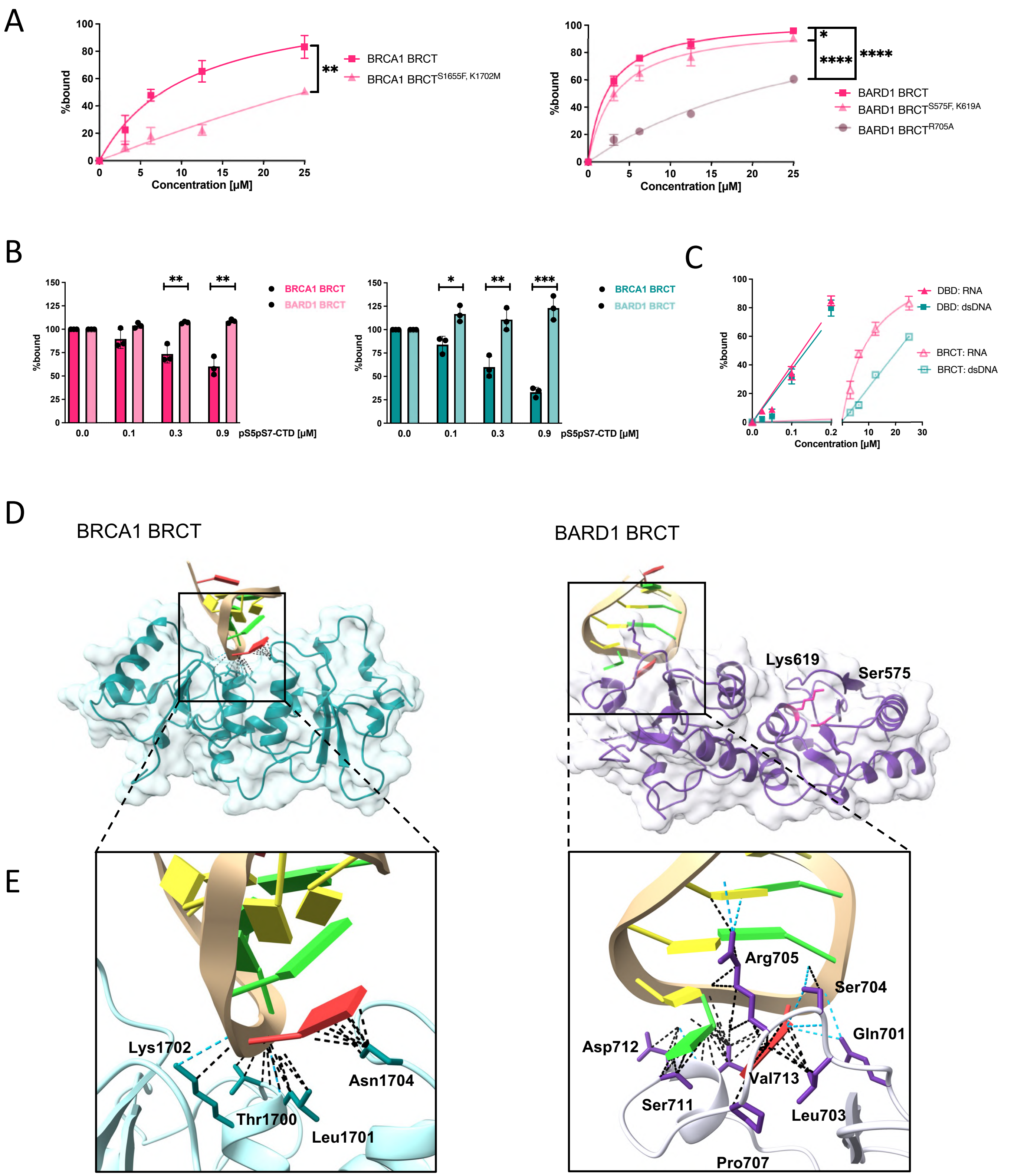
A
%bound
Concentration [µM]
**
BRCA1 BRCT
BRCA1 BRCT S1655F, K1702M
*
****
****
BARD1 BRCT
BARD1 BRCT S575F, K619A
BARD1 BRCT R705A
B
%bound
pS5pS7-CTD [µM]
BRCA1 BRCT
BARD1 BRCT
*
**
***
C
DBD: RNA
DBD: dsDNA
BRCT: RNA
BRCT: dsDNA
D
BRCA1 BRCT
BARD1 BRCT
Lys619
Ser575
E
Lys1702
Asn1704
Thr1700
Leu1701
Arg705
Ser704
Gln701
Asp712
Val713
Ser711
Leu703
Pro707

Supplementary figure 13 **Klapstova *et al.*,**

A

BRCA1 BRCT

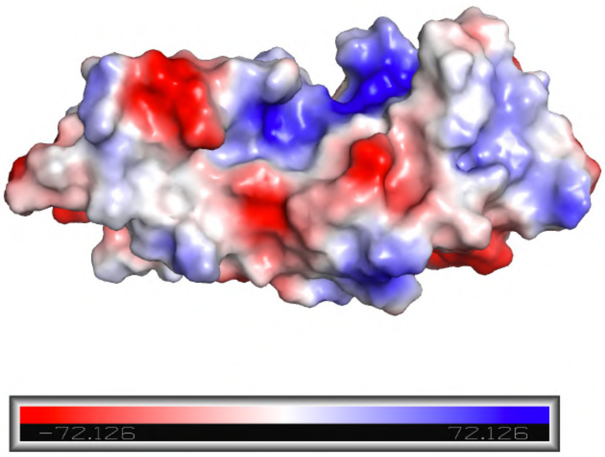

BARD1 BRCT

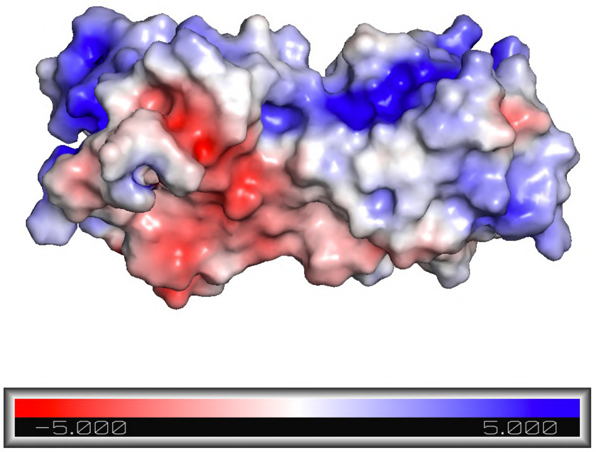

B

BARD1 BRCT

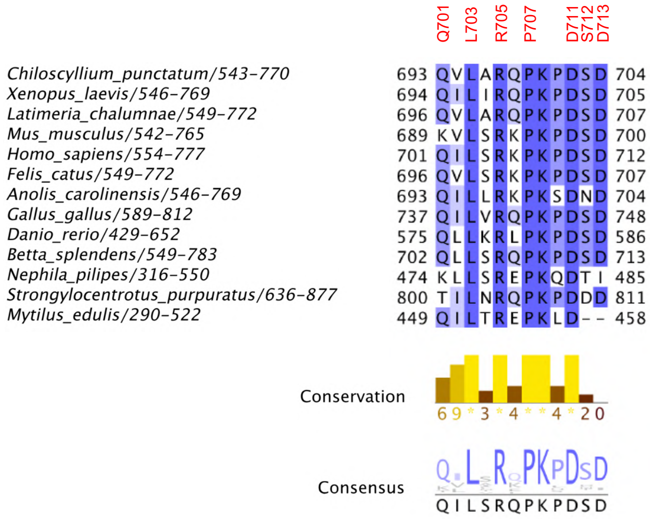

Supplementary figure 14 Klapstova *et al.*,

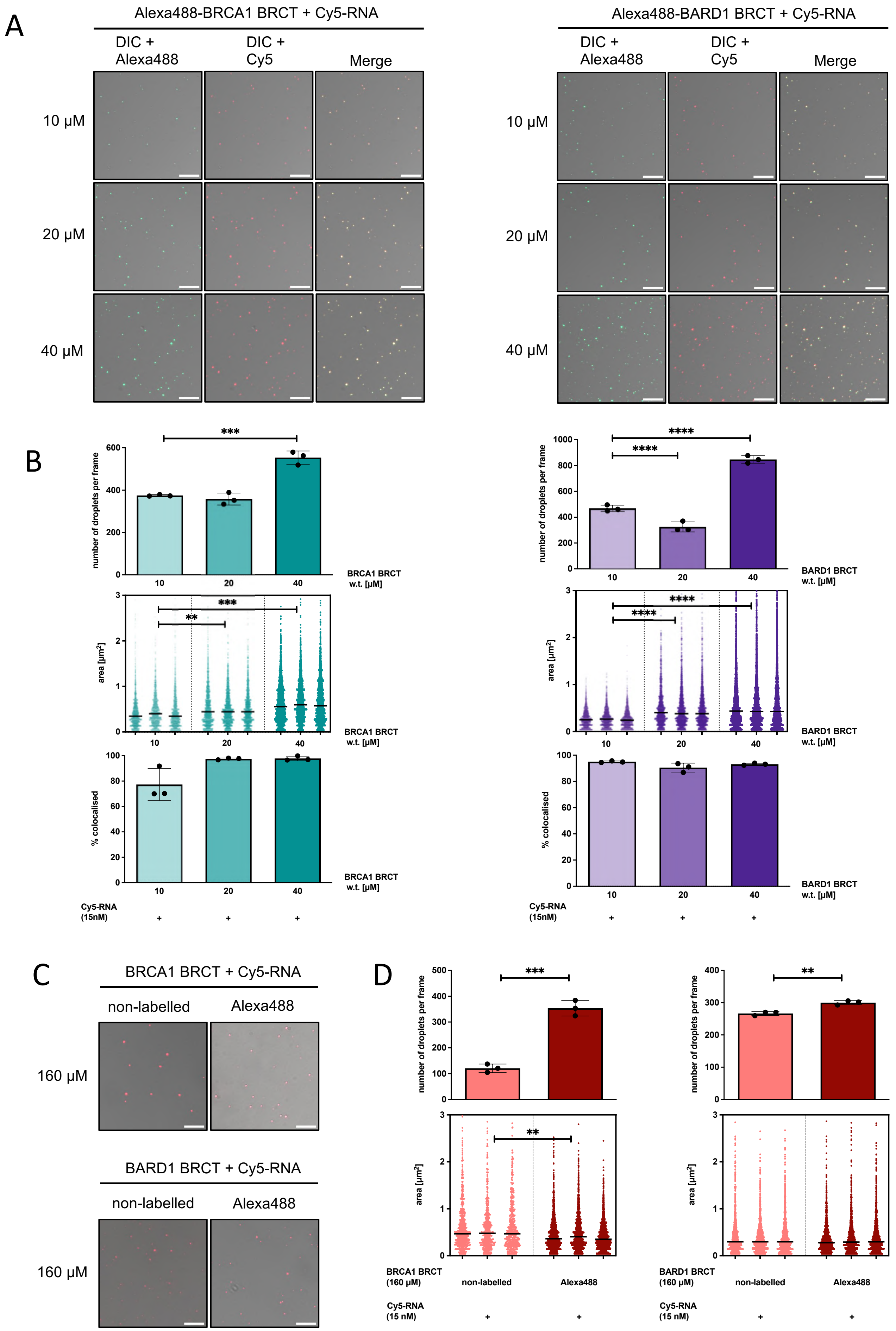

Supplementary figure 15 **Klapstova *et al.*,**

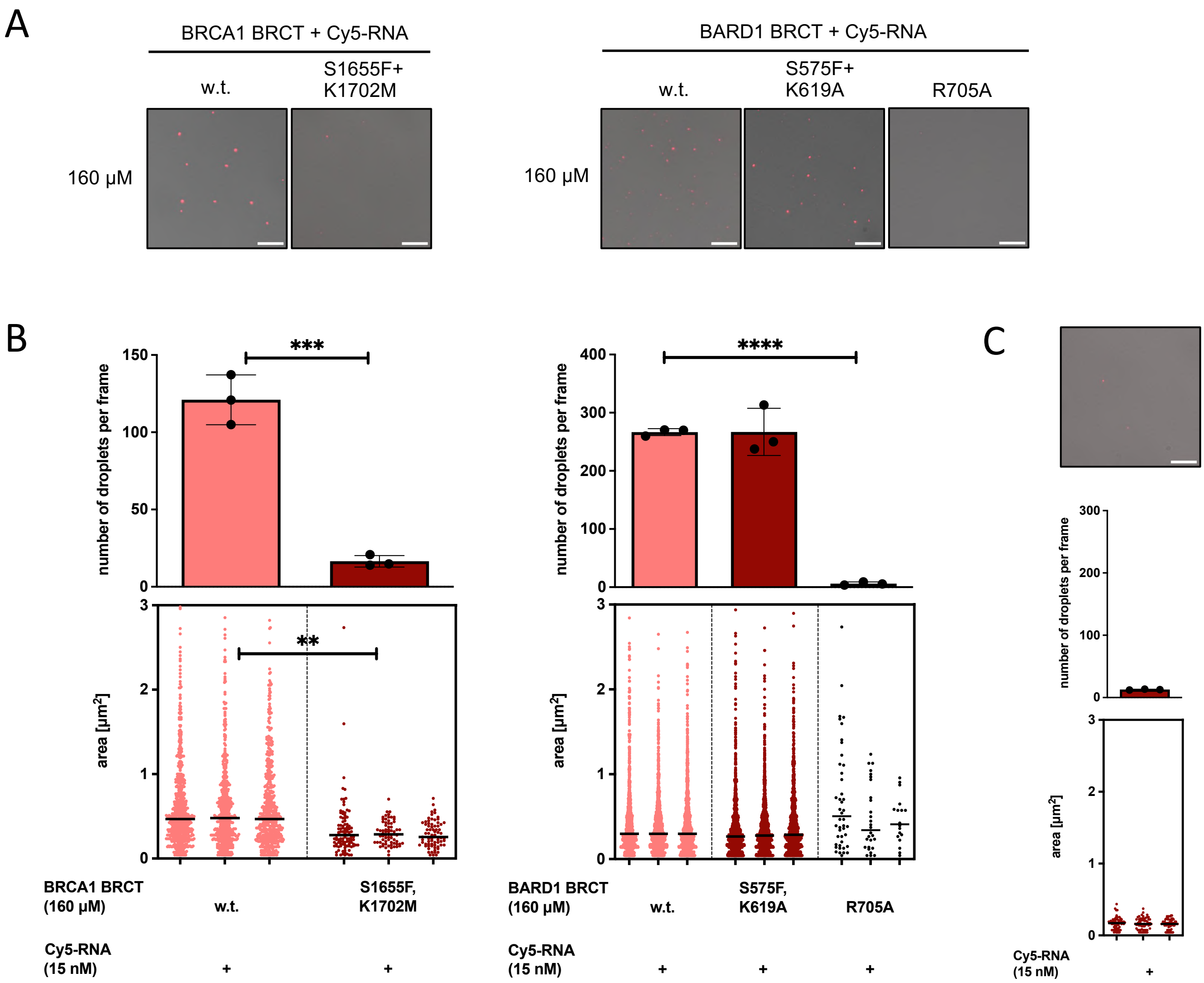

Supplementary figure 16

Klapstova *et al.*,

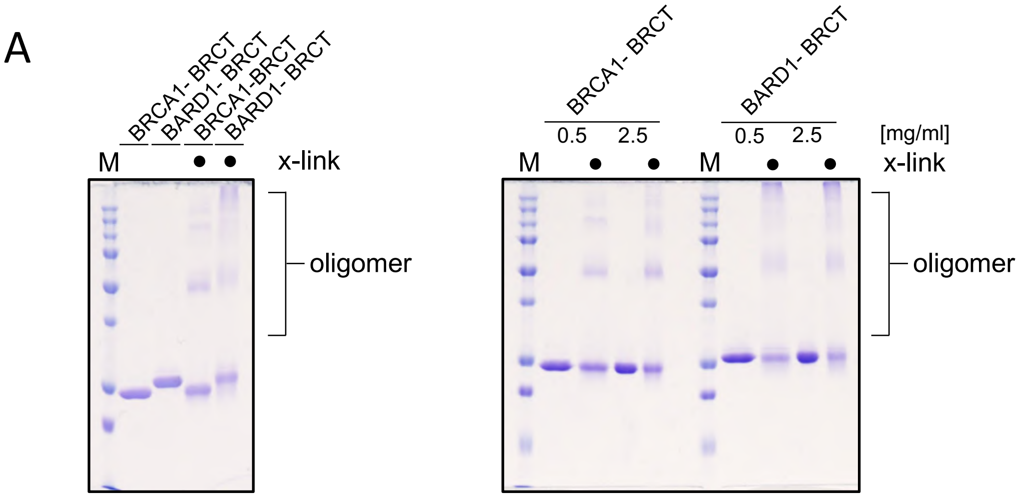

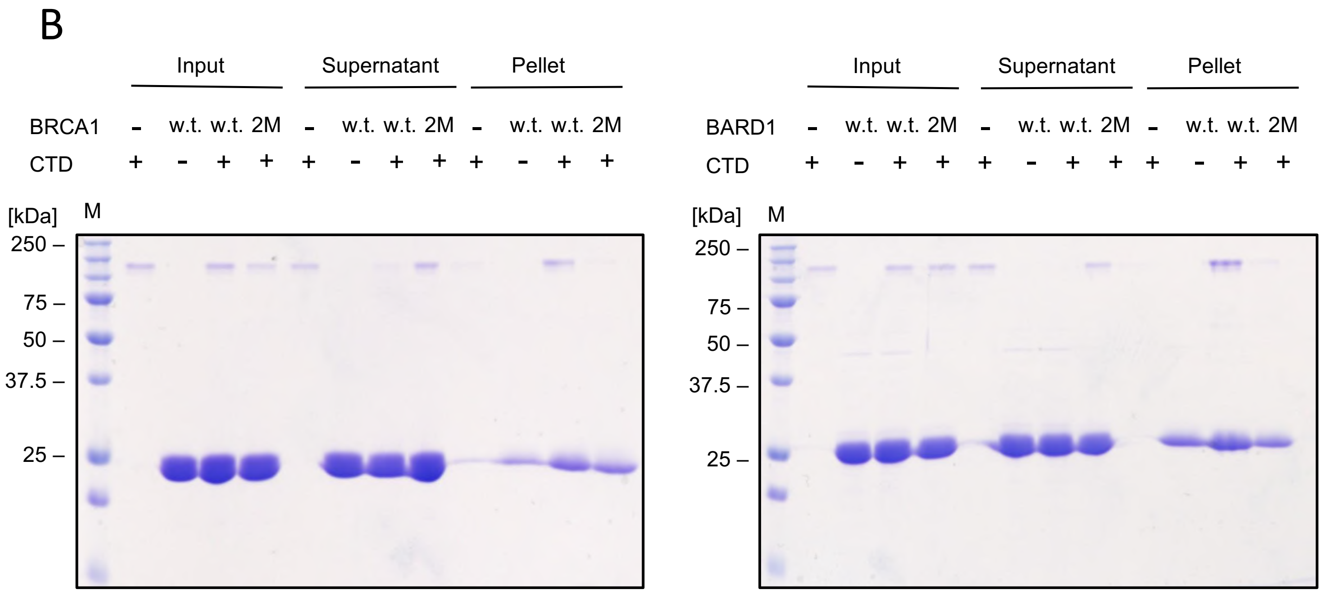

Supplementary figure 17 **Klapstova *et al*.,**

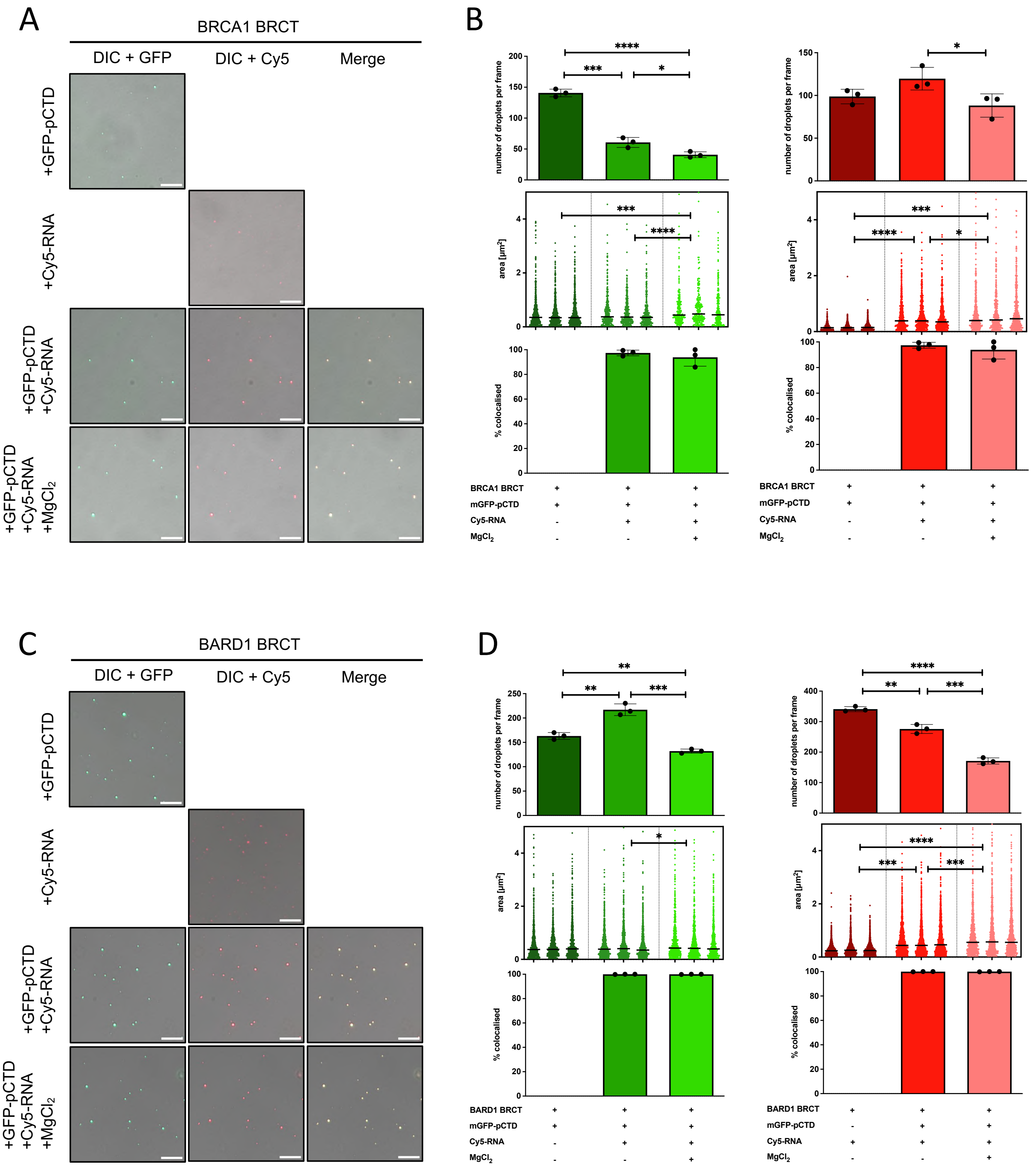

Supplementary figure 18

Klapstova *et al.*,

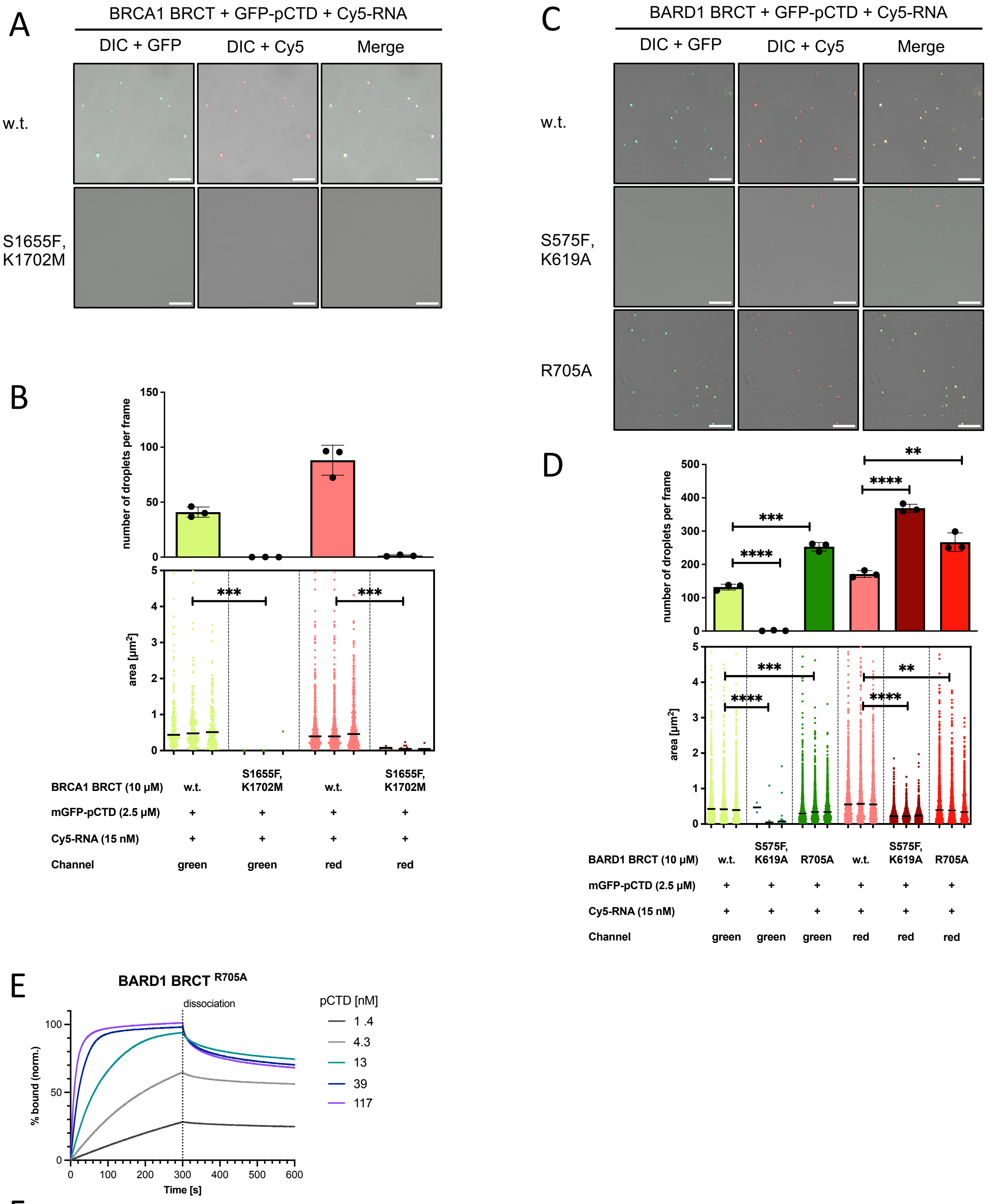

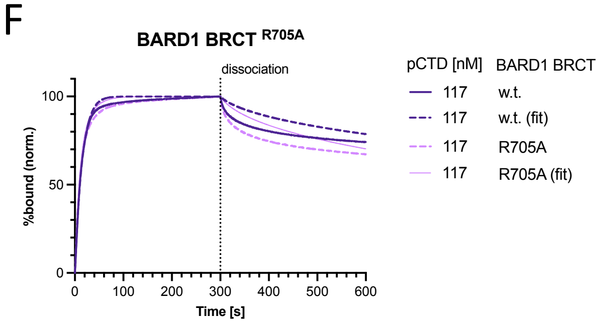

BARD1 BRCT	K_D (M)	K_D error	Full R^2	k_{as} (1/Ms)	k_{dis} (1/s)
**w.t.**	1,93E^{-07}	2,88E^{-08}	0,9730	2,66E^{+05}	5,12E^{-02}
**R705A**	3,05E^{-07}	3,07E^{-08}	0,9893	2,17E^{+05}	6,63E^{-02}

Supplementary figure 19 **Klapstova *et al.*,**

A BRCA1 BRCT

		E1682		E1754	S1755
Mytilus_edulis/2266-2571	2302	K	2364	Q	S
Bombus_vosnesenskii/1560-1794	1596	K	1665	C	R
Strongylocentrotus_purpuratus/2411-2641	2448	A	2520	T	V
Salmo_salar/1380-1588	1416	S	1488	T	T
Xenopus_laevis/1369-1579	1405	D	1477	L	G
Mus_musculus/1589-1812	1625	E	1697	E	S
Homo_sapiens/1646-1863	1682	E	1754	E	S
Felis_catus/1647-1871	1683	E	1755	E	S
Anolis_carolinensis/1487-1697	1523	E	1595	E	S
Gallus_gallus/1537-1749	1573	D	1645	Q	S
Latimeria_chalumnae/1652-1873	1688	S	1760	Q	M
Chiloscyllium_punctatum/1785-1999	1821	S	1893	K	T

B BARD1 BRCT

		E587		E665		S711		K754
Chiloscyllium_punctatum/543-770	577	K	657	E	703	S	746	K
Xenopus_laevis/546-769	580	K	658	Q	704	S	747	K
Latimeria_chalumnae/549-772	582	K	660	D	706	S	749	K
Mus_musculus/542-765	575	K	653	E	699	S	742	K
Homo_sapiens/554-777	587	E	665	E	711	S	754	K
Felis_catus/549-772	582	E	660	E	706	S	749	K
Anolis_carolinensis/546-769	579	K	657	E	703	N	746	K
Gallus_gallus/589-812	623	K	701	E	747	S	790	K
Danio_rerio/429-652	460	R	539	G	585	S	628	K
Betta_splendens/549-783	588	K	666	C	712	S	755	K
Nephila_pilipes/316-550	347	K	436	E	484	T	522	H
Strongylocentrotus_purpuratus/636-877	673	T	762	Q	810	D	853	K
Mytilus_edulis/290-522	322	K	411	K		-	498	K

Supplementary figure 20 **Klapstova *et al.*,**

A

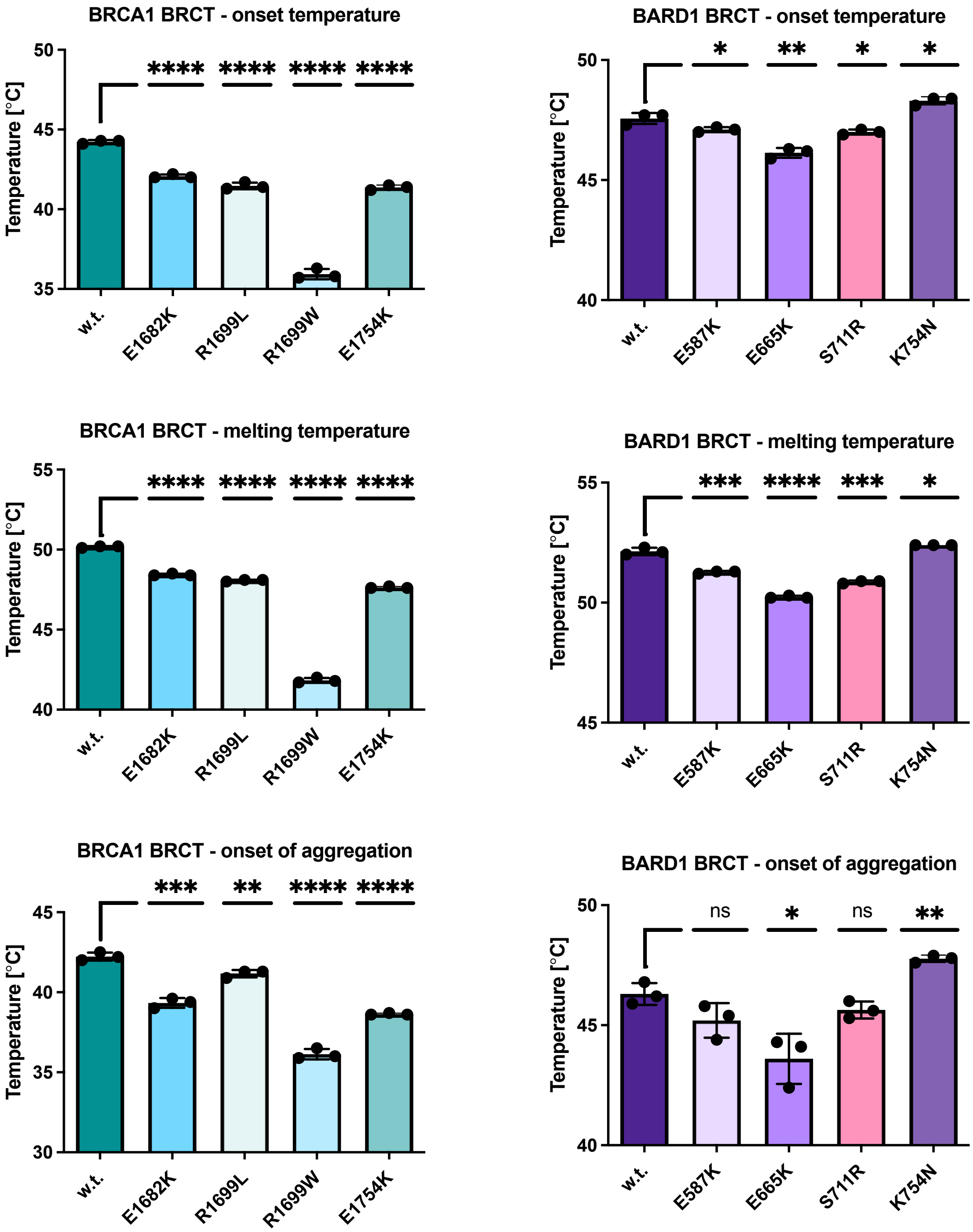

B

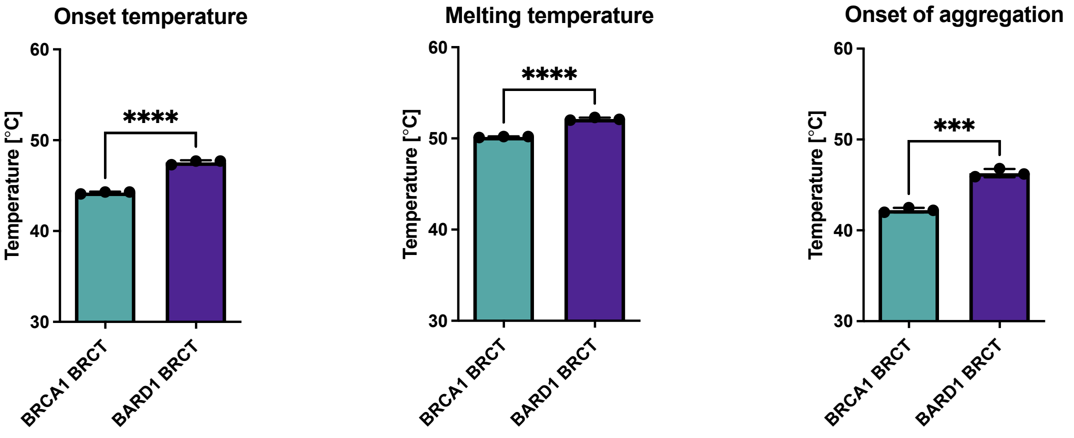

Supplementary figure 21 Klapstova *et al.*,

A

BRCA1 BRCT ^{E1754K}

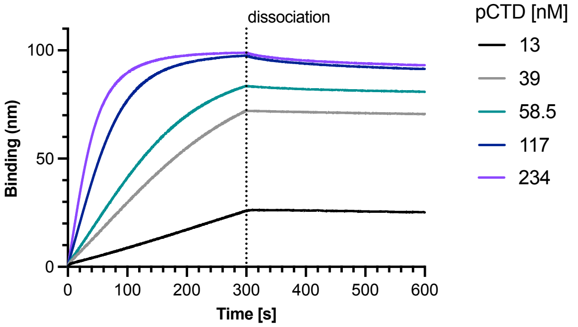

B

BRCA1 BRCT ^{E1754K}

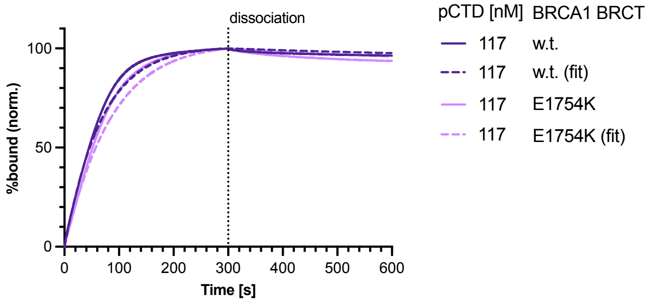

BRCA1 BRCT	K_D (M)	K_D error	Full R^2	k_{as} (1/Ms)	k_{dis} (1/s)
**w.t.**	1,98E^{-08}	9,60E^{-10}	0,9982	3,22E^{+04}	6,37E^{-04}
**E1754K**	3,05E^{-08}	1,52E^{-09}	0,9941	2,60E^{+04}	7,92E^{-04}

C

BARD1 BRCT ^{E665K}

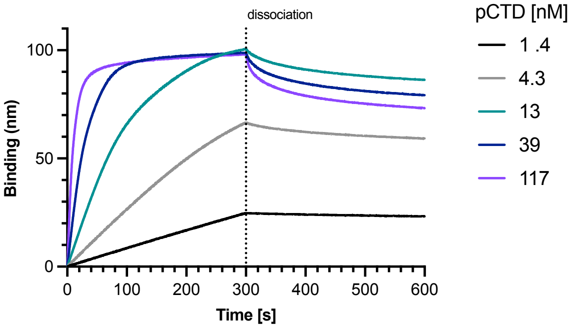

BARD1 BRCT ^{K754N}

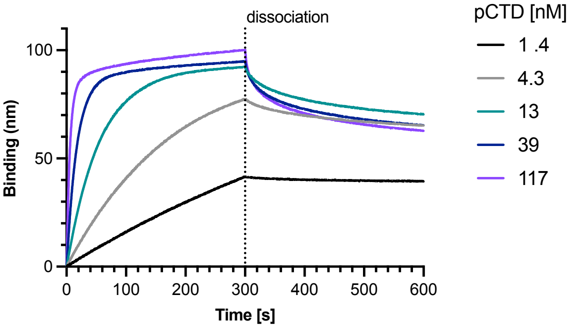

D

BARD1 BRCT ^{E665K}, BARD1 BRCT ^{K754N}

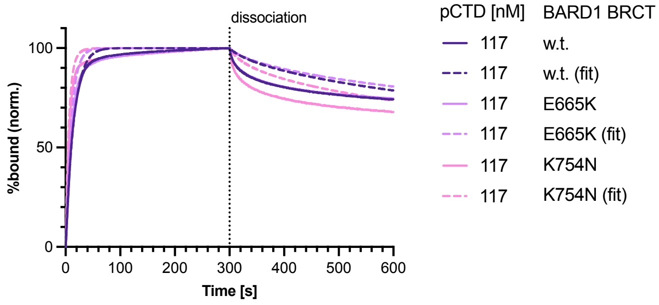

BARD1 BRCT	K_D (M)	K_D error	Full R^2	k_{as} (1/Ms)	k_{dis} (1/s)
**w.t.**	1,93E^{-07}	2,88E^{-08}	0,9730	2,66E^{+05}	5,12E^{-02}
**E665K**	3,28E^{-07}	8,26E^{-08}	0,9819	2,07E^{+05}	6,79E^{-02}
**K754N**	1,11E^{-07}	9,54E^{-09}	0,9693	3,64E^{+05}	4,03E^{-02}

Supplementary figure 22 Klapstova *et al.*,

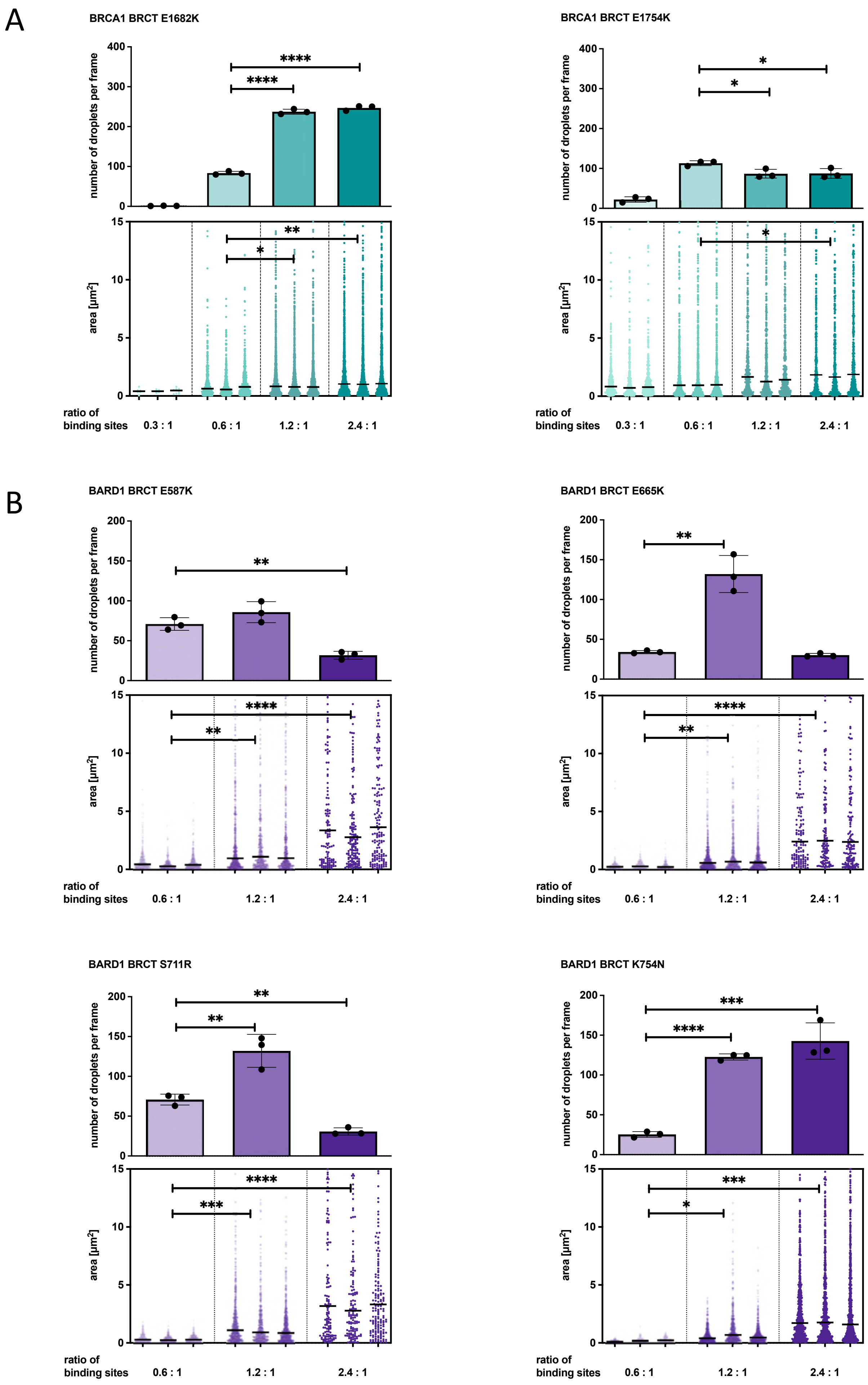

Supplementary figure 23 Klapstova *et al.*,

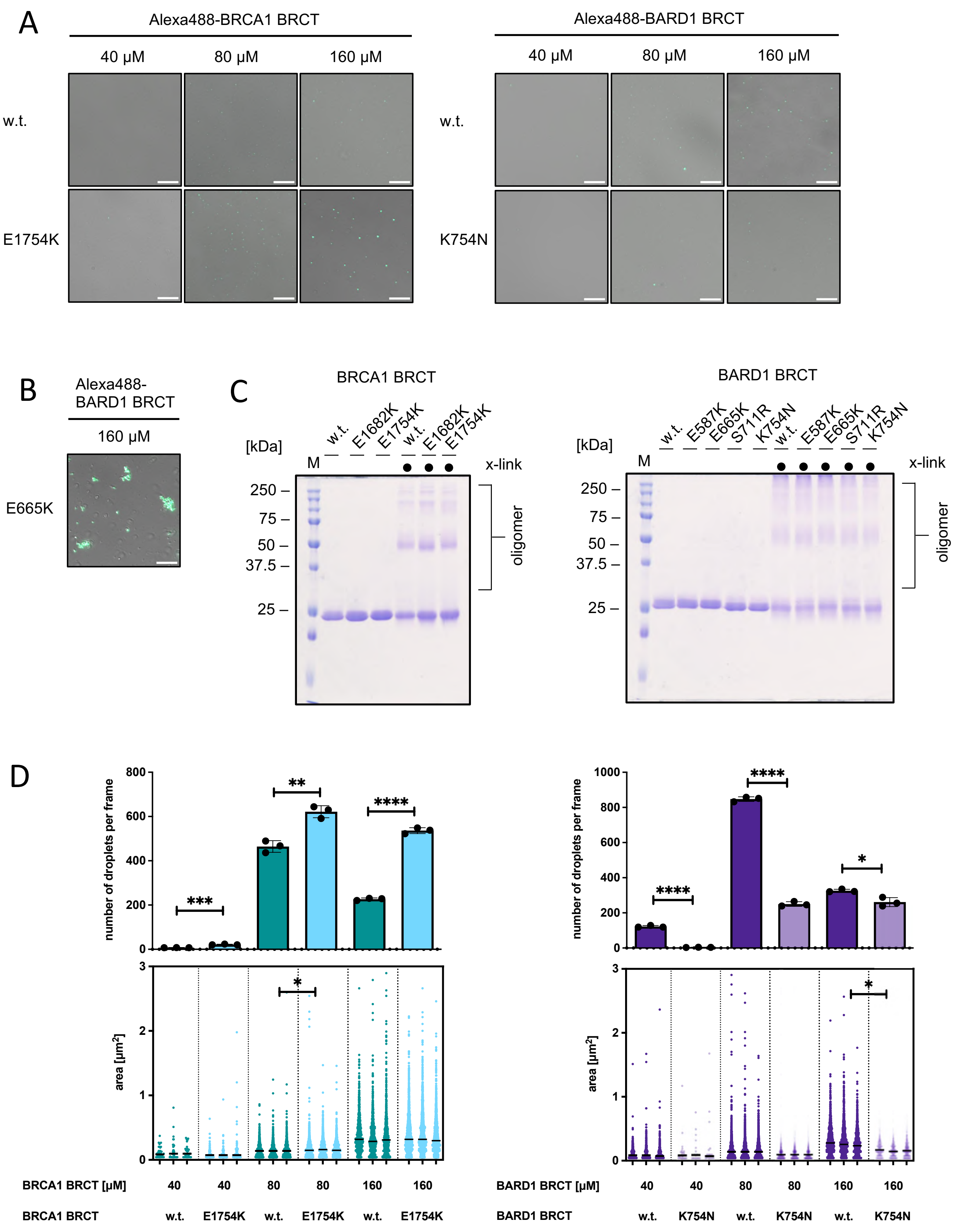

Supplementary figure 24

Klapstova *et al.*,

A

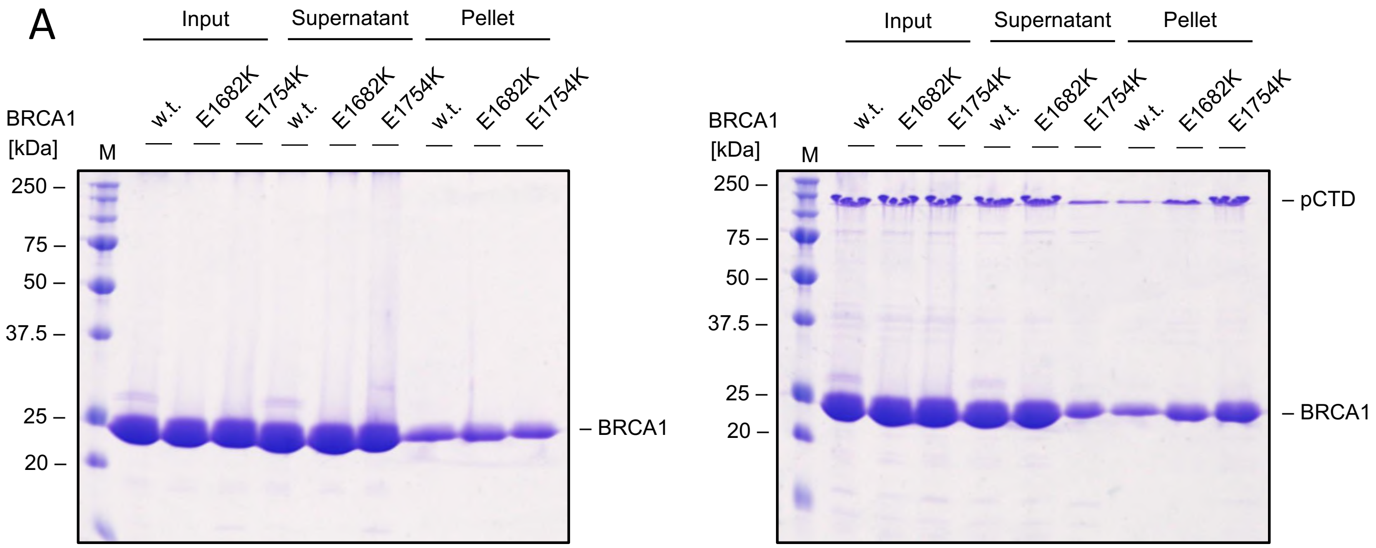

B

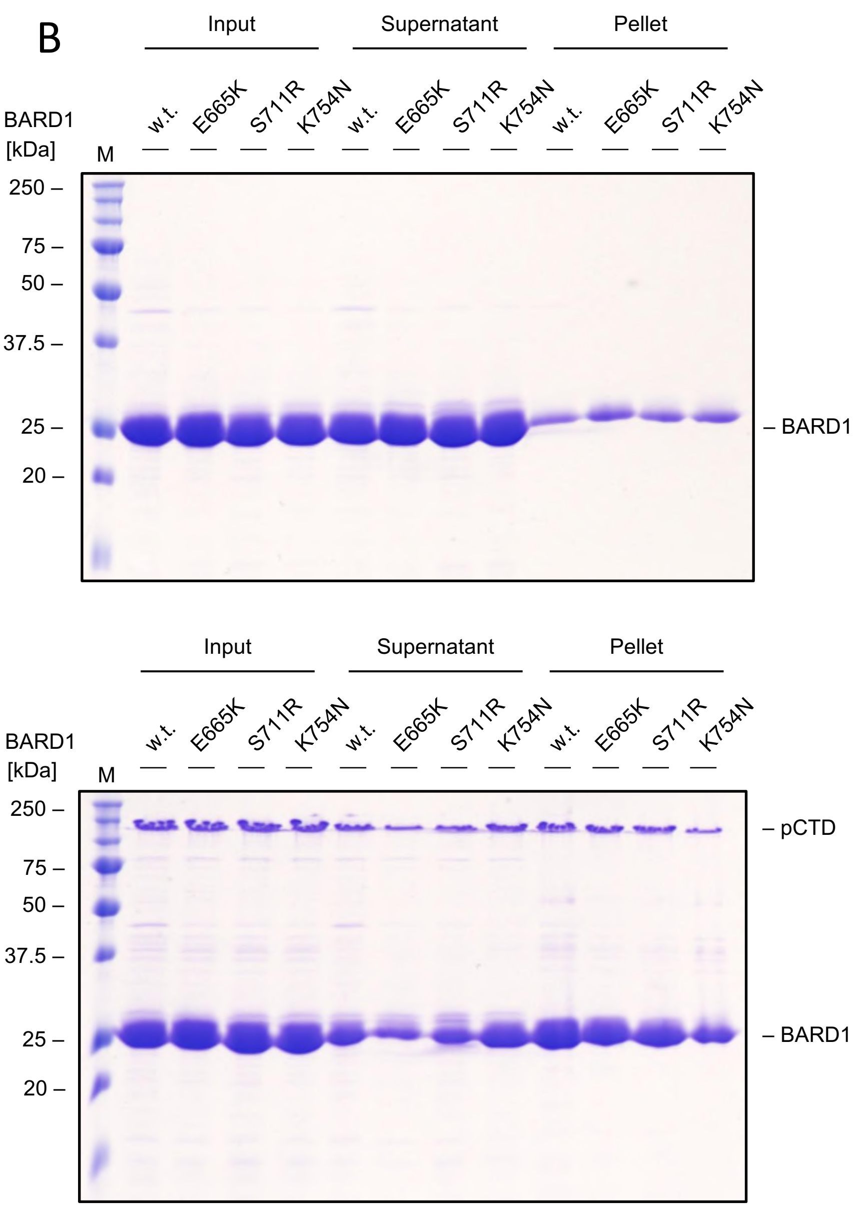

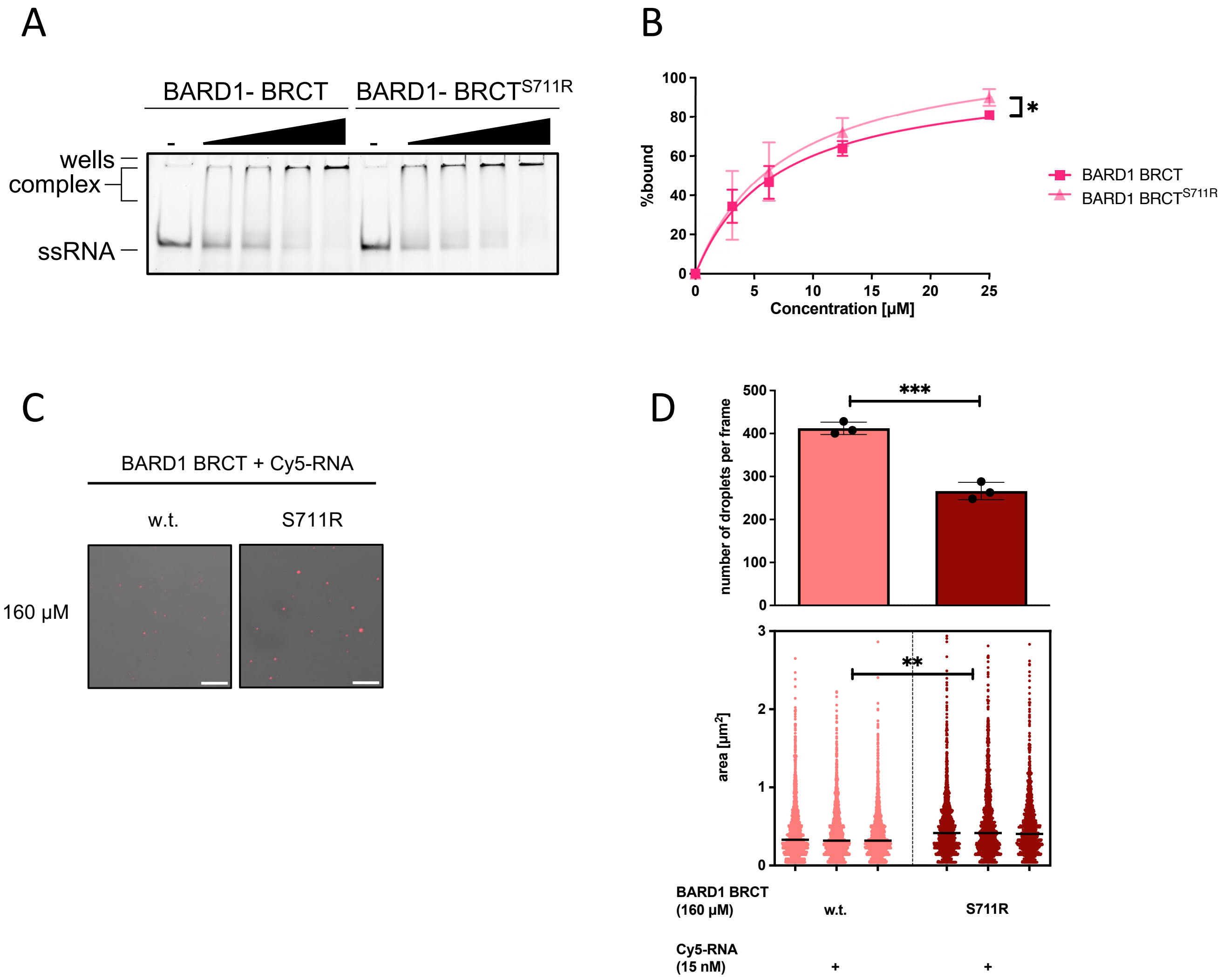
A
BARD1- BRCT
BARD1- BRCT S711R
wells
complex
ssRNA
B
100
80
60
40
20
0
%bound
0
5
10
15
20
25
Concentration [µM]
*
BARD1 BRCT
BARD1 BRCT S711R
C
BARD1 BRCT + Cy5-RNA
w.t.
S711R
160 µM
D
number of droplets per frame
500
400
300
200
100
0
***
area [µm2]
3
2
1
0
**
BARD1 BRCT (160 µM)
w.t.
S711R
Cy5-RNA (15 nM)
+
+

Supplementary figure 26

Klapstova ***et al.***,

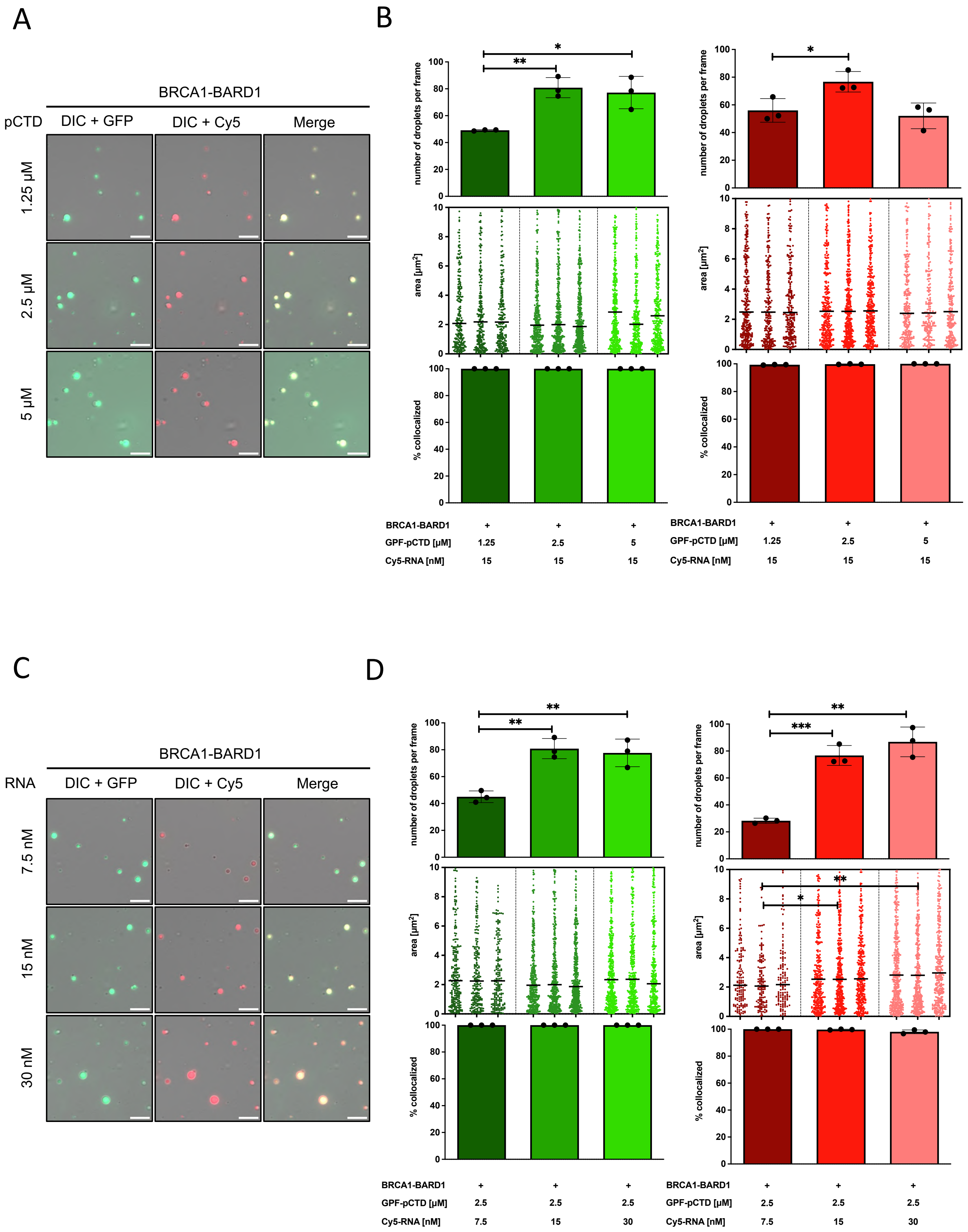

Supplementary figure 27

Klapstova ***et al.***,

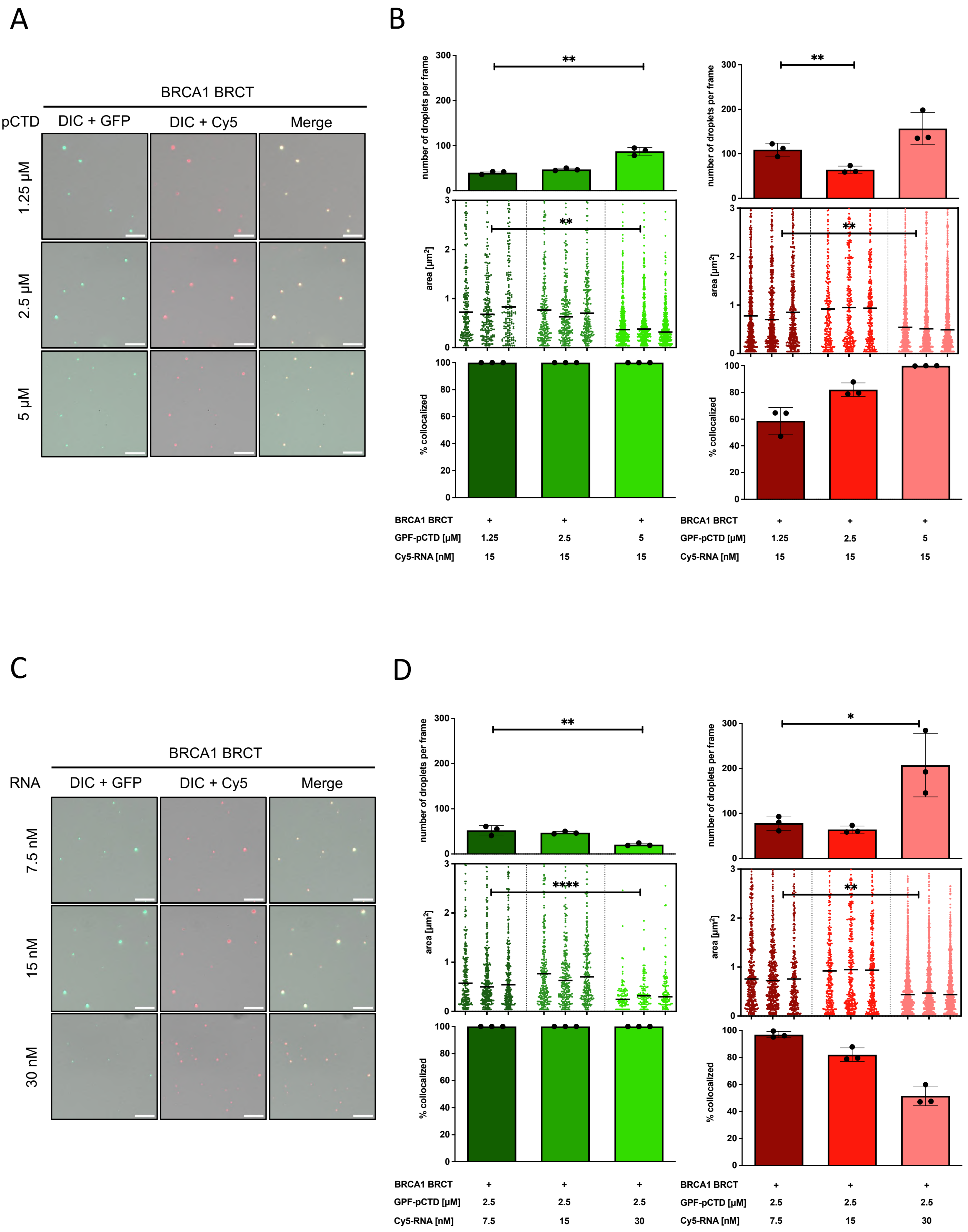

Supplementary figure 28

Klapstova *et al.*,

A

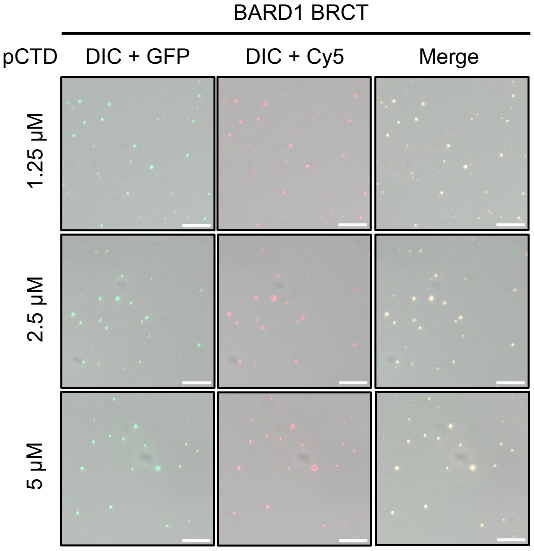

B

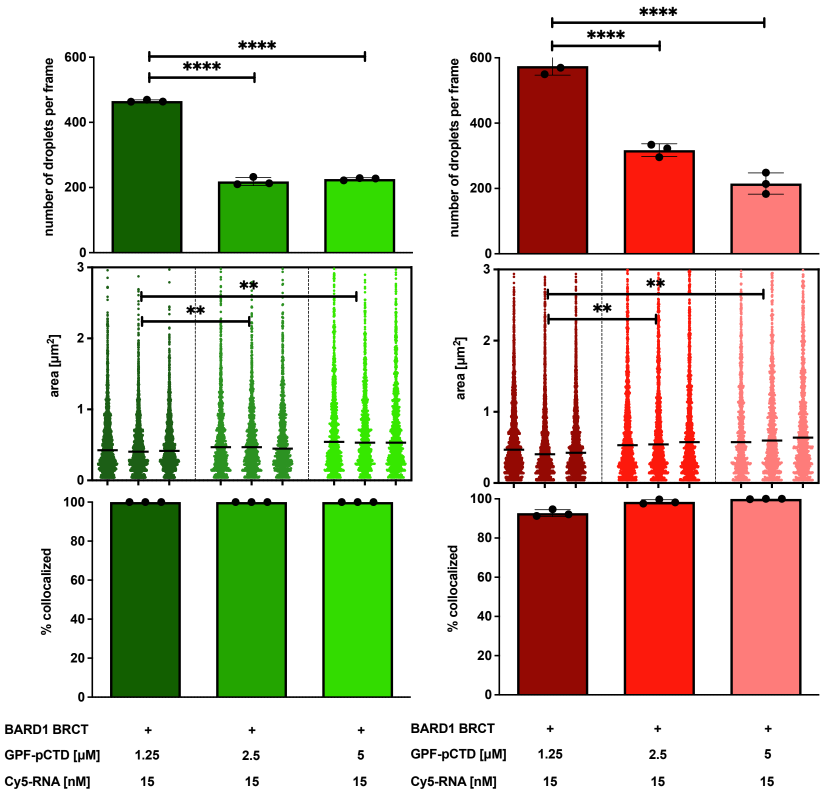

C

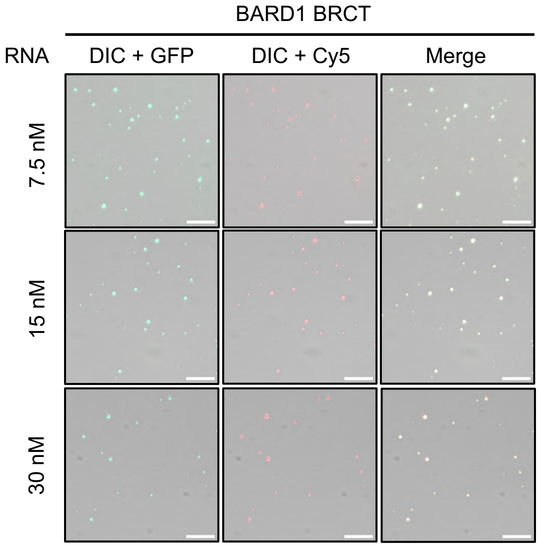

D

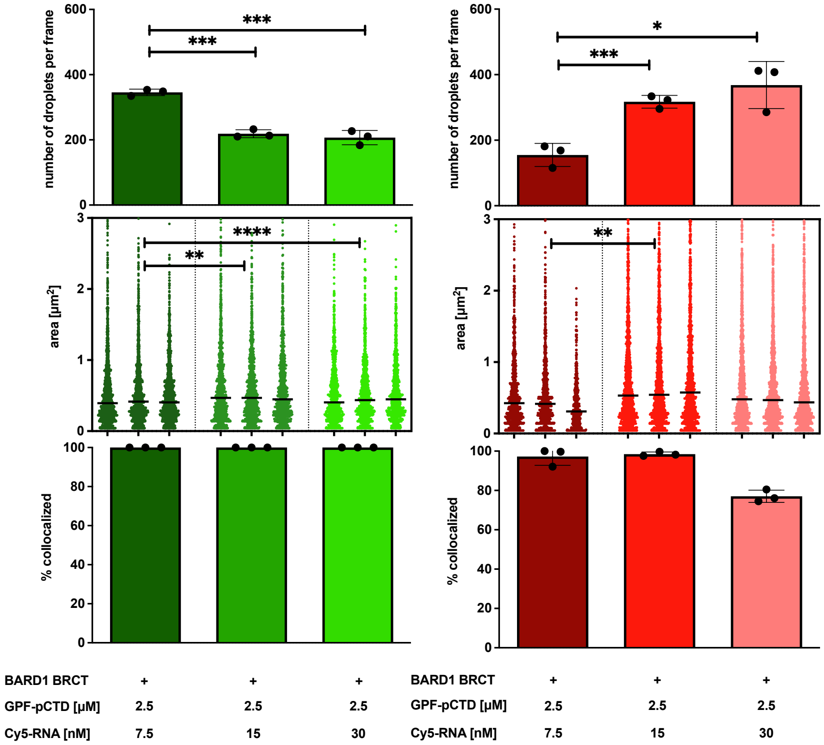

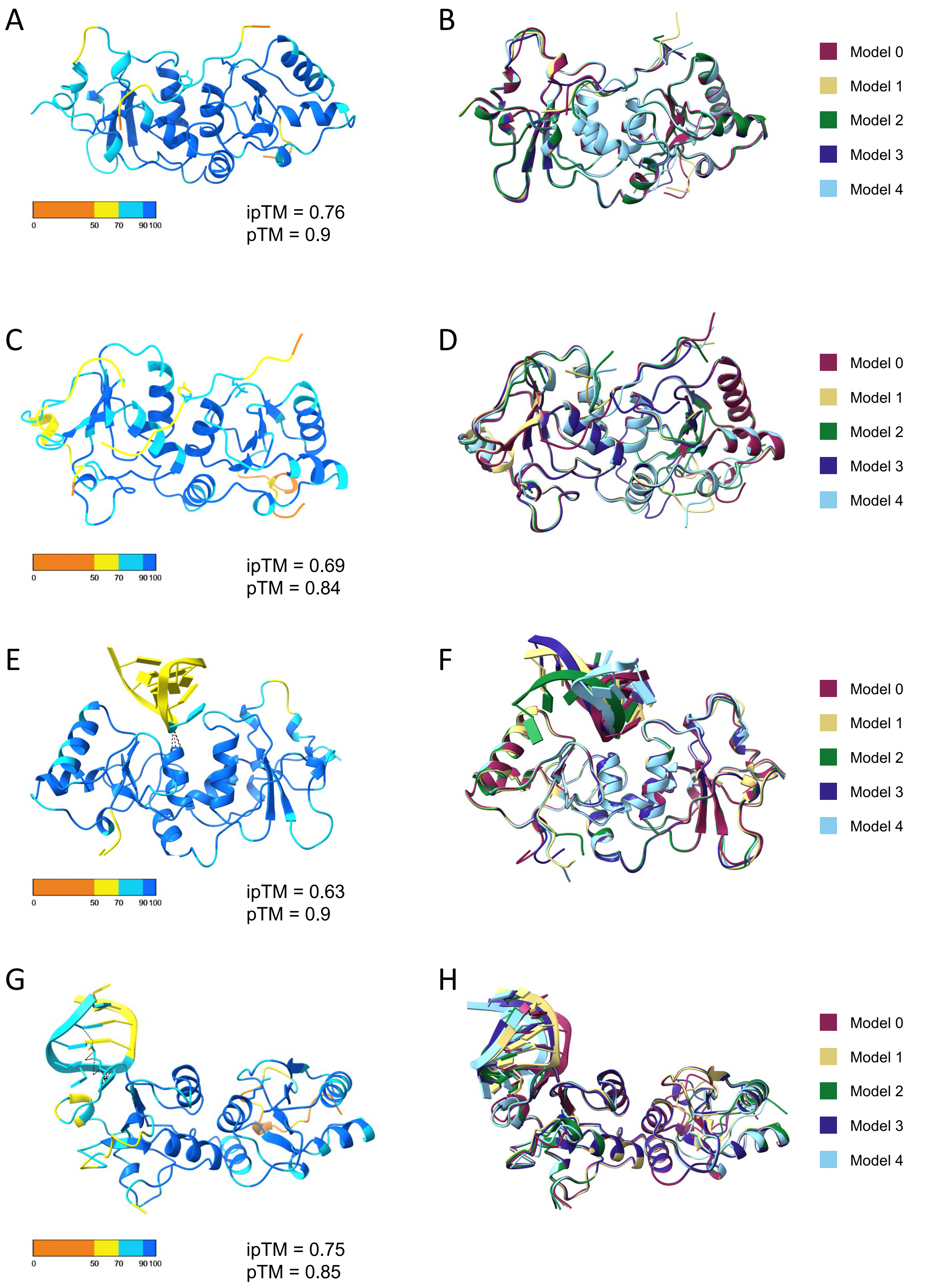
A
0
50
70
90 100
ipTM = 0.76
pTM = 0.9
B
Model 0
Model 1
Model 2
Model 3
Model 4
C
0
50
70
90 100
ipTM = 0.69
pTM = 0.84
D
Model 0
Model 1
Model 2
Model 3
Model 4
E
0
50
70
90 100
ipTM = 0.63
pTM = 0.9
F
Model 0
Model 1
Model 2
Model 3
Model 4
G
0
50
70
90 100
ipTM = 0.75
pTM = 0.85
H
Model 0
Model 1
Model 2
Model 3
Model 4
